# A Convenient Synthesis of 3,7′-Bisindole Derivatives

**DOI:** 10.3390/molecules21050638

**Published:** 2016-05-17

**Authors:** Teng Liu, Hong-You Zhu, Da-Yun Luo, Sheng-Jiao Yan, Jun Lin

**Affiliations:** 1 Key Laboratory of Medicinal Chemistry for Natural Resources, Ministry of Education, School of Chemical Science and Technology, Yunnan University, Kunming 650091, China; 15284404381@163.com (T.L.); L983189022@163.com (D.-Y.L.); 2 Guangdong Goodscend Pharm. Sci &Tech. Co., Ltd., Shantou 650091, China; hyzhu@ynu.edu.cn

**Keywords:** 3,7′-bisindoles, Michael addition, condensation reaction, heterocyclic ketene aminals

## Abstract

An efficient and convenient method to synthesize highly functionalized 3,7′-bisindole derivatives has been developed via a Michael addition and cyclic condensation reaction of heterocyclic ketene aminals (HKAs) with 2-(1*H*-indol-3-yl)cyclohexa-2,5-diene-1,4-dione derivatives in ethanol-based solvents at room temperature. This strategy provides an efficient, environmentally friendly approach for easy access to various novel 3,7′-bisindole derivatives in moderate to good yields.

## 1. Introduction

**Figure 1:**
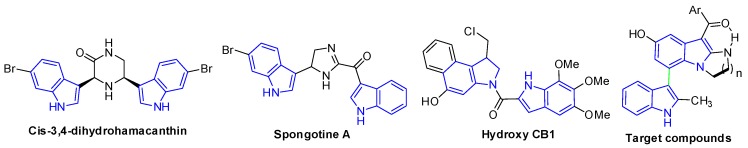
Biologically active bisindole derivatives.

Bisindole-containing systems are prevalent molecular architectures that are widely found in natural products [1,2,3,4]. Furthermore, bisindole derivatives are especially important [5,6,7,8] due to their potent biological activities, including methicillin-resistant *S*. *aureus* (MRSA) pyruvate kinase inhibitors (cis-3,4-dihydrohamacanthin and spongotine A, Figure 1) [9,10,11], antitumor agents (Hydroxy CB1, Figure 1) [12,13,14], antihistamines and antimicrobials [15], anti-inflammatories [16], antibacterials and so on [17,18]. Because of their unique biological activities, more and more synthetic strategies to generate bisindole skeletons have been developed.

Generally, Lewis acids as well as Brønsted acids are employed as catalysts to form bisindole derivatives starting from indoles reacted with carbonyl compounds and their synthetic equivalents [19,20,21,22,23,24,25,26,27,28,29]. However, the synthetic pathways of highly functionalized bisindole derivatives usually suffer from common limitations, including harsh reaction conditions, multistep reactions, use of toxic solvents, and costly catalysts or enzymes [30]. Consequently, the development of more straightforward, eco-friendly and efficient strategies is highly desirable for the synthesis of bisindoles.

**Figure 2:**
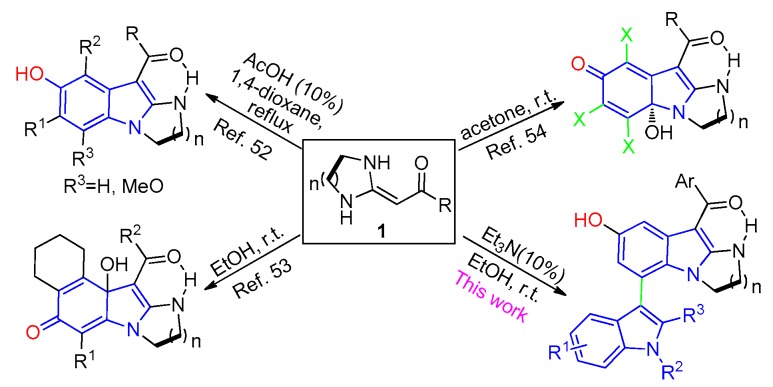
Synthesis of 3,7′-bisindole derivatives based on the HKAs.

Heterocyclic ketene aminals (HKAs) are versatile building blocks used to construct a variety of fused heterocyclic compounds [31,32,33], such as quinolones [34,35], pyridines [36,37,38,39,40,41,42], pyrroles [43,44,45,46,47], spirooxindoles [48,49], *etc.* In recent years, we have developed some protocols to synthesize different substituted indole derivatives based on HKA building blocks [50,51,52] (Figure 2). Herein, we report an efficient and concise process to construct highly functionalized 3,7′-bisindole derivatives via an environmentally friendly and highly selective one-pot protocol.

## 2. Results and Discussion

Initially, the model reaction of **1a** and **2a** with different catalysts, solvents and temperatures was studied, and the results are summarized in Table 1. Results showed that alkaline catalysts are better than acid catalysts. Furthermore, different bases have remarkable effects on the reactions. The use of triethylamine as the catalyst in ethanol at room temperature made the reaction proceed smoothly and afforded the target product **3a** in good yield (Table 1, entry 3). Carbonate catalysts, such as Na_2_CO_3_ and K_2_CO_3_, also gave product **3a** with moderate yield (Table 1, entries 4–5). However, NaOEt provided product **3a** with poor yield, which may be due to its strong basicity (Table 1, entry 6). Notably, trace product was detected when 1,8-diazabicyclo[5,4,0]undec-7ene (DBU) was employed as the catalyst (Table 1, entry 7). Next, solvent effects were examined. Most solvents had little influence and could facilitate good yield of the products, except H_2_O (Table 1, entries 8–13). Ultimately, EtOH was proved to be the best solvent (Table 1, entry 3). To gain further insight into the effects of reaction temperature, we examined 40 °C and reflux temperature. The results revealed that high temperature was adverse to the reaction (Table 1, entries 14–15). Therefore, it could be concluded that the optimum conditions for the synthesis of **3a** were EtOH as the solvent and triethylamine as the catalyst at room temperature for 12 h (Table 1, entry 3).

With the optimized conditions in hand, the substrate scope was investigated (Table 2). The results showed that the reaction was tolerant to a variety of HKAs bearing an electron-donating or an electron-withdrawing group. Furthermore, the ring size of HKA **1** has a slight effect on the reaction yield. Six- and seven-membered HKAs as substrates usually afforded superior yields to that of the five-membered HKAs. Additionally, aryl-substituted substrate **2** (R^4^ = Ph) was also tolerant to the reaction. Notably, substrate **2**, with or without a substituent group at N1 (R^3^ = Me, H), reacted smoothly with HKA **1** to provide the corresponding products **3** in moderate to good yields. Substrate **2** with a methoxy at C5 and no substituent at C2 reacted cleanly with HKA **1** to provide the corresponding product **3** in good yield. All new compounds were fully characterized using IR, HR-MS, ^1^H-NMR, ^13^C-NMR (Please see the Supplementary Materials).

**Scheme 1:**
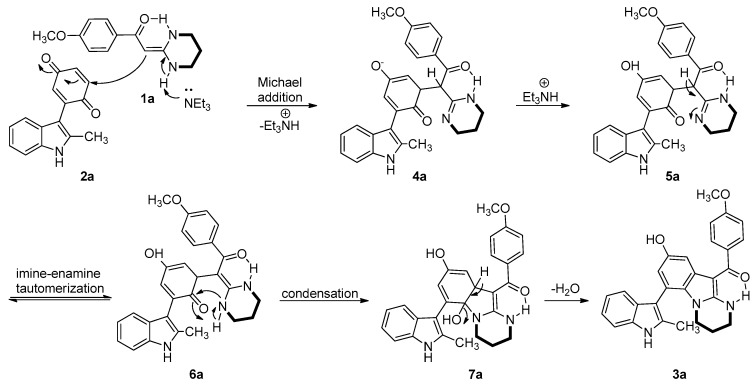
A plausible mechanism for the synthesis of **3a**.

A proposed mechanism of the base-catalyzed cyclocondensation of **1a** with **2a** is depicted in Scheme 1. Initially, HKA **1a** reacted with **2a** in the presence of Et_3_N to form intermediate **4a** by a Michael addition reaction. Intermediate **4a** was subsequently protonated to form compound **5a**. Imine-enamine tautomerization of compound **5a** then generates **6a**, which cyclizes to **7a** by intramolecular attack of the NH on the 2,4-cyclohexadienone. Loss of H_2_O from intermediate **7a** then provides the final product **3a**.

## 3. Experimental Section

### 3.1. General Information and Materials

All compounds were fully characterized by spectroscopic data. The NMR spectra were recorded on a Bruker DRX400 and DRX500 (Fällanden, Zürich, Switzerland). Chemical shifts (δ) are expressed in ppm, *J* values are given in Hz, and deuterated DMSO-*d*_6_ and CDCl_3_ were used as solvent. IR spectra were recorded on a FT-IR Thermo Nicolet Avatar 360 (Boston, MA, USA) using a KBr pellet. The reactions were monitored by thin layer chromatography (TLC) using silica gel GF254. The melting points were determined on a XT-4A melting point apparatus and are uncorrected. HRMs were performed on an Agilent LC/Msd TOF instrument (Palo Alto, CA, USA). All chemicals and solvents were used as received without further purification unless otherwise stated. Raw materials **1** and **2** were prepared according to the literature [53,54,55,56].

### 3.2. General Procedure for for the Preparation of the 3,7′-Bisindole Derivatives ***3a**–**3w***

Et_3_N (0.1 equiv) was added to a mixture of HKAs **1** (0.1 mmol) and compound **2** (0.11 mmol) in ethanol, and the mixture was stirred at room temperature until the HKAs **1** were completely consumed. Then, the solution was concentrated under reduced pressure and purified by flash column chromatography (petroleum ether/EtOAc = 6/1) to afford the corresponding products **3a**–**3w** with 65%–91% yield. The products were further identified by FT-IR, NMR, and HRMS, and were in good agreement with the assigned structures.

*(8-Hydroxy-6-(2-methyl-1H-indol-3-yl)-1,2,3,4-tetrahydro-pyrimido[1,2-a]indol-10-yl)(4-methoxyphenyl)methanone* (**3a**). Yellow solid, yield 91%; Mp 221.5–222.5 °C; ^1^H-NMR (DMSO-*d*_6_, 500 MHz) δ 10.92 (br, 1H, NH), 8.51 (br, 1H, NH), 8.23 (s, 1H, ArH), 7.58 (d, *J* = 8.0 Hz, 2H, ArH), 7.28–7.30 (m, 2H, ArH), 7.06 (d, *J* = 8.0 Hz, 2H, ArH), 6.97–7.03 (m, 1H, ArH), 6.91–6.94 (m, 2H, ArH), 6.50 (br, 1H, OH), 3.91–3.95 (m, 2H, NCH_2_), 3.85 (s, 3H, OCH_3_), 3.46–3.50 (m, 2H, CH_2_N), 2.30 (s, 3H, CH_3_), 2.07–2.11 (m, 2H, CH_2_); ^13^C-NMR (DMSO-*d*_6_, 125 MHz) δ 186.8, 160.7, 153.1, 150.3, 135.6, 135.5, 133.1, 129.4, 129.4, 128.9, 128.8, 125.3, 120.2, 118.9, 118.7, 114.7, 113.9, 113.9, 113.9, 110.6, 110.5, 105.4, 94.9, 55.6, 39.3, 38.1, 20.6, 13.0; IR (KBr) 3439, 3230, 2904, 2586, 1722, 1599, 1514, 1333, 1223, 752 cm^−1^; HRMS (ESI-TOF): *m*/*z* calcd for C_28_H_26_N_3_O_3_ [M + H]^+^, 452.1969; found, 452.1947.

*(8-Hydroxy-6-(2-methyl-1H-indol-3-yl)-1,2,3,4-tetrahydro-pyrimido[1,2-a]indol-10-yl)(p-tolyl)methanone* (**3b**). Yellow solid, yield 88%; Mp 228–230 °C; ^1^H-NMR (DMSO-*d*_6_, 500 MHz) *δ* 10.82 (br, 1H, NH), 8.44 (br, 1H, NH), 8.19 (s, 1H, ArH), 7.41 (d, *J* = 7.5 Hz, 2H, ArH), 7.23–7.32 (m, 3H, ArH), 7.21 (d, *J* = 7.5 Hz, 1H, ArH), 6.98 (t, *J* = 7.5 Hz, 1H, ArH), 6.85–6.92 (m, 2H, ArH), 6.32 (br, 1H, OH), 3.82–3.89 (m, 2H, NCH_2_), 3.42–3.46 (m, 2H, CH_2_N), 2.37 (s, 3H, CH_3_), 2.24 (s, 3H, ArCH_3_), 2.04–2.08 (m, 2H, CH_2_); ^13^C-NMR (DMSO-*d*_6_, 125 MHz) δ 187.6, 153.3, 150.1, 139.8, 139.7, 135.5, 133.3, 129.3, 129.3, 129.0, 128.7, 127.3, 127.3, 125.1, 120.4, 118.9, 118.7, 115.1, 110.8, 110.5, 110.2, 105.4, 95.2, 39.5, 38.0, 21.4, 20.3, 12.7; IR (KBr) 3394, 3053, 2928, 2316, 1728, 1591, 1443, 1335, 1171, 750 cm^−1^; HRMS (ESI-TOF): *m*/*z* calcd for C_28_H_26_N_3_O_2_ [M + H]^+^, 436.2020; found, 436.2005.

*(8-Hydroxy-6-(2-methyl-1H-indol-3-yl)-1,2,3,4-tetrahydro-pyrimido[1,2-a]indol-10-yl)(phenyl)methanone* (**3c**). Yellow solid, yield 82%; Mp 317–318.5 °C; ^1^H-NMR (DMSO-*d*_6_, 500 MHz) *δ* 10.95 (br, 1H, NH), 8.60 (br, 1H, NH), 8.19 (s, 1H, ArH), 7.54–7.58 (m, 5H, ArH), 7.30 (t, *J* = 7.5 Hz, 2H, ArH), 6.98–7.04 (m, 1H, ArH), 6.90–6.98 (m, 2H, ArH), 6.34 (br, 1H, OH), 3.92–3.96 (m, 2H, NCH_2_), 3.47–3.51 (m, 2H, CH_2_N), 2.30 (s, 3H, CH_3_), 2.08–2.12 (m, 2H, CH_2_); ^13^C-NMR (DMSO-*d_6_*, 125 MHz) *δ* 187.2, 153.2, 150.3, 143.2, 135.7, 133.2, 129.8, 128.9, 128.7, 128.7, 128.7, 127.3, 127.3, 125.2, 120.2, 118.9, 118.7, 114.9, 110.7, 110.6, 110.5, 105.4, 95.0, 39.3, 38.1, 20.6, 13.0; IR (KBr) 3323, 3055, 2972, 2866, 2314, 1726, 1614, 1529, 1319, 1174, 746 cm^−1^; HRMS (ESI-TOF): *m*/*z* calcd for C_27_H_24_N_3_O_2_ [M + H]^+^, 422.1863; found, 422.1871.

*(4-Chlorophenyl)(8-hydroxy-6-(2-methyl-1H-indol-3-yl)-1,2,3,4-tetrahydropyrimido[1,2-a]indol-10-yl)methanone* (**3d**). Yellow solid, yield 89%; Mp 199.0–201.5 °C; ^1^H-NMR (DMSO-*d*_6_, 500 MHz) δ 10.91 (br, 1H, NH), 8.55 (br, 1H, NH), 8.27–8.32 (m, 1H, ArH), 7.55–7.59 (m, 4H, ArH), 7.23–7.31 (m, 2H, ArH), 6.96–7.02 (m, 1H, ArH), 6.87–6.96 (m, 2H, ArH), 6.32 (br, 1H, OH), 3.91–3.95 (m, 2H, NCH_2_), 3.42–3.46 (m, 2H, CH_2_N), 2.27 (s, 3H, CH_3_), 2.08–2.12 (m, 2H, CH_2_); ^13^C-NMR (DMSO-*d*_6_, 125 MHz) δ 185.6, 153.2, 150.5, 141.8, 135.6, 134.3, 133.1, 129.3, 129.3, 129.3, 128.9, 128.9, 128.9, 128.9, 124.9, 120.2, 118.9, 118.7, 115.0, 110.7, 110.5, 105.1, 95.0, 39.4, 38.1, 20.5, 13.0; IR (KBr) 3400, 3063, 2951, 2866, 2349, 1680, 1616, 1527, 1331, 750 cm^−1^; HRMS (ESI-TOF): *m/z* calcd for C_27_H_23_ClN_3_O_2_ [M + H]^+^, 456.1473; found, 456.1459.

*(2-Chlorophenyl)(8-hydroxy-6-(2-methyl-1H-indol-3-yl)-1,2,3,4-tetrahydropyrimido[1,2-a]indol-10-yl)methanone* (**3e**). Yellow solid, yield 86%; Mp 301–303 °C; ^1^H-NMR (DMSO-*d*_6_, 500 MHz) δ 10.94 (br, 1H, NH), 8.52 (br, 1H, NH), 8.07 (s, 1H, ArH), 7.61 (d, *J* = 1.0 Hz, 1H, ArH), 7.47–7.61 (m, 2H, ArH), 7.34–7.39 (m, 1H, ArH), 7.30 (d, *J* = 8.0 Hz, 1H, ArH), 7.26 (d, *J* = 7.5 Hz, 1H, ArH), 6.96–7.03 (m, 1H, ArH), 6.88–6.95 (m, 2H, ArH), 5.71 (br, 1H, OH), 3.90–3.94 (m, 2H, NCH_2_), 3.48–3.52 (m, 2H, CH_2_N), 2.26 (s, 3H, CH_3_), 2.08–2.12 (m, 2H, CH_2_); ^13^C-NMR (DMSO-*d*_6_, 125 MHz) *δ* 183.7, 152.8, 150.5, 142.3, 135.7, 133.2, 130.4, 130.1, 129.6, 129.0, 128.9, 128.2, 128.1, 125.0, 120.3, 118.8, 118.7, 115.1, 110.7, 110.7, 110.4, 104.7, 95.7, 39.3, 38.1, 20.4, 13.0; IR KBr) 3342, 3061, 2966, 2868, 1726, 1618, 1531, 1429, 1329, 1176, 748 cm^−1^; HRMS (ESI-TOF): *m/z* calcd for C_27_H_23_ClN_3_O_2_ [M + H]^+^, 456.1473; found, 456.1462.

*(4-Fluorophenyl)(8-hydroxy-6-(2-methyl-1H-indol-3-yl)-1,2,3,4-tetrahydropyrimido[1,2-a]indol-10-yl)methanone* (**3f**). Yellow solid, yield 89%; Mp 247–248.5 °C; ^1^H-NMR (DMSO-*d*_6_, 500 MHz) *δ* 10.93 (br, 1H, NH), 8.54 (br, 1H, NH), 8.27 (s, 1H, ArH), 7.58–7.65 (m, 2H, ArH), 7.34 (t, *J* = 9.0 Hz, 2H, ArH), 7.28 (t, *J* = 9.0 Hz, 2H, ArH), 7.00 (t, *J* = 7.5 Hz, 1H, ArH), 6.87–6.97 (m, 2H, ArH), 6.31 (br, 1H, OH), 3.90–3.97 (m, 2H, NCH_2_), 3.47–3.51 (m, 2H, CH_2_N), 2.28 (s, 3H, CH_3_), 2.08–2.12 (m, 2H, CH_2_); ^13^C-NMR (DMSO-*d*_6_, 125 MHz) δ 185.9, 164.0, 162.1, 153.1, 150.4, 139.6, 135.6, 133.1, 129.7, 129.7, 128.9, 125.1, 120.2, 118.9, 118.7, 115.7, 115.5, 114.9, 110.7, 110.7, 110.5, 105.1, 95.0, 39.4, 38.1, 20.5, 13.0; IR (KBr) 3394, 3053, 2928, 2860, 2316, 1720, 1591, 1441, 1335, 1170, 750 cm^−1^; HRMS (ESI-TOF): *m/z* calcd for C_27_H_23_FN_3_O_2_ [M + H]^+^, 440.1769; found, 440.1775.

*(9-Hydroxy-7-(2-methyl-1H-indol-3-yl)-2,3,4,5-tetrahydro-1H-[1,3]diazepino[1,2-a]indol-11-yl)(4-methoxyphenyl)methan-one* (**3g**). Yellow solid, yield 87%; Mp 179.5–182 °C; ^1^H-NMR (DMSO-*d*_6_, 500 MHz) *δ* 10.93 (br, 1H, NH), 8.96 (br, 1H, NH), 8.32 (s, 1H, ArH), 7.59 (d, *J* = 7.6 Hz, 2H, ArH), 7.29 (d, *J* = 7.5 Hz, 1H, ArH), 7.26 (d, *J* = 7.4 Hz, 1H, ArH), 7.12 (s, 1H, ArH), 7.07 (d, *J* = 7.7 Hz, 2H, ArH), 7.00 (m, 1H, ArH), 6.91 (m, 1H, ArH), 6.45 (br, 1H, OH), 4.03–4.07 (m, 2H, NCH_2_), 3.86 (s, 3H, OCH_3_), 3.41–3.45 (m, 2H, CH_2_N), 2.29 (s, 3H, CH_3_), 1.87–1.97 (m, 4H, CH_2_CH_2_); ^13^C-NMR (DMSO-*d*_6_, 125 MHz) *δ* 188.3, 161.0, 160.0, 150.5, 135.6, 135.0, 133.2, 129.7, 129.7, 129.5, 128.9, 125.7, 120.2, 118.9, 118.7, 115.7, 113.9, 113.9, 112.2, 110.6, 105.3, 97.7, 55.6, 45.5, 45.1, 29.3, 27.0, 13.0; IR (KBr) 3396, 3063, 2928, 2850, 2351, 1726, 1593, 1444, 1313, 1250, 1167, 1022, 744 cm^−1^; HRMS (ESI-TOF): *m/z* calcd for C_29_H_28_N_3_O_3_ [M + H]^+^, 466.2125; found, 466.2145.

*(9-Hydroxy-7-(2-methyl-1H-indol-3-yl)-2,3,4,5-tetrahydro-1H-[1,3]diazepino[1,2-a]indol-11-yl)(p-tolyl)methanone* (**3h**). Yellow solid, yield 84%; Mp 242–244 °C; ^1^H-NMR (DMSO-*d*_6_, 500 MHz) δ 10.92 (br, 1H, NH), 9.05 (br, 1H, NH), 8.23 (s, 1H, ArH), 7.49 (d, *J* = 7.7 Hz, 2H, ArH), 7.33 (d, *J* = 7.6 Hz, 2H, ArH), 7.29 (d, *J* = 7.9 Hz, 1H, ArH), 7.25 (d, *J* = 7.8 Hz, 1H, ArH), 7.11 (s, 1H, ArH), 6.96–7.03 (m, 1H, ArH), 6.91 (t, *J* = 7.3 Hz, 1H, ArH), 6.34 (br, 1H, OH), 4.04–4.11 (m, 2H, NCH_2_), 3.39–3.43 (m, 2H, CH_2_N), 2.43 (s, 3H, ArCH_3_), 2.28 (s, 3H, CH_3_), 1.88–1.97 (m, 4H, CH_2_CH_2_); ^13^C-NMR (DMSO-*d*_6_, 125 MHz) δ 188.8, 160.0, 150.5, 139.9, 139.8, 135.6, 133.2, 129.7, 129.2, 129.2, 128.9, 127.7, 127.7, 125.6, 120.2, 118.9, 118.7, 115.8, 112.2, 110.6, 110.5, 105.4, 97.6, 45.5, 45.0, 29.2, 26.9, 21.5, 13.0; IR (KBr) 3394, 3053, 2926, 2858, 2314, 1726, 1593, 1446, 1335, 1169, 748 cm^−1^; HRMS (ESI-TOF): *m/z* calcd for C_29_H_28_N_3_O_2_ [M + H]^+^, 450.2176; found, 450.2184.

*(9-Hydroxy-7-(2-methyl-1H-indol-3-yl)-2,3,4,5-tetrahydro-1H-[1,3]diazepino[1,2-a]indol-11-yl)(phenyl)methanone* (**3i**). Yellow solid, yield 83%; Mp 289–290 °C; ^1^H-NMR (DMSO-*d*_6_, 500 MHz) δ 10.92 (br, 1H, NH), 9.10 (br, 1H, NH), 8.21 (s, 1H, ArH), 7.53–7.57 (m, 5H, ArH), 7.29 (d, *J* = 8.0 Hz, 1H, ArH), 7.24 (d, *J* = 7.5 Hz, 1H, ArH), 7.11 (s, 1H, ArH), 6.96–7.03 (m, 1H, ArH), 6.87–6.94 (m, 1H, ArH), 6.23 (br, 1H, OH), 4.04–4.08 (m, 2H, NCH_2_), 3.45–3.49 (m, 2H, CH_2_N), 2.27 (s, 3H, CH_3_), 1.95–1.99 (m, 2H, CH_2_), 1.89–1.93 (m, 2H, CH_2_); ^13^C-NMR (DMSO-*d*_6_, 125 MHz) *δ* 188.8, 160.1, 150.5, 142.8, 135.6, 133.2, 130.1, 129.7, 128.9, 128.8, 128.8, 127.4, 127.4, 125.6, 120.2, 118.9, 118.7, 115.9, 112.2, 110.7, 110.4, 105.4, 97.5, 45.5, 44.9, 29.1, 26.9, 12.9; IR (KBr) 3356, 3057, 2941, 2858, 2351, 1714, 1593, 1539, 1419, 1323, 1171, 748 cm^−1^; HRMS (ESI-TOF): *m/z* calcd for C_28_H_26_N_3_O_2_ [M + H]^+^, 436.2020; found, 436.2034.

*(4-Chlorophenyl)(9-hydroxy-7-(2-methyl-1H-indol-3-yl)-2,3,4,5-tetrahydro-1H-[1,3]diazepino[1,2-a]indol-11-yl)methan-one* (**3j**). Yellow solid, yield 87%; Mp 191–192.5 °C; ^1^H-NMR (DMSO-*d*_6_, 500 MHz) δ 10.92 (br, 1H, NH), 9.09 (br, 1H, NH), 8.37 (s, 1H, ArH), 7.56–7.60 (m, 4H, ArH), 7.28 (d, *J* = 8.0 Hz, 1H, ArH), 7.24 (d, *J* = 7.5 Hz, 1H, ArH), 7.11 (s, 1H, ArH), 6.99 (t, *J* = 7.5 Hz, 1H, ArH), 6.87–6.94 (m, 1H, ArH), 6.25 (br, 1H, OH), 4.05–4.09 (m, 2H, NCH_2_), 3.44–3.48 (m, 2H, CH_2_N), 2.27 (s, 3H, CH_3_), 1.95–1.99 (m, 2H, CH_2_), 1.88–1.92 (m, 2H, CH_2_); ^13^C-NMR (DMSO-*d*_6_, 125 MHz) δ 187.1, 160.1, 150.7, 141.4, 135.6, 134.7, 133.2, 129.7, 129.4, 129.4, 128.9, 128.9, 128.9, 125.3, 120.2, 118.9, 118.7, 115.9, 112.3, 110.6, 110.5, 105.1, 97.4, 45.5, 44.9, 29.0, 26.8, 13.0; IR (KBr) 3394, 3057, 2926, 2854, 2353, 1687, 1599, 1539, 1417, 1169, 1092, 746 cm^−1^; HRMS (ESI-TOF): *m/z* calcd for C_28_H_25_ClN_3_O_2_ [M + H]^+^, 470.1630; found, 470.1637.

*(2-Chlorophenyl)(9-hydroxy-7-(2-methyl-1H-indol-3-yl)-2,3,4,5-tetrahydro-1H-[1,3]diazepino[1,2-a]indol-11-yl)methan-one* (**3k**). Yellow solid, yield 81%; Mp 240.5–241.5 °C; ^1^H-NMR (DMSO-*d*_6_, 500 MHz) δ 10.94 (br, 1H, NH), 9.21 (br, 1H, NH), 8.15 (s, 1H, ArH), 7.61 (d, *J* = 7.5 Hz, 1H, ArH), 7.46–7.56 (m, 2H, ArH), 7.35–7.41 (m, 1H, ArH), 7.30 (d, *J* = 8.0 Hz, 1H, ArH), 7.24 (d, *J* = 7.5 Hz, 1H, ArH), 7.09 (s, 1H, ArH), 7.00 (t, *J* = 7.5, 1H, ArH), 6.91 (t, *J* = 7.5 Hz, 1H, ArH), 5.69 (br, 1H, OH), 4.01–4.08 (m, 2H, NCH_2_), 3.51–3.55 (m, 2H, CH_2_N), 2.27 (s, 3H, CH_3_), 1.98–2.02 (m, 2H, CH_2_), 1.89–1.94 (m, 2H, CH_2_); ^13^C-NMR (DMSO-*d*_6_, 125 MHz) δ 185.0, 159.8, 150.8, 142.0, 135.6, 133.3, 130.6, 130.1, 130.0, 129.5, 128.9, 128.1, 128.1, 125.4, 120.3, 118.9, 118.8, 116.0, 112.3, 110.7, 110.3, 104.6, 97.8, 45.5, 44.7, 28.8, 26.8, 13.0; IR (KBr) 3398, 3063, 2937, 2347, 1726, 1597, 1439, 1336, 1176, 750 cm^−1^; HRMS (ESI-TOF): *m/z* calcd for C_28_H_25_ClN_3_O_2_ [M + H]^+^, 470.1630; found, 470.1621.

*(4-Fluorophenyl)(9-hydroxy-7-(2-methyl-1H-indol-3-yl)-2,3,4,5-tetrahydro-1H-[1,3]diazepino[1,2-a]indol-11-yl)methan-one* (**3l**). Yellow solid, yield 83%; Mp 259–261 °C; ^1^H-NMR (DMSO-*d*_6_, 500 MHz) δ 10.91 (br, 1H, NH), 9.06 (br, 1H, NH), 8.32 (s, 1H, ArH), 7.59–7.65 (m, 2H, ArH), 7.35 (t, *J* = 8.5 Hz, 2H, ArH), 7.29 (d, *J* = 7.5 Hz, 1H, ArH), 7.24 (d, *J* = 7.5 Hz, 1H, ArH), 7.11 (s, 1H, ArH), 6.96–7.03 (m, 1H, ArH), 6.86–6.94 (m, 1H, ArH), 6.24 (br, 1H, OH), 4.04–4.08 (m, 2H, NCH_2_), 3.44–3.48 (m, 2H, CH_2_N), 2.28 (s, 3H, CH_3_), 1.95–1.99 (m, 2H, CH_2_), 1.89–1.93 (m, 2H, CH_2_); ^13^C-NMR (DMSO-*d*_6_, 125 MHz) δ 187.4, 164.2, 162.3, 160.0, 150.7, 139.2, 135.6, 133.2, 129.9, 129.7, 129.7, 128.9, 125.5, 120.2, 118.9, 118.7, 115.8, 115.6, 112.3, 110.6, 110.5, 105.1, 97.5, 45.5, 44.9, 29.1, 26.9, 13.0; IR (KBr) 3390, 3064, 2929, 2343, 1720,1595, 1535, 1428, 1222, 1167, 749 cm^−1^; HRMS (ESI-TOF): *m/z* calcd for C_28_H_25_FN_3_O_2_ [M + H]^+^, 454.1925; found, 454.1936.

*(7-Hydroxy-5-(2-methyl-1H-indol-3-yl)-2,3-dihydro-1H-imidazo[1,2-a]indol-9-yl)(4-methoxyphenyl)methanone* (**3m**). Yellow solid, yield 82%; Mp 269–271 °C; ^1^H-NMR (DMSO-*d*_6_, 500 MHz) δ 10.93 (br, 1H, NH), 8.40 (br, 1H, NH), 7.63–7.67 (m, 2H, ArH), 7.28–7.32 (m, 2H, ArH), 7.04–7.08 (m, 5H, ArH), 6.90–6.94 (m, 2H, ArH), 3.98–4.10 (m, 4H, CH_2_CH_2_), 3.85 (s, 3H, OCH_3_), 2.32 (s, 3H, CH_3_); ^13^C-NMR (DMSO-*d*_6_, 125 MHz) δ 186.5, 161.2, 159.5, 150.4, 135.6, 134.8, 133.1, 130.7, 129.7, 129.7, 128.9, 126.0, 120.2, 118.9, 118.7, 115.0, 114.0, 114.0, 111.1, 110.7, 106.8, 93.4, 55.6, 49.5, 42.5, 13.0; IR (KBr) 3390, 3059, 2966, 2843, 2353, 1726, 1597, 1473, 1325, 1248, 1163, 744 cm^−1^; HRMS (ESI-TOF): *m/z* calcd for C_27_H_24_N_3_O_3_ [M + H]^+^, 438.1812; found, 438.1786.

*(7-Hydroxy-5-(2-methyl-1H-indol-3-yl)-2,3-dihydro-1H-imidazo[1,2-a]indol-9-yl)(p-tolyl)methanone* (**3n**). Yellow solid, yield 75%; Mp 298.5–300 °C; ^1^H-NMR (DMSO-*d*_6_, 300 MHz) δ 10.91 (br, 1H, NH), 8.38 (br, 1H, NH), 7.53 (d, *J* = 6.5 Hz, 2H, ArH), 7.25–7.34 (m, 4H, ArH), 6.88–7.02 (m, 5H, ArH), 3.97–4.09 (m, 4H, CH_2_CH_2_), 2.40 (s, 3H, ArCH_3_), 2.29 (s, 3H, CH_3_); ^13^C-NMR (DMSO-*d*_6_, 125 MHz) δ 187.2, 159.7, 150.4, 140.1, 139.6, 135.6, 133.1, 130.5, 129.3, 129.3, 128.9, 127.6, 127.6, 126.0, 120.3, 118.9, 118.7, 115.2, 111.1, 110.7, 110.5, 106.9, 93.5, 49.5, 42.5, 21.5, 13.0; IR (KBr) 3408, 2899, 2584, 2345, 1726, 1597, 1475, 1327, 1167, 752 cm^−1^; HRMS (ESI-TOF): *m/z* calcd for C_27_H_24_N_3_O_2_ [M + H]^+^, 422.1863; found, 422.1837.

*(7-Hydroxy-5-(2-methyl-1H-indol-3-yl)-2,3-dihydro-1H-imidazo[1,2-a]indol-9-yl)(phenyl)methanone* (**3o**). Yellow solid, yield 73%; Mp 289.5–290.5 °C; ^1^H-NMR (DMSO-*d*_6_, 500 MHz) δ 10.92 (br, 1H, NH), 8.33 (br, 1H, NH), 7.61–7.65 (m, 2H, ArH), 7.52–7.56 (m, 3H, ArH), 7.28–7.32 (m, 2H, ArH), 6.91–7.02 (m, 5H, ArH), 3.99–4.11 (m, 4H, CH_2_CH_2_), 2.31 (s, 3H, CH_3_); ^13^C-NMR (DMSO-*d*_6_, 125 MHz) δ 187.1, 159.7, 150.4, 142.6, 135.6, 133.1, 130.8, 130.4, 130.3, 128.8, 128.8, 127.5, 127.5, 126.1, 120.2, 118.9, 118.7, 115.2, 111.1, 110.7, 110.5, 106.8, 93.5, 49.5, 42.5, 13.0; IR (KBr): 3419, 3059, 2970, 2316, 1730, 1603, 1510, 1335, 1227, 744 cm^−1^; HRMS (ESI-TOF): *m/z* calcd for C_26_H_22_N_3_O_2_ [M + H]^+^, 408.1707; found, 408.1713.

*(4-Chlorophenyl)(7-hydroxy-5-(2-methyl-1H-indol-3-yl)-2,3-dihydro-1H-imidazo[1,2-a]indol-9-yl)methanone* (**3p**). Yellow solid, yield 75%; Mp 204–206 °C; ^1^H-NMR (DMSO-*d*_6_, 500 MHz) δ 10.95 (br, 1H, NH), 8.48 (br, 1H, NH), 7.60–7.66 (m, 2H, ArH), 7.54–7.60 (m, 2H, ArH), 7.25–7.32 (m, 3H, ArH), 7.00 (t, *J* = 7.0 Hz, 1H, ArH), 6.89–6.95 (m, 3H, ArH), 4.05–4.11 (m, 2H, NCH_2_), 3.98–4.03 (m, 2H, CH_2_N), 2.30 (s, 3H, CH_3_); ^13^C-NMR (DMSO-*d*_6_, 125 MHz) δ 185.6, 159.7, 150.5, 141.2, 135.6, 134.9, 133.1, 130.3, 129.5, 129.5, 128.9, 128.9, 128.9, 126.0, 120.2, 118.9, 118.7, 115.3, 111.2, 110.7, 110.5, 106.7, 93.3, 49.5, 42.5, 13.0; IR (KBr) 3435, 3072, 2902, 2347, 1724, 1600, 1510, 1402, 1330, 1223, 752 cm^−1^; HRMS (ESI-TOF): *m/z* calcd for C_26_H_21_ClN_3_O_2_ [M + H]^+^, 442.1317; found, 442.1309.

*(2-Chlorophenyl)(7-hydroxy-5-(2-methyl-1H-indol-3-yl)-2,3-dihydro-1H-imidazo[1,2-a]indol-9-yl)methanone* (**3q**). Yellow solid, yield 72%; mp 339–341 °C; ^1^H-NMR (DMSO-*d*_6_, 500 MHz) δ 10.92 (br, 1H, NH), 7.87–8.07 (m, 1H, ArH), 7.10–7.60 (m, 8H, ArH), 6.87–7.02 (m, 3H, ArH), 4.08– 4.12(m, 4H, CH_2_CH_2_), 2.26 (s, 3H, CH_3_); ^13^C-NMR (DMSO-*d*_6_, 125 MHz) δ 184.0, 160.1, 150.1, 142.1, 135.6, 133.2, 130.6, 130.2, 129.5, 128.8, 128.1, 128.1, 128.1, 126.1, 120.3, 118.8, 118.8, 115.4, 111.2, 110.7, 110.3, 105.7, 94.1, 49.6, 42.4, 12.9; IR (KBr) 3429, 3346, 3059, 2918, 2580, 2318, 1728, 1520, 1464, 1327, 1225, 748 cm^−1^; HRMS (ESI-TOF): *m/z* calcd for C_26_H_21_ClN_3_O_2_ [M + H]^+^, 442.1317; found, 442.1302.

*(4-Fluorophenyl)(7-hydroxy-5-(2-methyl-1H-indol-3-yl)-2,3-dihydro-1H-imidazo[1,2-a]indol-9-yl)methanone* (**3r**). Yellow solid, yield 77%; Mp 268–270 °C; ^1^H-NMR (DMSO-*d*_6_, 500 MHz) δ 10.92 (br, 1H, NH), 8.39 (br, 1H, NH), 7.65–7.72 (m, 2H, ArH), 7.27–7.37 (m, 4H, ArH), 6.89–7.04 (m, 5H, ArH), 4.06–4.13 (m, 2H, NCH_2_), 3.98–4.04 (m, 2H, CH_2_N), 2.31 (s, 3H, CH_3_); ^13^C-NMR (DMSO-*d*_6_, 125 MHz) δ 185.8, 164.4, 162.4, 159.7, 150.5, 139.0, 135.6, 133.1, 130.4, 130.0, 130.0, 128.9, 126.0, 120.2, 118.9, 118.7, 115.8, 115.6, 115.2, 111.2, 110.7, 106.7, 93.3, 49.5, 42.5, 13.0; IR (KBr) 3429, 3072, 2902, 2582, 2347, 1724, 1601, 1510, 1402, 1331, 1223, 1157, 752 cm^−1^; HRMS (ESI-TOF): *m/z* calcd for C_26_H_21_FN_3_O_2_ [M + H]^+^, 426.1612; found, 426.1622.

*(6-(1,2-Dimethyl-1H-indol-3-yl)-8-hydroxy-1,2,3,4-tetrahydropyrimido[1,2-a]indol-10-yl)(4-methoxyphenyl)methanone* (**3s**). Yellow solid, yield 87%; Mp 261–263 °C; ^1^H-NMR (CDCl_3_, 400 MHz) δ 8.61 (br, 1H, NH), 7.69 (d, *J* = 8.8 Hz, 2H, ArH), 7.39 (d, *J* = 8.0 Hz, 1H, ArH), 7.34 (d, *J* = 8.0 Hz, 1H, ArH), 7.20–7.22 (m, 1H, ArH), 7.07–7.11 (m, 1H, ArH), 6.98 (d, *J* = 8.4 Hz, 2H, ArH), 6.86 (s, 1H, ArH), 6.71 (s, 1H, ArH), 4.93 (br, 1H, OH), 3.90–3.93 (m, 2H, CH_2_N), 3.86 (s, 3H, NCH_3_), 3.75 (s, 3H, OCH_3_), 3.53–3.57 (m, 2H, CH_2_), 2.34 (s, 3H, CH_3_), 2.21–2.24 (m, 2H, CH_2_N); ^13^C-NMR (CDCl_3_, 100 MHz) δ 188.8, 161.0, 153.5, 149.3, 137.0, 135.4, 134.9, 129.4, 129.4, 129.3, 127.5, 126.6, 121.5, 119.9, 118.9, 113.6, 113.6, 113.2, 109.5, 108.9, 107.7, 104.7, 95.8, 55.3, 39.3, 38.1, 29.9, 20.8, 11.1; IR (KBr) 3439, 2926, 2853, 2347, 1728, 1616, 1510, 1471, 1350, 1324, 1168, 740 cm^−1^; HRMS (ESI-TOF): *m/z* calcd for C_29_H_28_N_3_O_3_ [M + H]^+^, 466.2125; found, 466.2120.

*(6-(1,2-Dimethyl-1H-indol-3-yl)-8-hydroxy-1,2,3,4-tetrahydropyrimido[1,2-a]indol-10-yl)(4-methoxyphenyl)methanone(6-(1,2-dimethyl-1H-indol-3-yl)-8-hydroxy-1,2,3,4-tetrahydropyrimido[1,2-a]indol-10-yl)(4-fluorophenyl)methanone* (**3t**). Yellow solid, yield 70%; Mp 173–175 °C; ^1^H-NMR (CDCl_3_, 400 MHz) δ 8.62 (br, 1H, NH), 7.67–7.71 (m, 2H, ArH), 7.34–7.39 (m, 2H, ArH), 7.11–7.26 (m, 3H, ArH), 7.08–7.11 (m, 1H, ArH), 6.87 (s, 1H, ArH), 6.53 (s, 1H, ArH), 4.88 (br, 1H, OH), 3.94 (t, *J* = 6.0 Hz, 2H, CH_2_N), 3.77 (s, 3H, NCH_3_), 3.57–3.63 (m, 2H, CH_2_), 2.35 (s, 3H, CH_3_), 2.23–2.29 (m, 2H, CH_2_N); ^13^C-NMR (CDCl_3_, 100 MHz) δ 188.0, 162.5, 153.6, 149.4, 138.5, 137.0, 135.4, 129.6, 129.5, 129.4, 127.4, 126.4, 121.6, 120.0, 118.8, 115.5, 115.3, 113.4, 109.6, 108.9, 107.6, 104.5, 95.9, 39.3, 38.1, 29.9, 20.7, 11.1; IR (KBr) 3437, 2925, 2582, 1721, 1617, 1534, 1470, 1325, 1221, 1173, 775 cm^−1^; HRMS (ESI-TOF): *m/z* calcd for C_28_H_25_N_3_O_2_ [M + H]^+^, 454.1925; found, 454.1931.

*(8-Hydroxy-6-(2-phenyl-1H-indol-3-yl)-1,2,3,4-tetrahydropyrimido[1,2-a]indol-10-yl)(4-methoxyphenyl)methanone* (**3u**). Yellow solid, yield 85%; Mp 179–181 °C; ^1^H-NMR (CDCl_3_, 400 MHz) δ 8.92 (br, 1H, NH), 8.59 (br, 1H, NH), 7.69 (d, *J* = 8.8 Hz, 2H, ArH), 7.45–7.40 (m, 4H, ArH), 7.28–7.22 (m, 4H, ArH), 7.13–7.10 (m, 1H, ArH), 6.96 (d, *J* = 8.8 Hz, 2H, ArH), 6.85 (s, 1H, ArH), 6.72 (s, 1H, ArH), 4.94 (br, 1H, OH), 3.82–3.86 (m, 5H, CH_2_N, OCH_3_), 3.47–3.51 (m, 2H, NCH_2_), 2.19–2.16 (m, 2H, CH_2_); ^13^C-NMR (CDCl_3_, 100 MHz) δ 188.9, 161.1, 153.6, 149.3, 136.2, 135.2, 134.8, 131.9, 129.7, 129.5, 129.4, 129.4, 128.9, 128.9, 127.9, 127.0, 127.0, 123.1, 120.6, 119.8, 113.6, 113.6, 113.1, 113.0, 111.1, 109.5, 108.8, 105.2, 95.9, 55.3, 39.2, 38.0, 20.7; IR (KBr) 3438, 2925, 2854, 1728, 1616, 1577, 1532, 1445, 1326, 1253, 1168, 747 cm^−1^; HRMS (ESI-TOF): *m/z* calcd for C_33_H_28_N_3_O_3_ [M + H]^+^, 514.2125; found, 514.2121.

*(4-Fluorophenyl)(8-hydroxy-6-(2-phenyl-1H-indol-3-yl)-1,2,3,4-tetrahydropyrimido[1,2-a]indol-10-yl)methanone* (**3v**). Yellow solid; yield 75%; Mp 264–266 °C; ^1^H-NMR (CDCl_3_, 400 MHz): δ 8.75 (br, 1H, NH), 8.61 (br, 1H, NH), 7.71–7.67 (m, 2H, ArH), 7.44–7.40 (m, 4H, ArH), 7.31–7.26 (m, 4H, ArH), 7.16–7.11 (m, 3H, ArH), 6.85 (s, 1H, ArH), 6.54 (s, 1H, ArH), 4.92 (br, 1H, OH), 3.86–3.83 (m, 2H, CH_2_N), 3.52–3.56 (m, 2H, NCH_2_), 2.22–2.19 (m, 2H, CH_2_); ^13^C-NMR (CDCl_3_, 100 MHz): δ 187.9, 165.0, 162.5, 153.7, 149.4, 138.4, 138.4, 136.1, 135.2, 131.9, 129.7, 129.6, 129.5, 128.9, 128.0, 126.9, 126.7, 123.2, 120.7, 119.8, 115.5, 115.3, 113.2, 111.1, 109.7, 108.7, 105.0, 104.9, 95.9, 39.2, 38.0, 20.4; IR (KBr) 3426, 2924, 1721, 1617, 1535, 1478, 1325, 1221, 1176, 774 cm^−1^; HRMS (ESI-TOF): *m/z* calcd for C_32_H_25_FN_3_O_2_ [M + H]^+^, 502.1925; found, 502.1933.

*(8-Hydroxy-6-(5-methoxy-1H-indol-3-yl)-1,2,3,4-tetrahydropyrimido[1,2-a]indol-10-yl)(4-methoxyphenyl)methanone* (**3w**). Yellow solid; yield 65%; Mp 184–186 °C; ^1^H-NMR (CDCl_3_, 400 MHz) δ 8.60 (br, 1H, NH), 8.42 (br, 1H, NH), 7.70–7.68 (m, 1H, ArH), 7.64–7.61 (m, 2H, ArH), 7.35–7.33 (m, 1H, ArH), 7.01–6.94 (m, 5H, ArH), 6.70 (s, 1H, ArH), 5.08 (br, 1H, OH), 3.98–3.94 (m, 2H, CH_2_N), 3.88 (s, 3H, OCH_3_), 3.80 (s, 3H, OCH_3_), 3.58–3.56 (m, 2H, NCH_2_), 2.27–2.23 (m, 2H, CH_2_); ^13^C-NMR (CDCl_3_, 100 MHz) δ 188.8, 161.0, 154.8, 153.6, 148.9, 137.8, 134.7, 131.5, 129.4, 129.4, 126.5, 123.9, 118.8, 116.3, 113.6, 113.5, 112.3, 108.7, 107.6, 105.3, 105.1, 101.1, 95.8, 55.9, 55.3, 39.2, 38.0, 20.7; IR (KBr) 3430, 2921, 2852, 1724, 1612, 1557, 1528, 1450, 1340, 1250, 1170, 750 cm^−1^; HRMS (ESI-TOF): *m/z* calcd for C_28_H_26_N_3_O_4_ [M + H]^+^, 468.1918; found, 468.1922.

## 4. Conclusions

In summary, we have successfully developed a facile, economical, and environmentally friendly method for the construction of highly functionalized 3,7′-bisindole derivatives via a Michael addition/cyclocondensation reaction. This allowed for the rapid construction of a novel library of highly substituted 3,7′-bisindole derivatives through the simple and easy raw material HKAs **1** and 2-(1*H*-indol-3-yl)cyclohexa-2,5-diene-1,4-dione derivatives **2**. Our further investigations into the *in vitro* biological activities of compounds **3** are currently ongoing.

## Figures and Tables

**Table 1:** Optimization of reaction conditions ^a^. 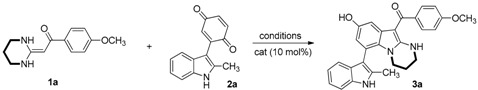

Entry	Solvent	Catalyst	*t* (°C)	Time (h)	Yield (%) ^b^
1	EtOH	−	rt	12	trace
2	EtOH	HOAc	rt	12	45
3	EtOH	Et_3_N	rt	12	91
4	EtOH	Na_2_CO_3_	rt	12	67
5	EtOH	K_2_CO_3_	rt	12	66
6	EtOH	EtONa	rt	12	25
7	EtOH	DBU	rt	12	trace
8	CH_2_Cl_2_	Et_3_N	rt	12	78
9	MeCN	Et_3_N	rt	12	81
10	tetrahydrofuran	Et_3_N	rt	12	75
11	toluene	Et_3_N	rt	12	68
12	MeOH	Et_3_N	rt	12	72
13	H_2_O	Et_3_N	rt	12	30
14	EtOH	Et_3_N	40	18	67
15	EtOH	Et_3_N	reflux	24	45

^a^ The reaction was performed with **1a** (0.1 mmol), **2a** (0.11 mmol). ^b^ Isolated yields based on HKA **1a**.

**Table 2:** Preparation of the of 3,7′-bisindole derivatives ^a^. 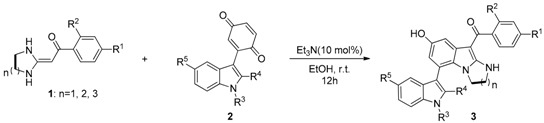

Entry	n	R^1^	R^2^	R^3^	R^4^	R^5^	3	Yield (%) ^b^
1	2	MeO	H	H	Me	H	**3a**	91
2	2	Me	H	H	Me	H	**3b**	88
3	2	H	H	H	Me	H	**3c**	82
4	2	Cl	H	H	Me	H	**3d**	89
5	2	H	Cl	H	Me	H	**3e**	86
6	2	F	H	H	Me	H	**3f**	89
7	3	MeO	H	H	Me	H	**3g**	87
8	3	Me	H	H	Me	H	**3h**	84
9	3	H	H	H	Me	H	**3i**	83
10	3	Cl	H	H	Me	H	**3j**	87
11	3	H	Cl	H	Me	H	**3k**	81
12	3	F	H	H	Me	H	**3l**	83
13	1	MeO	H	H	Me	H	**3m**	82
14	1	Me	H	H	Me	H	**3n**	75
15	1	H	H	H	Me	H	**3o**	73
16	1	Cl	H	H	Me	H	**3p**	75
17	1	H	Cl	H	Me	H	**3q**	72
18	1	F	H	H	Me	H	**3r**	77
19	2	MeO	H	Me	Me	H	**3s**	87
20	2	F	H	Me	Me	H	**3t**	70
21	2	MeO	H	H	Ph	H	**3u**	85
22	2	F	H	H	Ph	H	**3v**	75
23	2	MeO	H	H	H	MeO	**3w**	65

^a^ The reaction was performed with **1** (0.1 mmol), **2** (0.11 mmol). ^b^ Isolated yields based on HKA **1**.

## Supplementary Materials

Supplementary materials can be accessed at: http://www.mdpi.com/1420-3049/21/5/638/s1.
