# Supplementary material for: A Convenient Synthesis of 3,7′-Bisindole Derivatives
Source: Molecules. 2016 May 17;21(5):638. doi: 10.3390/molecules21050638 (PMC6273136; doi:10.3390/molecules21050638)
Supplement: Supplementary file 1 [file molecules-21-00638-s001.pdf]

Supplementary Materials: A Convenient Synthesis of 3,7′-Bisindole Derivatives

Teng Liu, Hong-You Zhu, Da-Yun Luo, Sheng-Jiao Yan and Jun Lin

Table of Contents

General Information S2
General Procedure for the Preparation of **3** S2
Spectroscopic Data of **3** S2–S10
Figure S1. ^{1}H-NMR (500 MHz, DMSO-*d*$_6$) spectra of compound **3a** S11
Figure S2. ^{13}C-NMR (125 MHz, DMSO-*d*$_6$) spectra of compound **3a** S12
Figure S3. ^{1}H-NMR (500 MHz, DMSO-*d*$_6$) spectra of compound **3b** S13
Figure S4. ^{13}C-NMR (125 MHz, DMSO-*d*$_6$) spectra of compound **3b** S14
Figure S5. ^{1}H-NMR (500 MHz, DMSO-*d*$_6$) spectra of compound **3c** S15
Figure S6. ^{13}C-NMR (125 MHz, DMSO-*d*$_6$) spectra of compound **3c** S16
Figure S7. ^{1}H-NMR (500 MHz, DMSO-*d*$_6$) spectra of compound **3d** S17
Figure S8. ^{13}C-NMR (125 MHz, DMSO-*d*$_6$) spectra of compound **3d** S18
Figure S9. ^{1}H-NMR (500 MHz, DMSO-*d*$_6$) spectra of compound **3e** S19
Figure S10. ^{13}C-NMR (125 MHz, DMSO-*d*$_6$) spectra of compound **3e** S20
Figure S11. ^{1}H-NMR (500 MHz, DMSO-*d*$_6$) spectra of compound **3f** S21
Figure S12. ^{13}C-NMR (125 MHz, DMSO-*d*$_6$) spectra of compound **3f** S22
Figure S13. ^{1}H-NMR (500 MHz, DMSO-*d*$_6$) spectra of compound **3g** S23
Figure S14. ^{13}C-NMR (125 MHz, DMSO-*d*$_6$) spectra of compound **3g** S24
Figure S15. ^{1}H-NMR (500 MHz, DMSO-*d*$_6$) spectra of compound **3h** S25
Figure S16. ^{13}C-NMR (125 MHz, DMSO-*d*$_6$) spectra of compound **3h** S26
Figure S17. ^{1}H-NMR (500 MHz, DMSO-*d*$_6$) spectra of compound **3i** S27
Figure S18. ^{13}C-NMR (125 MHz, DMSO-*d*$_6$) spectra of compound **3i** S28
Figure S19. ^{1}H-NMR (500 MHz, DMSO-*d*$_6$) spectra of compound **3j** S29
Figure S20. ^{13}C-NMR (125 MHz, DMSO-*d*$_6$) spectra of compound **3j** S30
Figure S21. ^{1}H-NMR (500 MHz, DMSO-*d*$_6$) spectra of compound **3k** S31
Figure S22. ^{13}C-NMR (125 MHz, DMSO-*d*$_6$) spectra of compound **3k** S32
Figure S23. ^{1}H-NMR (500 MHz, DMSO-*d*$_6$) spectra of compound **3l** S33
Figure S24. ^{13}C-NMR (125 MHz, DMSO-*d*$_6$) spectra of compound **3l** S34
Figure S25. ^{1}H-NMR (500 MHz, DMSO-*d*$_6$) spectra of compound **3m** S35
Figure S26. ^{13}C-NMR (125 MHz, DMSO-*d*$_6$) spectra of compound **3m** S36
Figure S27. ^{1}H-NMR (500 MHz, DMSO-*d*$_6$) spectra of compound **3n** S37
Figure S28. ^{13}C-NMR (125 MHz, DMSO-*d*$_6$) spectra of compound **3n** S38
Figure S29. ^{1}H-NMR (500 MHz, DMSO-*d*$_6$) spectra of compound **3o** S39
Figure S30. ^{13}C-NMR (125 MHz, DMSO-*d*$_6$) spectra of compound **3o** S40
Figure S31. ^{1}H-NMR (500 MHz, DMSO-*d*$_6$) spectra of compound **3p** S41
Figure S32. ^{13}C-NMR (125 MHz, DMSO-*d*$_6$) spectra of compound **3p** S42
Figure S33. ^{1}H-NMR (500 MHz, DMSO-*d*$_6$) spectra of compound **3q** S43
Figure S34. ^{13}C-NMR (125 MHz, DMSO-*d*$_6$) spectra of compound **3q** S44
Figure S35. ^{1}H-NMR (500 MHz, DMSO-*d*$_6$) spectra of compound **3r** S45
Figure S36. ^{13}C-NMR (125 MHz, DMSO-*d*$_6$) spectra of compound **3r** S46
Figure S37. ^{1}H-NMR (400 MHz, $CDCl_3$) spectra of compound **3s** S47
Figure S38. ^{13}C-NMR (100 MHz, $CDCl_3$) spectra of compound **3s** S40
Figure S39. ^{1}H-NMR (400 MHz, $CDCl_3$) spectra of compound **3t** S41
Figure S40. ^{13}C-NMR (100 MHz, $CDCl_3$) spectra of compound **3t** S50
Figure S41. ^{1}H-NMR (400 MHz, $CDCl_3$) spectra of compound **3u** S51
Figure S42. ^{13}C-NMR (100 MHz, $CDCl_3$) spectra of compound **3u** S52
Figure S43. ^{1}H-NMR (400 MHz, $CDCl_3$) spectra of compound **3v** S53
Figure S44. ^{13}C-NMR (100 MHz, $CDCl_3$) spectra of compound **3v** S54

Figure S45. ^{1}H-NMR (400 MHz, $CDCl_3$) spectra of compound **3w** S55
Figure S46. ^{13}C-NMR (100 MHz, $CDCl_3$) spectra of compound **3w** S56
References and Notes S57

General Information

All compounds were fully characterised by spectroscopic data. The NMR spectra were recorded on a Bruker DRX400 & DRX500. Chemical shifts (δ) are expressed in ppm, *J* values are given in Hz, and deuterated DMSO-d_6 and $CDCl_3$ were used as solvent. IR spectra were recorded on a FT-IR Thermo Nicolet Avatar 360 using a KBr pellet. The reactions were monitored by thin layer chromatography (TLC) using silica gel GF_{254}. The melting points were determined on a XT-4A melting point apparatus and are uncorrected. HRMs were performed on an Agilent LC/Msd TOF instrument.

The materials were purchased from Adamas-beta Corporation Limited. All chemicals and solvents were used as received without further purification unless otherwise stated. Column chromatography was performed on silica gel (200–300 mesh).

Compounds **1** were prepared according to the literature [1-3]. Compound **2** were prepared according to the literature [4-5].

General Procedure for the Preparation of 3

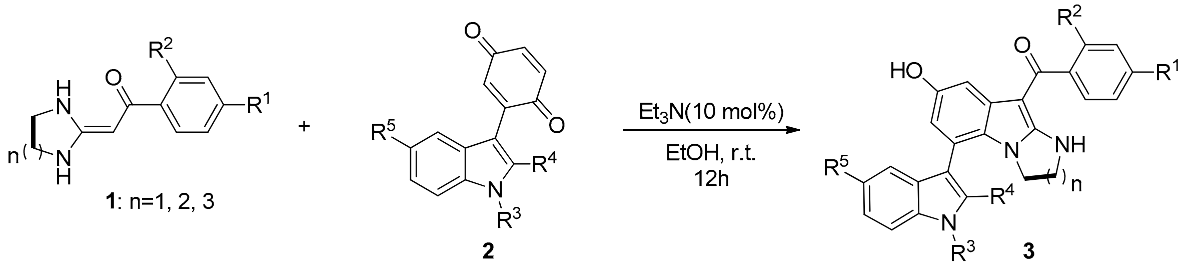

To a 10 ml round-bottom flask, HKAs **1** (0.1 mmol) and substrate **2** (0.11 mmol) were added to a solution of Anhydrous ethanol (1 mL) and Et_3N (10 mol%), stirred at room temperature. The mixture was stirred for 12 h until the **1** were completely consumed. The solvent and other volatile liquids were evaporated under vacuum. The raw materials were purified by column chromatography to afford the 3,7′-bisindole derivatives **3** in 65%–91%.

Spectroscopic Data of 3

(8-Hydroxy-6-(2-methyl-1*H*-indol-3-yl)-1,2,3,4-tetrahydropyrimido[1,2-*a*]indol-10-yl)(4-methoxyphenyl)methanone (**3a**)

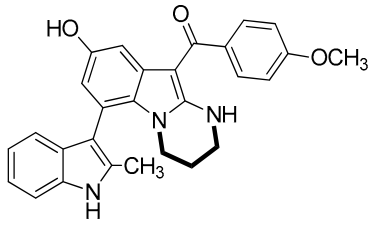

Yellow solid; Mp 221.5–222.5 °C; IR (KBr): 3439, 3230, 2904, 2586, 1722, 1599, 1514, 1333, 1223, 752 cm^{-1}; ^{1}H-NMR (500 MHz, DMSO-d_6): δ = 10.92 (br, 1H, NH), 8.51 (br, 1H, NH), 8.23 (s, 1H, ArH), 7.58 (d, *J* = 8.0 Hz, 2H, ArH), 7.28–7.30 (m, 2H, ArH), 7.06 (d, *J* = 8.0 Hz, 2H, ArH), 6.97–7.03 (m, 1H, ArH), 6.91–6.94 (m, 2H, ArH), 6.50 (br, 1H, OH), 3.91–3.95 (m, 2H, NCH_2), 3.85 (s, 3H, OCH_3), 3.46–3.50 (m, 2H, CH_2N), 2.30 (s, 3H, CH_3), 2.07–2.11 (m, 2H, CH_2); ^{13}C-NMR (125 MHz, DMSO-d_6): δ = 186.8, 160.7, 153.1, 150.3, 135.6, 135.5, 133.1, 129.4, 129.4, 128.9, 128.8, 125.3, 120.2, 118.9, 118.7, 114.7, 113.9, 113.9, 113.9, 110.6, 110.5, 105.4, 94.9, 55.6, 39.3, 38.1, 20.6, 13.0; HRMS (ESI-TOF): m/z calcd. for $C_{28}H_{26}N_3O_3$ [M + H]$^+$, 452.1969; found, 452.1947.

(8-Hydroxy-6-(2-methyl-1*H*-indol-3-yl)-1,2,3,4-tetrahydropyrimido[1,2-*a*]indol-10-yl)(*p*-tolyl)methanone (**3b**)

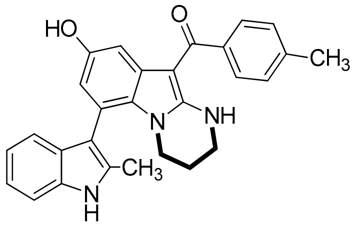

Yellow solid; Mp 228–230 °C; IR (KBr): 3394, 3053, 2928, 2316, 1728, 1591, 1443, 1335, 1171, 750 cm^{-1}; ^{1}H-NMR (500 MHz, DMSO-d_6): δ = 10.82 (br, 1H, NH), 8.44 (br, 1H, NH), 8.19 (s, 1H, ArH), 7.41 (d, *J* = 7.5 Hz, 2H, ArH), 7.23–7.32 (m, 3H, ArH), 7.21 (d, *J* = 7.5 Hz, 1H, ArH), 6.98 (t, *J* = 7.5 Hz, 1H, ArH), 6.85–6.92 (m, 2H, ArH), 6.32 (br, 1H, OH), 3.82–3.89 (m, 2H, NCH_2), 3.42–3.46 (m, 2H, CH_2N), 2.37 (s, 3H, CH_3), 2.24 (s, 3H, $ArCH_3$), 2.04–2.08 (m, 2H, CH_2); ^{13}C-NMR (125 MHz, DMSO-d_6): δ = 187.6, 153.3, 150.1, 139.8, 139.7, 135.5, 133.3, 129.3, 129.3, 129.0, 128.7, 127.3, 127.3, 125.1, 120.4, 118.9, 118.7, 115.1, 110.8, 110.5, 110.2, 105.4, 95.2, 39.5, 38.0, 21.4, 20.3, 12.7; HRMS (ESI-TOF): m/z calcd. for $C_{28}H_{26}N_3O_2$ [M + H]$^+$, 436.2020; found, 436.2005.

(8-Hydroxy-6-(2-methyl-1*H*-indol-3-yl)-1,2,3,4-tetrahydropyrimido[1,2-*a*]indol-10-yl) -(phenyl)methanone (**3c**)

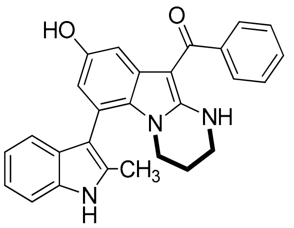

Yellow solid; Mp 317–318.5 °C; IR (KBr): 3323, 3055, 2972, 2866, 2314, 1726, 1614, 1529, 1319, 1174, 746 cm^{-1}; ^{1}H-NMR (500 MHz, DMSO-d_6): δ = 10.95 (br, 1H, NH), 8.60 (br, 1H, NH), 8.19 (s, 1H, ArH), 7.54–7.58 (m, 5H, ArH), 7.30 (t, *J* = 7.5 Hz, 2H, ArH), 6.98–7.04 (m, 1H, ArH), 6.90–6.98 (m, 2H, ArH), 6.34 (br, 1H, OH), 3.92–3.96 (m, 2H, NCH_2), 3.47–3.51 (m, 2H, CH_2N), 2.30 (s, 3H, CH_3), 2.08–2.12 (m, 2H, CH_2); ^{13}C-NMR (125 MHz, DMSO-d_6): δ = 187.2, 153.2, 150.3, 143.2, 135.7, 133.2, 129.8, 128.9, 128.7, 128.7, 128.7, 127.3, 127.3, 125.2, 120.2, 118.9, 118.7, 114.9, 110.7, 110.6, 110.5, 105.4, 95.0, 39.3, 38.1, 20.6, 13.0; HRMS (ESI-TOF): m/z calcd. for $C_{27}H_{24}N_3O_2$ [M + H]$^+$, 422.1863; found, 422.1871.

(4-Chlorophenyl)(8-hydroxy-6-(2-methyl-1*H*-indol-3-yl)-1,2,3,4-tetrahydropyrimido-[1,2-*a*]indol-10-yl)methanone (**3d**)

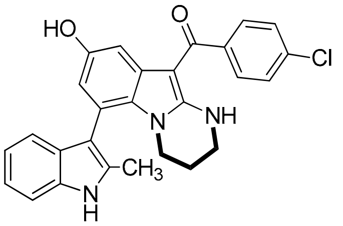

Yellow solid; Mp 199.0–201.5 °C; IR (KBr): 3400, 3063, 2951, 2866, 2349, 1680, 1616, 1527, 1331, 750 cm^{-1}; ^{1}H-NMR (500 MHz, DMSO-d_6): δ = 10.91 (br, 1H, NH), 8.55 (br, 1H, NH), 8.27–8.32 (m, 1H, ArH), 7.55–7.59 (m, 4H, ArH), 7.23–7.31 (m, 2H, ArH), 6.96–7.02 (m, 1H, ArH), 6.87–6.96 (m, 2H, ArH), 6.32 (br, 1H, OH), 3.91–3.95 (m, 2H, NCH_2), 3.42–3.46 (m, 2H, CH_2N), 2.27 (s, 3H, CH_3), 2.08–2.12 (m, 2H, CH_2); ^{13}C-NMR (125 MHz, DMSO-d_6): δ = 185.6, 153.2, 150.5, 141.8, 135.6, 134.3, 133.1, 129.3, 129.3, 129.3, 128.9, 128.9, 128.9, 128.9, 124.9, 120.2, 118.9, 118.7, 115.0, 110.7, 110.5, 105.1, 95.0, 39.4, 38.1, 20.5, 13.0; HRMS (ESI-TOF): *m*/*z* calcd. for $C_{27}H_{23}ClN_3O_2$ [M + H]$^+$, 456.1473; found, 456.1459.

(2-Chlorophenyl)(8-hydroxy-6-(2-methyl-1*H*-indol-3-yl)-1,2,3,4-tetrahydropyrimido-[1,2-*a*]indol-10-yl)methanone (**3e**)

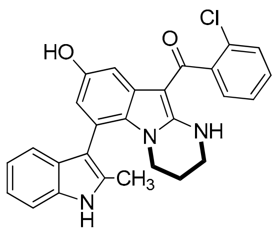

Yellow solid; Mp 301–303 °C; IR KBr): 3342, 3061, 2966, 2868, 1726, 1618, 1531, 1429, 1329, 1176, 748 cm^{-1}; ^{1}H-NMR (500 MHz, DMSO-*d*$_6$): *δ* = 10.94 (br, 1H, NH), 8.52 (br, 1H, NH), 8.07 (s, 1H, ArH), 7.61 (d, *J* = 1.0 Hz, 1H, ArH), 7.47–7.61 (m, 2H, ArH), 7.34–7.39 (m, 1H, ArH), 7.30 (d, *J* = 8.0 Hz, 1H, ArH), 7.26 (d, *J* = 7.5 Hz, 1H, ArH), 6.96–7.03 (m, 1H, ArH), 6.88–6.95 (m, 2H, ArH), 5.71 (br, 1H, OH), 3.90–3.94 (m, 2H, NCH$_2$), 3.48–3.52 (m, 2H, CH$_2$N), 2.26 (s, 3H, CH$_3$), 2.08–2.12 (m, 2H, CH$_2$); ^{13}C-NMR (125 MHz, DMSO-*d*$_6$): *δ* = 183.7, 152.8, 150.5, 142.3, 135.7, 133.2, 130.4, 130.1, 129.6, 129.0, 128.9, 128.2, 128.1, 125.0, 120.3, 118.8, 118.7, 115.1, 110.7, 110.7, 110.4, 104.7, 95.7, 39.3, 38.1, 20.4, 13.0; HRMS (ESI-TOF): *m*/*z* calcd. for $C_{27}H_{23}ClN_3O_2$ [M + H]$^+$, 456.1473; found, 456.1462.

(4-Fluorophenyl)(8-hydroxy-6-(2-methyl-1*H*-indol-3-yl)-1,2,3,4-tetrahydropyrimido-[1,2-*a*]indol-10-yl)methanone (**3f**)

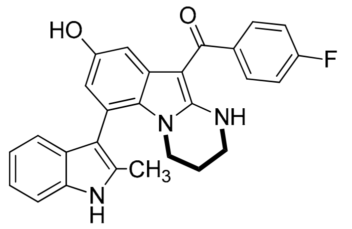

Yellow solid; Mp 247–248.5 °C; IR (KBr): 3394, 3053, 2928, 2860, 2316, 1720, 1591, 1441, 1335, 1170, 750 cm^{-1}; ^{1}H-NMR (500 MHz, DMSO-*d*$_6$): *δ* = 10.93 (br, 1H, NH), 8.54 (br, 1H, NH), 8.27 (s, 1H, ArH), 7.58–7.65 (m, 2H, ArH), 7.34 (t, *J* = 9.0 Hz, 2H, ArH), 7.28 (t, *J* = 9.0 Hz, 2H, ArH), 7.00 (t, *J* = 7.5 Hz, 1H, ArH), 6.87–6.97 (m, 2H, ArH), 6.31 (br, 1H, OH), 3.90–3.97 (m, 2H, NCH$_2$), 3.47–3.51 (m, 2H, CH$_2$N), 2.28 (s, 3H, CH$_3$), 2.08–2.12 (m, 2H, CH$_2$); ^{13}C-NMR (125 MHz, DMSO-*d*$_6$): *δ* = 185.9, 164.0, 162.1, 153.1, 150.4, 139.6, 135.6, 133.1, 129.7, 129.7, 128.9, 125.1, 120.2, 118.9, 118.7, 115.7, 115.5, 114.9, 110.7, 110.7, 110.5, 105.1, 95.0, 39.4, 38.1, 20.5, 13.0; HRMS (ESI-TOF): *m*/*z* calcd. for $C_{27}H_{23}FN_3O_2$ [M + H]$^+$, 440.1769; found, 440.1775.

(9-Hydroxy-7-(2-methyl-1*H*-indol-3-yl)-2,3,4,5-tetrahydro-1*H*-[1,3]diazepino[1,2-*a*]-indol-11-yl)(4-methoxyphenyl)methanone (**3g**)

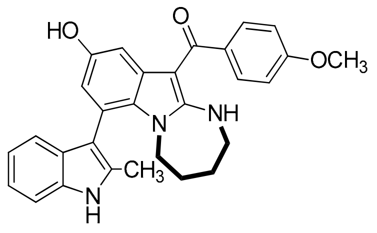

Yellow solid; Mp 179.5–182 °C; IR (KBr): 3396, 3063, 2928, 2850, 2351, 1726, 1593, 1444, 1313, 1250, 1167, 1022, 744 cm^{-1}; ^{1}H-NMR (500 MHz, DMSO-*d*$_6$): *δ* = 10.93 (br, 1H, NH), 8.96 (br, 1H, NH), 8.32 (s, 1H, ArH), 7.59 (d, *J* = 7.6 Hz, 2H, ArH), 7.29 (d, *J* = 7.5 Hz, 1H, ArH), 7.26 (d, *J* = 7.4 Hz, 1H, ArH), 7.12 (s, 1H, ArH), 7.07 (d, *J* = 7.7 Hz, 2H, ArH), 7.00 (m, 1H, ArH), 6.91 (m, 1H, ArH), 6.45 (br, 1H, OH), 4.03–4.07 (m, 2H, NCH$_2$), 3.86 (s, 3H, OCH$_3$), 3.41–3.45 (m, 2H, CH$_2$N), 2.29 (s, 3H, CH$_3$), 1.87–1.97 (m, 4H, CH$_2$CH$_2$); ^{13}C-NMR (125 MHz, DMSO-*d*$_6$): *δ* = 188.3, 161.0, 160.0, 150.5, 135.6, 135.0, 133.2, 129.7, 129.7, 129.5, 128.9, 125.7, 120.2, 118.9, 118.7, 115.7, 113.9, 113.9, 112.2, 110.6, 105.3, 97.7, 55.6, 45.5, 45.1, 29.3, 27.0, 13.0; HRMS (ESI-TOF): *m*/*z* calcd. for $C_{29}H_{28}N_3O_3$ [M + H]$^+$, 466.2125; found, 466.2145.

(9-Hydroxy-7-(2-methyl-1*H*-indol-3-yl)-2,3,4,5-tetrahydro-1*H*-[1,3]diazepino[1,2-*a*]-indol-11-yl)(*p*-tolyl)methanone (**3h**)

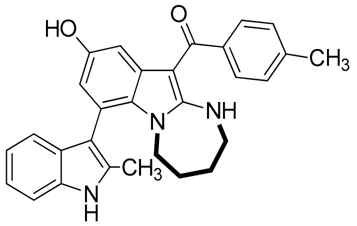

Yellow solid; Mp 242–244 °C; IR (KBr): 3394, 3053, 2926, 2858, 2314, 1726, 1593, 1446, 1335, 1169, 748 cm^{-1}; ^{1}H-NMR (500 MHz, DMSO-d_6): δ = 10.92 (br, 1H, NH), 9.05 (br, 1H, NH), 8.23 (s, 1H, ArH), 7.49 (d, J = 7.7 Hz, 2H, ArH), 7.33 (d, J = 7.6 Hz, 2H, ArH), 7.29 (d, J = 7.9 Hz, 1H, ArH), 7.25 (d, J = 7.8 Hz, 1H, ArH), 7.11 (s, 1H, ArH), 6.96–7.03 (m, 1H, ArH), 6.91 (t, J = 7.3 Hz, 1H, ArH), 6.34 (br, 1H, OH), 4.04–4.11 (m, 2H, NCH_2), 3.39–3.43 (m, 2H, CH_2N),2.43 (s, 3H, $ArCH_3$), 2.28 (s, 3H, CH_3), 1.88–1.97 (m, 4H, CH_2CH_2); ^{13}C-NMR (125 MHz, DMSO-d_6): δ = 188.8, 160.0, 150.5, 139.9, 139.8, 135.6, 133.2, 129.7, 129.2, 129.2, 128.9, 127.7, 127.7, 125.6, 120.2, 118.9, 118.7, 115.8, 112.2, 110.6, 110.5, 105.4, 97.6, 45.5, 45.0, 29.2, 26.9, 21.5, 13.0; HRMS (ESI-TOF): m/z calcd. for $C_{29}H_{28}N_3O_2$ [M + H]$^+$, 450.2176; found, 450.2184.

(9-Hydroxy-7-(2-methyl-1*H*-indol-3-yl)-2,3,4,5-tetrahydro-1*H*-[1,3]diazepino[1,2-*a*]-indol-11-yl)(phenyl)methanone (**3i**)

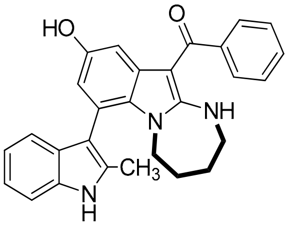

Yellow solid; Mp 289–290 °C; IR (KBr): 3356, 3057, 2941, 2858, 2351, 1714, 1593, 1539, 1419, 1323, 1171, 748 cm^{-1}; ^{1}H-NMR (500 MHz, DMSO-d_6): δ = 10.92 (br, 1H, NH), 9.10 (br, 1H, NH), 8.21 (s, 1H, ArH), 7.53–7.57 (m, 5H, ArH), 7.29 (d, J = 8.0 Hz, 1H, ArH), 7.24 (d, J = 7.5 Hz, 1H, ArH), 7.11 (s, 1H, ArH), 6.96–7.03 (m, 1H, ArH), 6.87–6.94 (m, 1H, ArH), 6.23 (br, 1H, OH), 4.04–4.08 (m, 2H, NCH_2), 3.45–3.49 (m, 2H, CH_2N), 2.27 (s, 3H, CH_3), 1.95–1.99 (m, 2H, CH_2), 1.89–1.93 (m, 2H, CH_2); ^{13}C-NMR (125 MHz, DMSO-d_6): δ = 188.8, 160.1, 150.5, 142.8, 135.6, 133.2, 130.1, 129.7, 128.9, 128.8, 128.8, 127.4, 127.4, 125.6, 120.2, 118.9, 118.7, 115.9, 112.2, 110.7, 110.4, 105.4, 97.5, 45.5, 44.9, 29.1, 26.9, 12.9; HRMS (ESI-TOF): m/z calcd. for $C_{28}H_{26}N_3O_2$ [M + H]$^+$, 436.2020; found, 436.2034.

(4-Chlorophenyl)(9-hydroxy-7-(2-methyl-1*H*-indol-3-yl)-2,3,4,5-tetrahydro-1*H*-[1,3]-diazepino[1,2-*a*]indol-11-yl)methanone (**3j**)

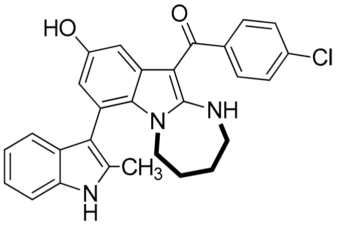

Yellow solid; Mp 191–192.5 °C; IR (KBr): 3394, 3057, 2926, 2854, 2353, 1687, 1599, 1539, 1417, 1169, 1092, 746 cm^{-1}; ^{1}H-NMR (500 MHz, DMSO-d_6): δ = 10.92 (br, 1H, NH), 9.09 (br, 1H, NH), 8.37 (s, 1H, ArH), 7.56–7.60 (m, 4H, ArH), 7.28 (d, J = 8.0 Hz, 1H, ArH), 7.24 (d, J = 7.5 Hz, 1H, ArH), 7.11 (s, 1H, ArH), 6.99 (t, J = 7.5 Hz, 1H, ArH), 6.87–6.94 (m, 1H, ArH), 6.25 (br, 1H, OH), 4.05–4.09 (m, 2H, NCH_2), 3.44–3.48 (m, 2H, CH_2N), 2.27 (s, 3H, CH_3), 1.95–1.99 (m, 2H, CH_2), 1.88–1.92 (m, 2H, CH_2); ^{13}C-NMR (125 MHz, DMSO-d_6): δ = 187.1, 160.1, 150.7, 141.4, 135.6, 134.7, 133.2, 129.7, 129.4, 129.4, 128.9, 128.9, 128.9, 125.3, 120.2, 118.9, 118.7, 115.9, 112.3, 110.6, 110.5, 105.1, 97.4, 45.5, 44.9, 29.0, 26.8, 13.0; HRMS (ESI-TOF): m/z calcd. for $C_{28}H_{25}ClN_3O_2$ [M + H]$^+$, 470.1630; found, 470.1637.

(2-Chlorophenyl)(9-hydroxy-7-(2-methyl-1*H*-indol-3-yl)-2,3,4,5-tetrahydro-1*H*-[1,3]-diazepino[1,2-*a*]indol-11-yl)methanone (**3k**)

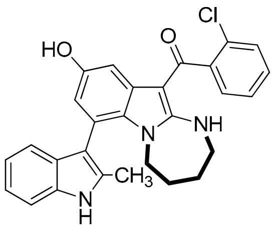

Yellow solid; Mp 240.5–241.5 °C; IR (KBr): 3398, 3063, 2937, 2347, 1726, 1597, 1439, 1336, 1176, 750 cm^{-1}; ^{1}H-NMR (500 MHz, DMSO-d_6): δ = 10.94 (br, 1H, NH), 9.21 (br, 1H, NH), 8.15 (s, 1H, ArH), 7.61 (d, *J* = 7.5 Hz, 1H, ArH), 7.46–7.56 (m, 2H, ArH), 7.35–7.41 (m, 1H, ArH), 7.30 (d, *J* = 8.0 Hz, 1H, ArH), 7.24 (d, *J* = 7.5 Hz, 1H, ArH), 7.09 (s, 1H, ArH), 7.00 (t, *J* = 7.5, 1H, ArH), 6.91 (t, *J* = 7.5 Hz, 1H, ArH), 5.69 (br, 1H, OH), 4.01–4.08 (m, 2H, NCH_2), 3.51–3.55 (m, 2H, CH_2N), 2.27 (s, 3H, CH_3), 1.98–2.02 (m, 2H, CH_2), 1.89–1.94 (m, 2H, CH_2); ^{13}C-NMR (125 MHz, DMSO-d_6): δ = 185.0, 159.8, 150.8, 142.0, 135.6, 133.3, 130.6, 130.1, 130.0, 129.5, 128.9, 128.1, 128.1, 125.4, 120.3, 118.9, 118.8, 116.0, 112.3, 110.7, 110.3, 104.6, 97.8, 45.5, 44.7, 28.8, 26.8, 13.0; HRMS (ESI-TOF): *m*/*z* calcd. for $C_{28}H_{25}ClN_3O_2$ [M + H]$^+$, 470.1630; found, 470.1621.

(4-Fluorophenyl)(9-hydroxy-7-(2-methyl-1*H*-indol-3-yl)-2,3,4,5-tetrahydro-1*H*-[1,3]-diazepino[1,2-*a*]indol-11-yl)methanone (**3l**)

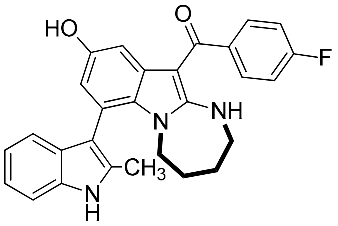

Yellow solid; Mp 259–261 °C; IR (KBr): 3390, 3064, 2929, 2343, 1720,1595, 1535, 1428, 1222, 1167, 749 cm^{-1}; ^{1}H-NMR (500 MHz, DMSO-d_6): δ = 10.91 (br, 1H, NH), 9.06 (br, 1H, NH), 8.32 (s, 1H, ArH), 7.59–7.65 (m, 2H, ArH), 7.35 (t, *J* = 8.5 Hz, 2H, ArH), 7.29 (d, *J* = 7.5 Hz, 1H, ArH), 7.24 (d, *J* = 7.5 Hz, 1H, ArH), 7.11 (s, 1H, ArH), 6.96–7.03 (m, 1H, ArH), 6.86–6.94 (m, 1H, ArH), 6.24 (br, 1H, OH), 4.04–4.08 (m, 2H, NCH_2), 3.44–3.48 (m, 2H, CH_2N), 2.28 (s, 3H, CH_3), 1.95–1.99 (m, 2H, CH_2), 1.89–1.93 (m, 2H, CH_2); ^{13}C-NMR (125 MHz, DMSO-d_6): δ = 187.4, 164.2, 162.3, 160.0, 150.7, 139.2, 135.6, 133.2, 129.9, 129.7, 129.7, 128.9, 125.5, 120.2, 118.9, 118.7, 115.8, 115.6, 112.3, 110.6, 110.5, 105.1, 97.5, 45.5, 44.9, 29.1, 26.9, 13.0; HRMS (ESI-TOF): *m*/*z* calcd. for $C_{28}H_{25}FN_3O_2$ [M + H]$^+$, 454.1925; found, 454.1936.

(7-Hydroxy-5-(2-methyl-1*H*-indol-3-yl)-2,3-dihydro-1*H*-imidazo[1,2-*a*]indol-9-yl)(4-methoxyphenyl)methanone (**3m**)

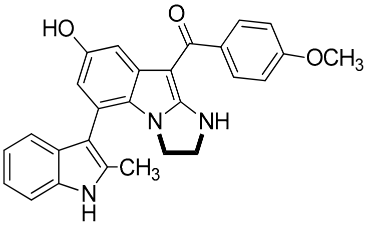

Yellow solid; Mp 269–271 °C; IR (KBr): 3390, 3059, 2966, 2843, 2353, 1726, 1597, 1473, 1325, 1248, 1163, 744 cm^{-1}; ^{1}H-NMR (500 MHz, DMSO-d_6): δ = 10.93 (br, 1H, NH), 8.40 (br, 1H, NH), 7.63–7.67 (m, 2H, ArH), 7.28–7.32 (m, 2H, ArH), 7.04–7.08 (m, 5H, ArH), 6.90–6.94 (m, 2H, ArH), 3.98–4.10 (m, 4H, CH_2CH_2), 3.85 (s, 3H, OCH_3), 2.32 (s, 3H, CH_3); ^{13}C-NMR (125 MHz, DMSO-d_6): δ = 186.5, 161.2, 159.5, 150.4, 135.6, 134.8, 133.1, 130.7, 129.7, 129.7, 128.9, 126.0, 120.2, 118.9, 118.7, 115.0, 114.0, 114.0, 111.1, 110.7, 106.8, 93.4, 55.6, 49.5, 42.5, 13.0; HRMS (ESI-TOF): *m*/*z* calcd. for $C_{27}H_{24}N_3O_3$ [M + H]$^+$, 438.1812; found, 438.1786.

(7-Hydroxy-5-(2-methyl-1*H*-indol-3-yl)-2,3-dihydro-1*H*-imidazo[1,2-*a*]indol-9-yl)(*p*-tolyl)methanone (**3n**)

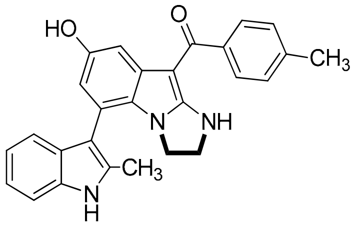

Yellow solid; Mp 298.5–300 °C; IR (KBr): 3408, 2899, 2584, 2345, 1726, 1597, 1475, 1327, 1167, 752 cm^{-1}; ^{1}H-NMR (300 MHz, DMSO-d_6): δ = 10.91 (br, 1H, NH), 8.38 (br, 1H, NH), 7.53 (d, *J* = 6.5 Hz, 2H, ArH), 7.25–7.34 (m, 4H, ArH), 6.88–7.02 (m, 5H, ArH), 3.97–4.09 (m, 4H, CH_2CH_2), 2.40 (s, 3H, $ArCH_3$), 2.29 (s, 3H, CH_3); ^{13}C-NMR (125 MHz, DMSO-d_6): δ = 187.2, 159.7, 150.4, 140.1, 139.6, 135.6, 133.1, 130.5, 129.3, 129.3, 128.9, 127.6, 127.6, 126.0, 120.3, 118.9, 118.7, 115.2, 111.1, 110.7, 110.5, 106.9, 93.5, 49.5, 42.5, 21.5, 13.0; HRMS (ESI-TOF): *m*/*z* calcd. for $C_{27}H_{24}N_3O_2$ [M + H]$^+$, 422.1863; found, 422.1837.

(7-Hydroxy-5-(2-methyl-1*H*-indol-3-yl)-2,3-dihydro-1*H*-imidazo[1,2-*a*]indol-9-yl)-(phenyl)methanone (**3o**)

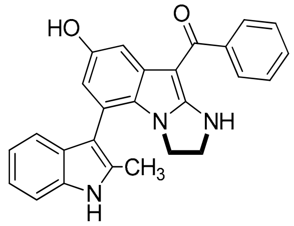

Yellow solid; Mp 289.5–290.5 °C; IR (KBr): 3419, 3059, 2970, 2316, 1730, 1603, 1510, 1335, 1227, 744 cm^{-1}; ^{1}H-NMR (500 MHz, DMSO-d_6): δ= 10.92 (br, 1H, NH), 8.33 (br, 1H, NH), 7.61–7.65 (m, 2H, ArH), 7.52–7.56 (m, 3H, ArH), 7.28–7.32 (m, 2H, ArH), 6.91–7.02 (m, 5H, ArH), 3.99–4.11 (m, 4H, CH_2CH_2), 2.31 (s, 3H, CH_3); ^{13}C-NMR (125 MHz, DMSO-d_6): δ = 187.1, 159.7, 150.4, 142.6, 135.6, 133.1, 130.8, 130.4, 130.3, 128.8, 128.8, 127.5, 127.5, 126.1, 120.2, 118.9, 118.7, 115.2, 111.1, 110.7, 110.5, 106.8, 93.5, 49.5, 42.5, 13.0; HRMS (ESI-TOF): *m*/*z* calcd. for $C_{26}H_{22}N_3O_2$ [M + H]$^+$, 408.1707; found, 408.1713.

(4-Chlorophenyl)(7-hydroxy-5-(2-methyl-1*H*-indol-3-yl)-2,3-dihydro-1*H*-imidazo[1,2-*a*]indol-9-yl)methanone (**3p**)

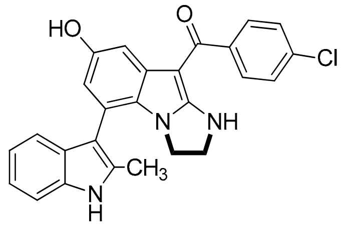

Yellow solid; Mp 204–206 °C; IR (KBr): 3435, 3072, 2902, 2347, 1724, 1600, 1510, 1402, 1330, 1223, 752 cm^{-1}; ^{1}H-NMR (500 MHz, DMSO-d_6): δ = 10.95 (br, 1H, NH), 8.48 (br, 1H, NH), 7.60–7.66 (m, 2H, ArH), 7.54–7.60 (m, 2H, ArH), 7.25–7.32 (m, 3H, ArH), 7.00 (t, *J* = 7.0 Hz, 1H, ArH), 6.89–6.95 (m, 3H, ArH), 4.05–4.11 (m, 2H, NCH_2), 3.98–4.03 (m, 2H, CH_2N), 2.30 (s, 3H, CH_3); ^{13}C-NMR (125 MHz, DMSO-d_6): δ = 185.6, 159.7, 150.5, 141.2, 135.6, 134.9, 133.1, 130.3, 129.5, 129.5, 128.9, 128.9, 128.9, 126.0, 120.2, 118.9, 118.7, 115.3, 111.2, 110.7, 110.5, 106.7, 93.3, 49.5, 42.5, 13.0; HRMS (ESI-TOF): *m*/*z* calcd. for $C_{26}H_{21}ClN_3O_2$ [M + H]$^+$, 442.1317; found, 442.1309.

(2-Chlorophenyl)(7-hydroxy-5-(2-methyl-1*H*-indol-3-yl)-2,3-dihydro-1*H*-imidazo[1,2-*a*]indol-9-yl)methanone (**3q**)

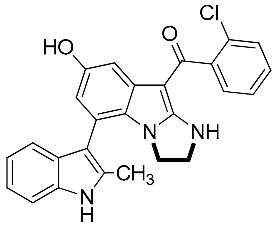

Yellow solid; mp 339–341 °C; IR (KBr): 3429, 3346, 3059, 2918, 2580, 2318, 1728, 1520, 1464, 1327, 1225, 748 cm^{-1}; ^{1}H-NMR (500 MHz, DMSO-d_6): δ = 10.92 (br, 1H, NH), 7.87–8.07 (m, 1H, ArH), 7.10–7.60 (m, 8H, ArH), 6.87–7.02 (m, 3H, ArH), 4.08– 4.12(m, 4H, CH_2CH_2), 2.26 (s, 3H, CH_3); ^{13}C-NMR (125 MHz, DMSO-d_6): δ = 184.0, 160.1, 150.1, 142.1, 135.6, 133.2, 130.6, 130.2, 129.5, 128.8, 128.1, 128.1, 128.1, 126.1, 120.3, 118.8, 118.8, 115.4, 111.2, 110.7, 110.3, 105.7, 94.1, 49.6, 42.4, 12.9; HRMS (ESI-TOF): *m*/*z* calcd. for $C_{26}H_{21}ClN_3O_2$ [M + H]$^+$, 442.1317; found, 442.1302.

(4-Fluorophenyl)(7-hydroxy-5-(2-methyl-1*H*-indol-3-yl)-2,3-dihydro-1*H*-imidazo[1,2-*a*]indol-9-yl)methanone (**3r**)

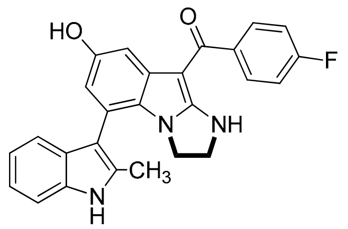

Yellow solid; Mp 268–270 °C; IR (KBr): 3429, 3072, 2902, 2582, 2347, 1724, 1601, 1510, 1402, 1331, 1223, 1157, 752 cm^{-1}; ^{1}H-NMR (500 MHz, DMSO-d_6): δ = 10.92 (br, 1H, NH), 8.39 (br, 1H, NH), 7.65–7.72 (m, 2H, ArH), 7.27–7.37 (m, 4H, ArH), 6.89–7.04 (m, 5H, ArH), 4.06–4.13 (m, 2H, NCH_2), 3.98–4.04 (m, 2H, CH_2N), 2.31 (s, 3H, CH_3); ^{13}C-NMR (125 MHz, DMSO-d_6): δ = 185.8, 164.4, 162.4, 159.7, 150.5, 139.0, 135.6, 133.1, 130.4, 130.0, 130.0, 128.9, 126.0, 120.2, 118.9, 118.7, 115.8, 115.6, 115.2, 111.2, 110.7, 106.7, 93.3, 49.5, 42.5, 13.0; HRMS (ESI-TOF): *m*/*z* calcd. for $C_{26}H_{21}FN_3O_2$ [M + H]$^+$, 426.1612; found, 426.1622.

(6-(1,2-Dimethyl-1*H*-indol-3-yl)-8-hydroxy-1,2,3,4-tetrahydropyrimido[1,2-*a*]indol-10-yl)(4-methoxyphenyl)methanone (**3s**)

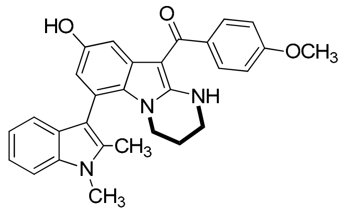

Yellow solid; Mp 261–263 °C; IR (KBr) 3439, 2926, 2853, 2347, 1728, 1616, 1510, 1471, 1350, 1324, 1168, 740 cm^{-1}; ^{1}H-NMR ($CDCl_3$, 400 MHz): δ = 8.61 (br, 1H, NH), 7.69 (d, *J* = 8.8 Hz, 2H, ArH), 7.39 (d, *J* = 8.0 Hz, 1H, ArH), 7.34 (d, *J* = 8.0 Hz, 1H, ArH), 7.20–7.22 (m, 1H, ArH), 7.07–7.11 (m, 1H, ArH), 6.98 (d, *J* = 8.4 Hz, 2H, ArH), 6.86 (s, 1H, ArH), 6.71 (s, 1H, ArH), 4.93 (br, 1H, OH), 3.90–3.93 (m, 2H, CH_2N), 3.86 (s, 3H, NCH_3), 3.75 (s, 3H, OCH_3), 3.53–3.57 (m, 2H, CH_2), 2.34 (s, 3H, CH_3), 2.21–2.24 (m, 2H, CH_2N); ^{13}C-NMR ($CDCl_3$, 100 MHz): δ = 188.8, 161.0, 153.5, 149.3, 137.0, 135.4, 134.9, 129.4, 129.4, 129.3, 127.5, 126.6, 121.5, 119.9, 118.9, 113.6, 113.6, 113.2, 109.5, 108.9, 107.7, 104.7, 95.8, 55.3, 39.3, 38.1, 29.9, 20.8, 11.1; HRMS (ESI-TOF): *m*/*z* calcd for $C_{29}H_{27}N_3O_3$ [M + H]$^+$, 466.2125; found, 466.2120.

(6-(1,2-Dimethyl-1*H*-indol-3-yl)-8-hydroxy-1,2,3,4-tetrahydropyrimido[1,2-*a*]indol-10-yl)(4-methoxyphenyl)methanone(6-(1,2-dimethyl-1*H*-indol-3-yl)-8-hydroxy-1,2,3,4-tetrahydropyrimido[1,2-*a*]indol-10-yl)(4-fluorophenyl)methanone (**3t**)

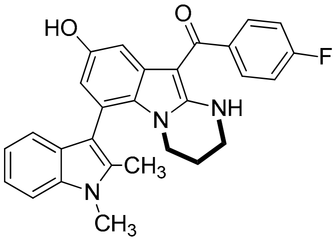

Yellow solid; Mp 173–175 °C; IR (KBr) 3437, 2925, 2582, 1721, 1617, 1534, 1470, 1325, 1221, 1173, 775 cm^{-1}; ^1H-NMR ($CDCl_3$, 400 MHz): δ = 8.62 (br, 1H, NH), 7.67–7.71 (m, 2H, ArH), 7.34–7.39 (m, 2H, ArH), 7.11–7.26 (m, 3H, ArH), 7.08–7.11 (m, 1H, ArH), 6.87 (s, 1H, ArH), 6.53 (s, 1H, ArH), 4.88 (br, 1H, OH), 3.94 (t, *J* = 6.0, 2H, CH_2N), 3.77 (s, 3H, NCH_3), 3.57–3.63 (m, 2H, CH_2), 2.35 (s, 3H, CH_3), 2.23–2.29 (m, 2H, CH_2N); ^{13}C-NMR ($CDCl_3$, 100 MHz): δ = 188.0, 165.5, 162.5, 153.6, 149.4, 138.5, 137.0, 135.4, 129.6, 129.5, 129.4, 127.4, 126.4, 121.6, 120.0, 118.8, 115.5, 115.3, 113.4, 109.6, 108.9, 107.6, 104.5, 95.9, 39.3, 38.1, 29.9, 20.7, 11.1; HRMS (ESI-TOF): *m*/*z* calcd for $C_{28}H_{24}N_3O_2$ [M + H]$^+$, 454.1925; found, 454.1931.

(8-Hydroxy-6-(2-phenyl-1*H*-indol-3-yl)-1,2,3,4-tetrahydropyrimido[1,2-*a*]indol-10-yl)(4-methoxyphenyl)methanone (**3u**)

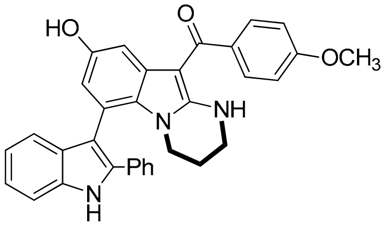

Yellow solid; Mp 179–181 °C; IR (KBr) 3438, 2925, 2854, 1728, 1616, 1577, 1532, 1445, 1326, 1253, 1168, 747 cm^{-1}; ^1H-NMR ($CDCl_3$, 400 MHz) δ = 8.92 (br, 1H, NH), 8.59 (br, 1H, NH), 7.69 (d, *J* = 8.8 Hz, 2H, ArH), 7.45–7.40 (m, 4H, ArH), 7.28–7.22 (m, 4H, ArH), 7.13–7.10 (m, 1H, ArH), 6.96 (d, *J* = 8.8 Hz, 2H, ArH), 6.85 (s, 1H, ArH), 6.72 (s, 1H, ArH), 4.94 (br, 1H, OH), 3.82–3.86 (m, 5H, CH_2N, OCH_3), 3.47–3.51 (m, 2H, NCH_2), 2.19–2.16 (m, 2H, CH_2); ^{13}C-NMR ($CDCl_3$, 100 MHz) δ = 188.9, 161.1, 153.6, 149.3, 136.2, 135.2, 134.8, 131.9, 129.7, 129.5, 129.4, 129.4, 128.9, 128.9, 127.9, 127.0, 127.0, 123.1, 120.6, 119.8, 113.6, 113.6, 113.1, 113.0, 111.1, 109.5, 108.8, 105.2, 95.9, 55.3, 39.2, 38.0, 20.7; HRMS (ESI-TOF): *m*/*z* calcd for $C_{33}H_{27}N_3O_3$ [M + H]$^+$, 514.2125; found, 514.2121.

(4-Fluorophenyl)(8-hydroxy-6-(2-phenyl-1*H*-indol-3-yl)-1,2,3,4-tetrahydropyrimido-[1,2-*a*]indol-10-yl)methanone (**3v**)

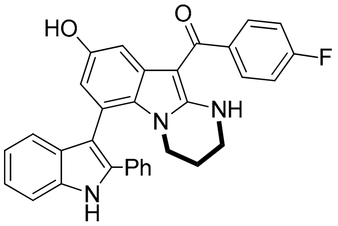

Yellow solid; Mp 264–266 °C; IR (KBr) 3426, 2924, 1721, 1617, 1535, 1478, 1325, 1221, 1176, 774 cm^{-1}; ^1H-NMR ($CDCl_3$, 400 MHz): δ = 8.75 (br, 1H, NH), 8.61 (br, 1H, NH), 7.71–7.67 (m, 2H, ArH), 7.44–7.40 (m, 4H, ArH), 7.31–7.26 (m, 4H, ArH), 7.16–7.11 (m, 3H, ArH), 6.85 (s, 1H, ArH), 6.54 (s, 1H, ArH), 4.92 (br, 1H, OH), 3.86–3.83 (m, 2H, CH_2N), 3.52–3.56 (m, 2H, NCH_2), 2.22–2.19 (m, 2H, CH_2); ^{13}C-NMR ($CDCl_3$, 100 MHz): δ = 187.9, 165.0, 162.5, 153.7, 149.4, 138.4, 138.4, 136.1, 135.2, 131.9, 129.7, 129.6, 129.5, 128.9, 128.0, 126.9, 126.7, 123.2, 120.7, 119.8, 115.5, 115.3, 113.2, 111.1, 109.7, 108.7, 105.0,

104.9, 95.9, 39.2, 38.0, 20.4; HRMS (ESI-TOF): *m*/*z* calcd for $C_{32}H_{24}FN_3O_2$ [M + H]$^+$, 502.1925; found, 502.1933.

(8-Hydroxy-6-(5-methoxy-1*H*-indol-3-yl)-1,2,3,4-tetrahydropyrimido[1,2-*a*]indol-10-yl)(4-methoxyphenyl)methanone (**3w**)

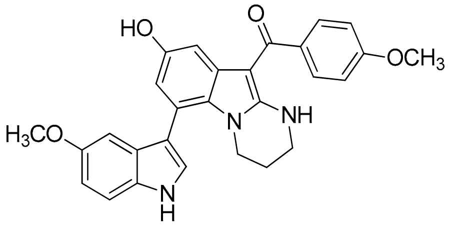

Yellow solid; Mp 184–186 °C; IR (KBr) 3430, 2921, 2852, 1724, 1612, 1557, 1528, 1450, 1340, 1250, 1170, 750 cm^{-1}; ^{1}H-NMR ($CDCl_3$, 400 MHz) δ = 8.60 (br, 1H, NH), 8.42 (br, 1H, NH), 7.70–7.68 (m, 1H, ArH), 7.64–7.61 (m, 2H, ArH), 7.35–7.33 (m, 1H, ArH), 7.01–6.94 (m, 5H, ArH), 6.70 (s, 1H, ArH), 5.08 (br, 1H, OH), 3.98–3.94 (m, 2H, CH_2N), 3.88 (s, 3H, OCH_3), 3.80 (s, 3H, OCH_3), 3.58–3.56 (m, 2H, NCH_2), 2.27–2.23 (m, 2H, CH_2); ^{13}C-NMR ($CDCl_3$, 100 MHz) δ = 188.8, 161.0, 154.8, 153.6, 148.9, 137.8, 134.7, 131.5, 129.4, 129.4, 126.5, 123.9, 118.8, 116.3, 113.6, 113.5, 112.3, 108.7, 107.6, 105.3, 105.1, 101.1, 95.8, 55.9, 55.3, 39.2, 38.0, 20.7; HRMS (ESI-TOF): *m*/*z* calcd for $C_{28}H_{25}N_3O_4$ [M + H]$^+$, 468.1918; found, 468.1922.

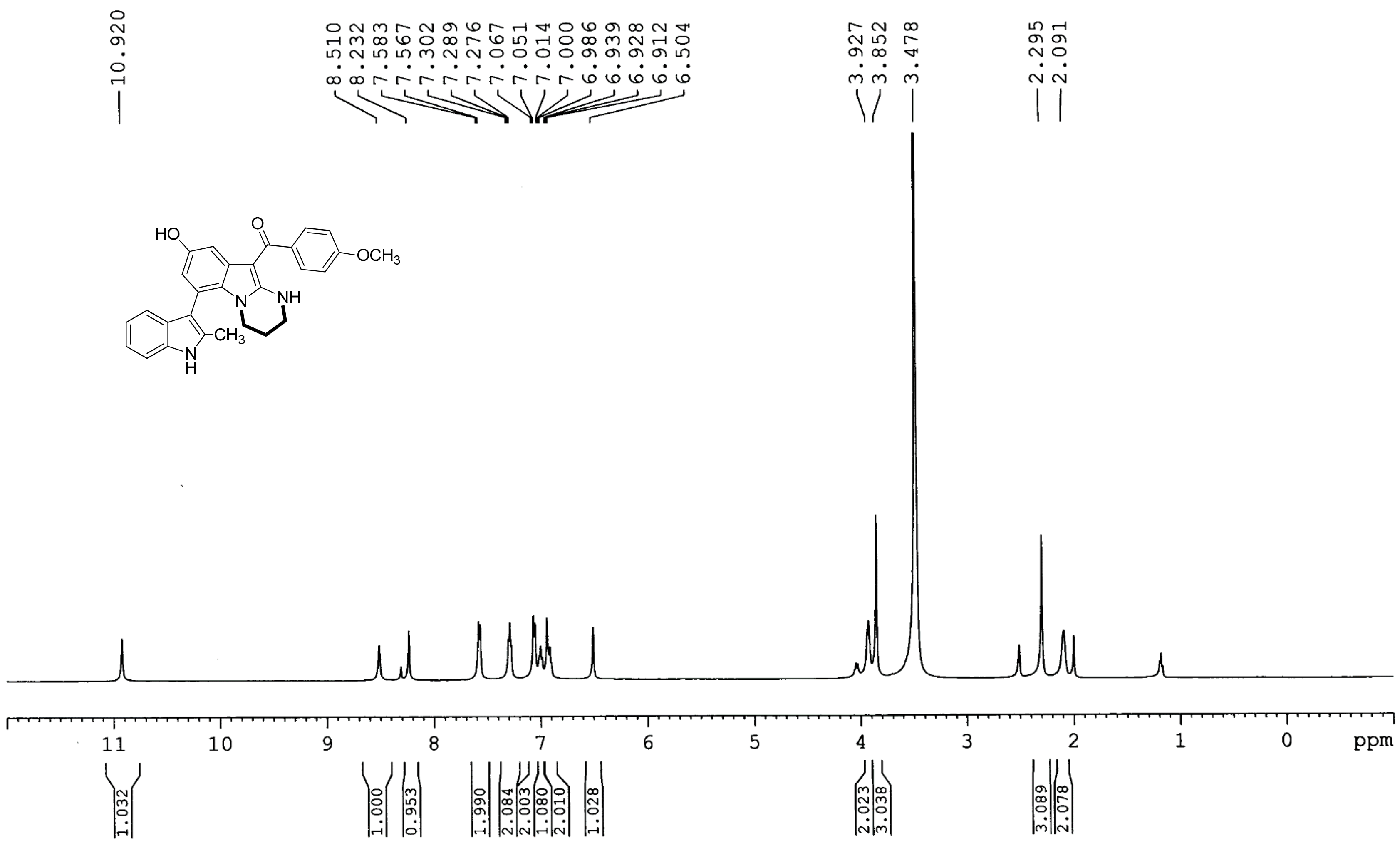

Figure S1. ^{1}H-NMR (500 MHz, DMSO-d_6) spectra of compound **3a**.

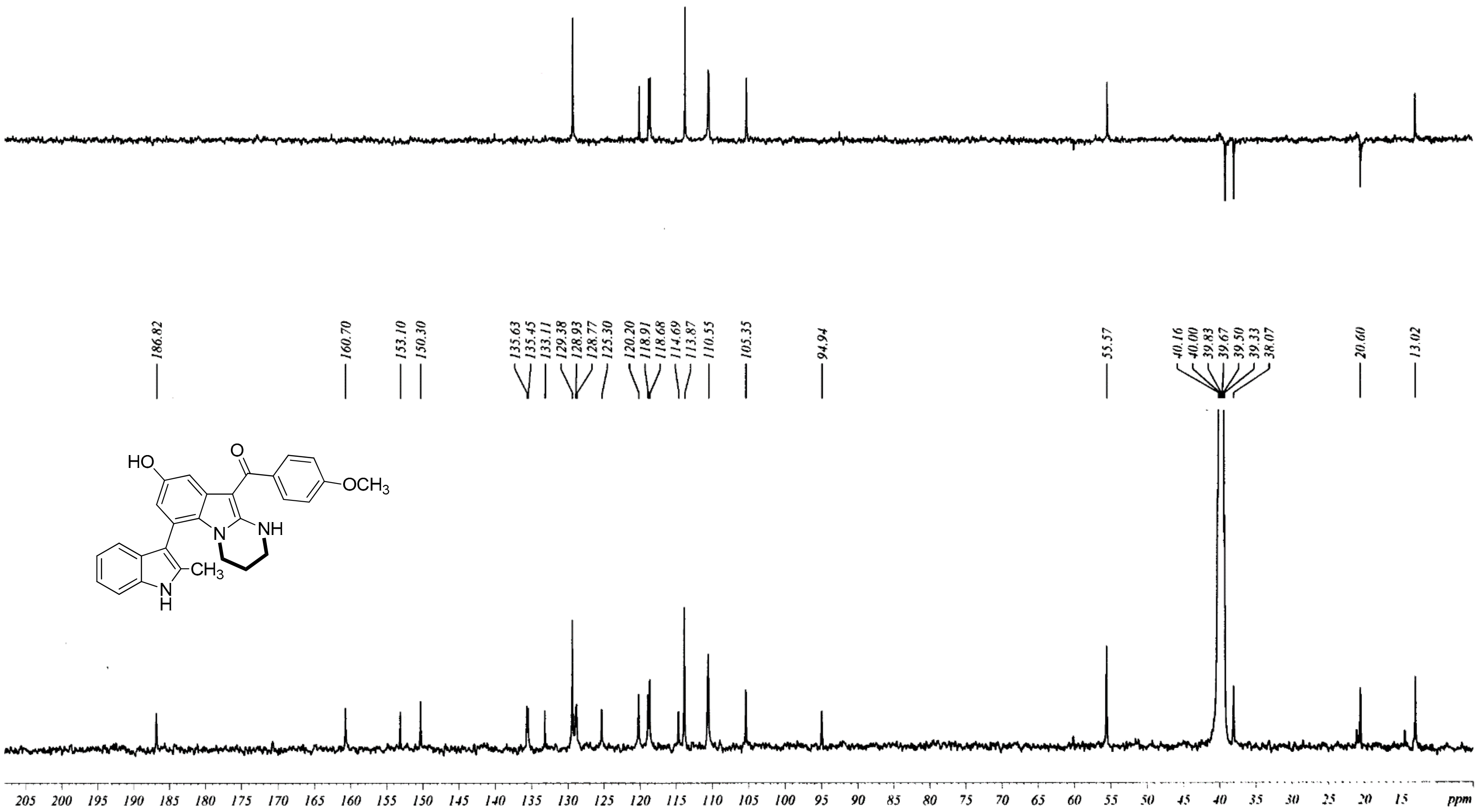

Figure S2. ^{13}C-NMR (125 MHz, DMSO-d_6) spectra of compound **3a.**

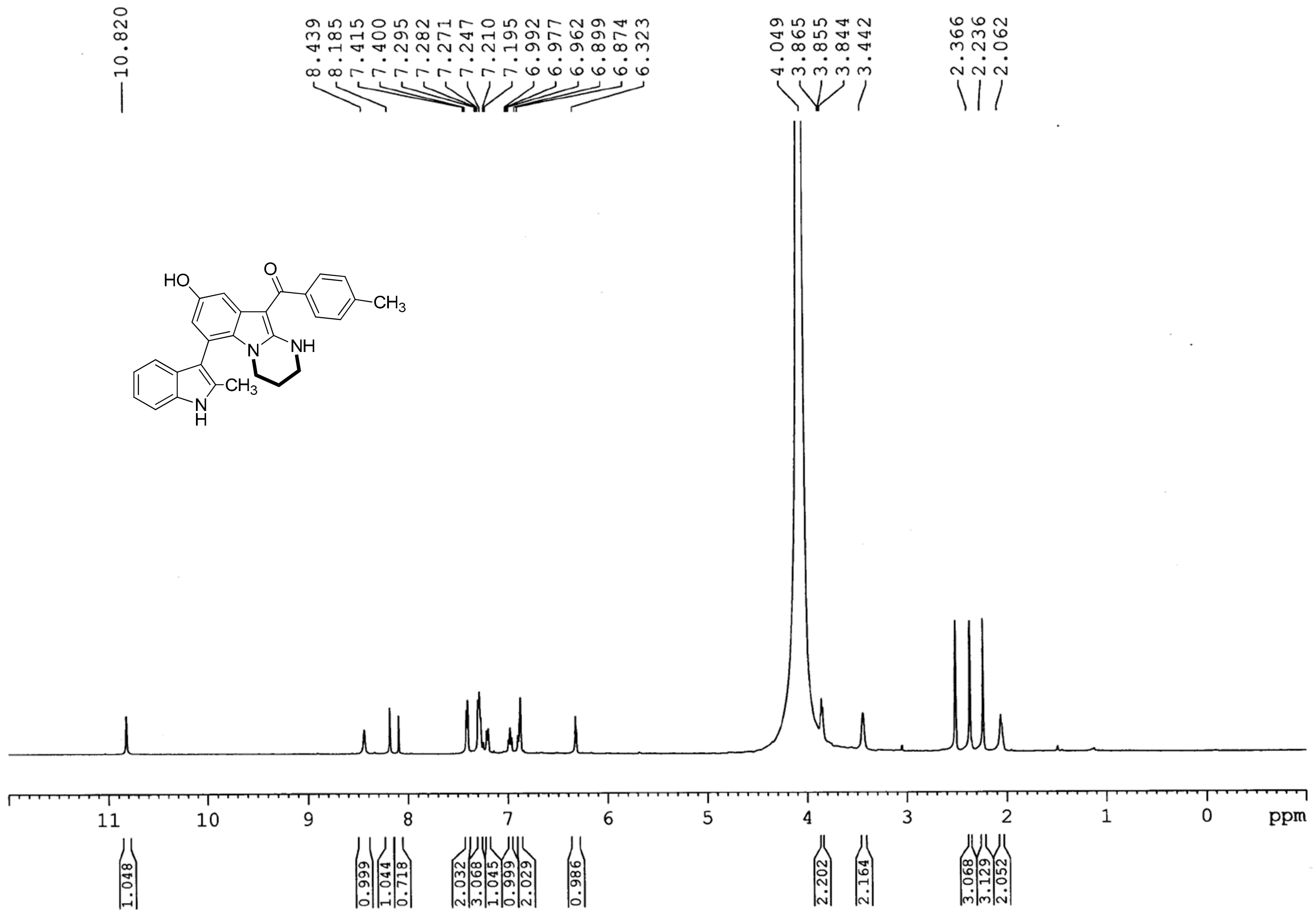

Figure S3. ^{1}H-NMR (500 MHz, DMSO-d_6) spectra of compound **3b**.

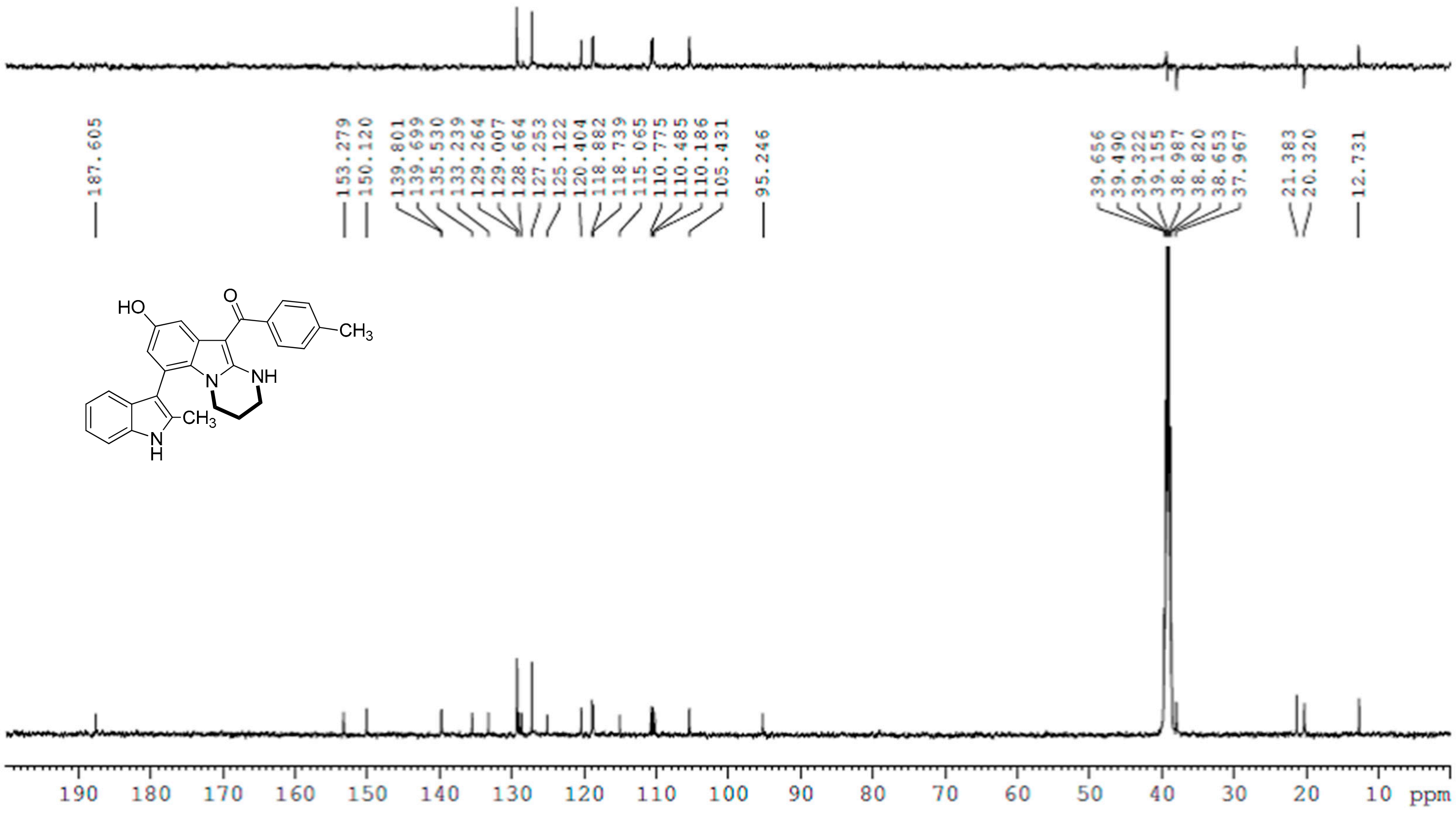

Figure S4. ^{13}C-NMR (125 MHz, DMSO-d_6) spectra of compound **3b.**

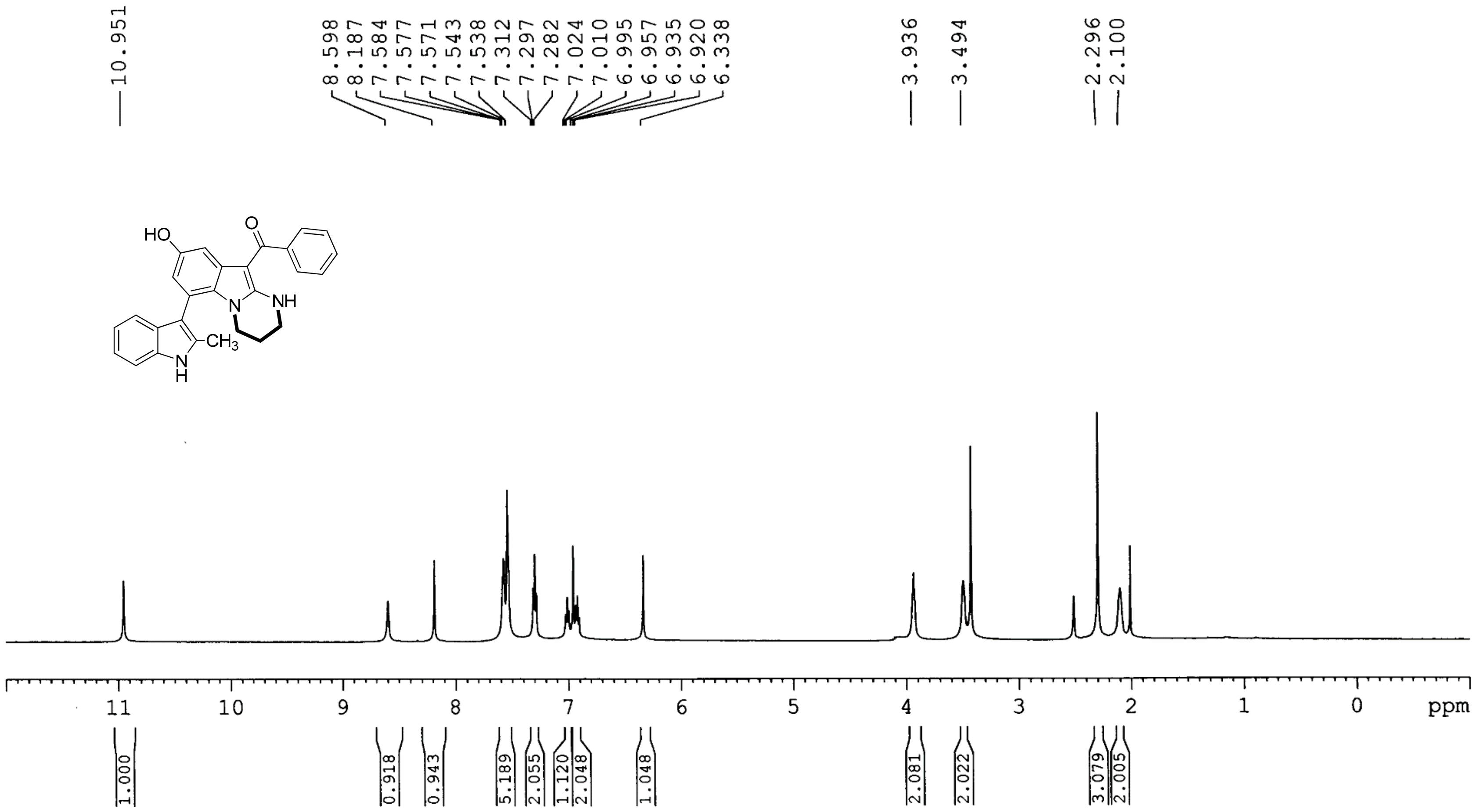

Figure S5. ^{1}H-NMR (500 MHz, DMSO-d_6) spectra of compound **3c**.

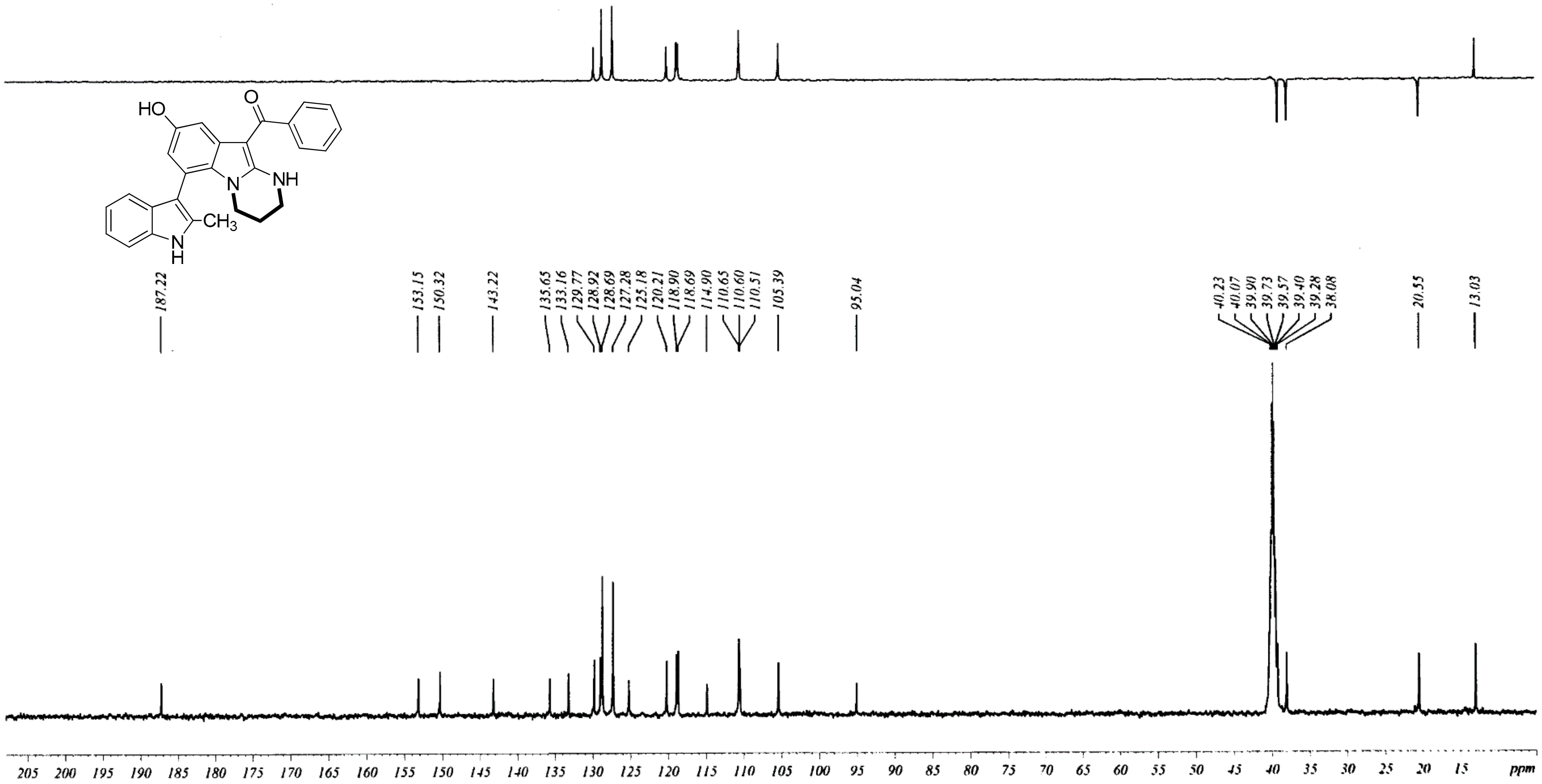

Figure S6. ^{13}C-NMR (125 MHz, DMSO-d_6) spectra of compound **3c.**

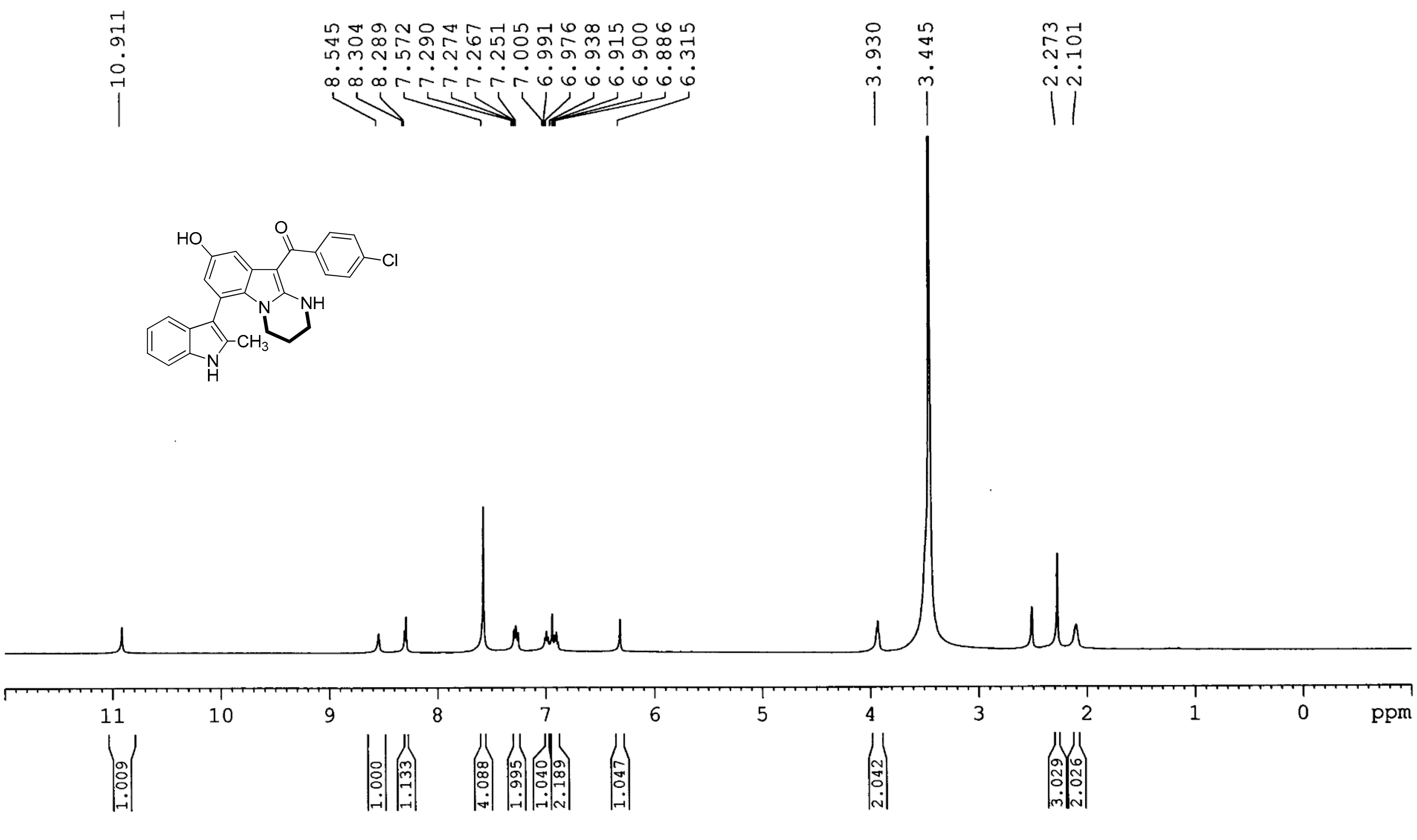

Figure S7. ^{1}H-NMR (500 MHz, DMSO-d_6) spectra of compound **3d**.

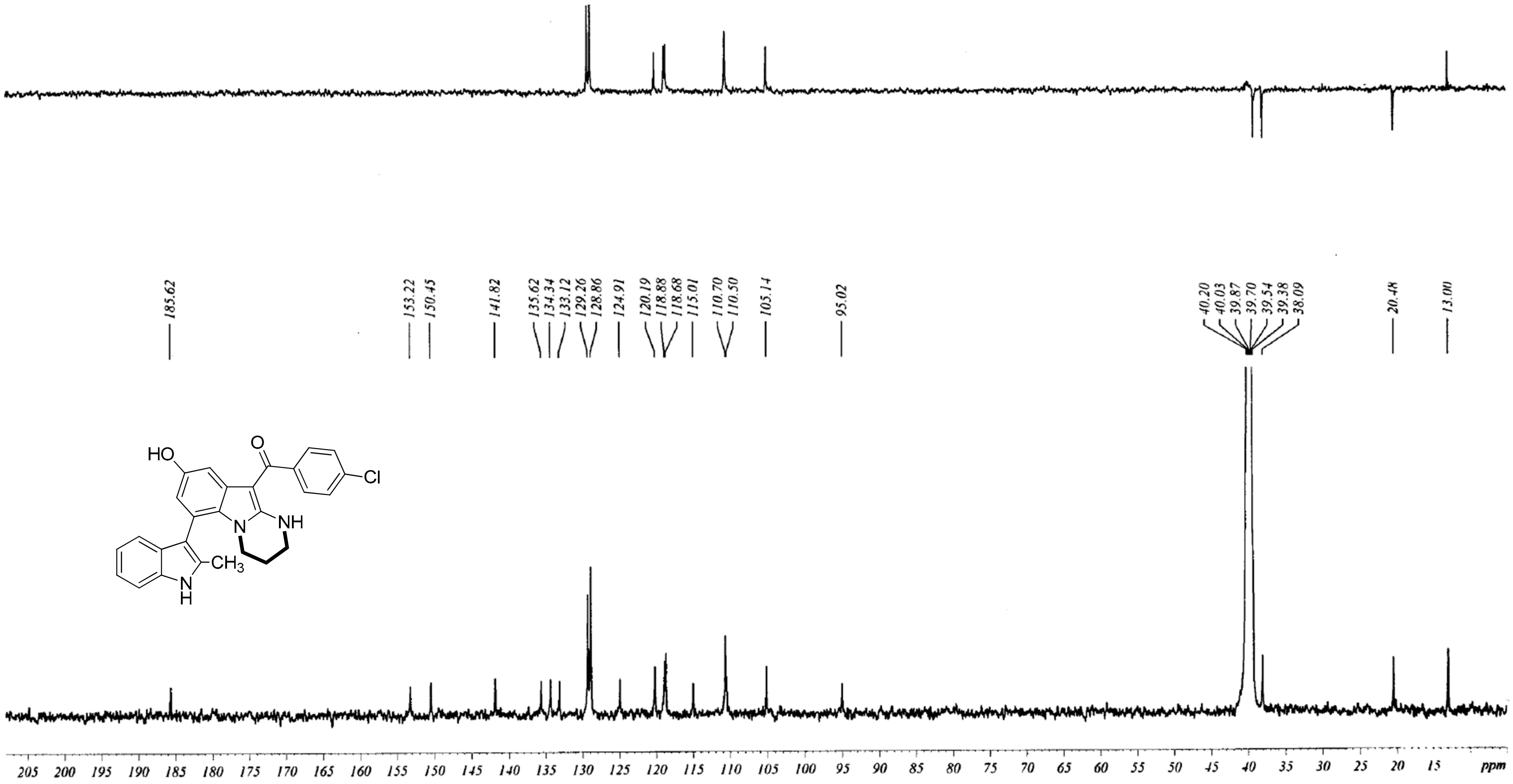

Figure S8. ^{13}C-NMR (125 MHz, DMSO-d_6) spectra of compound **3d.**

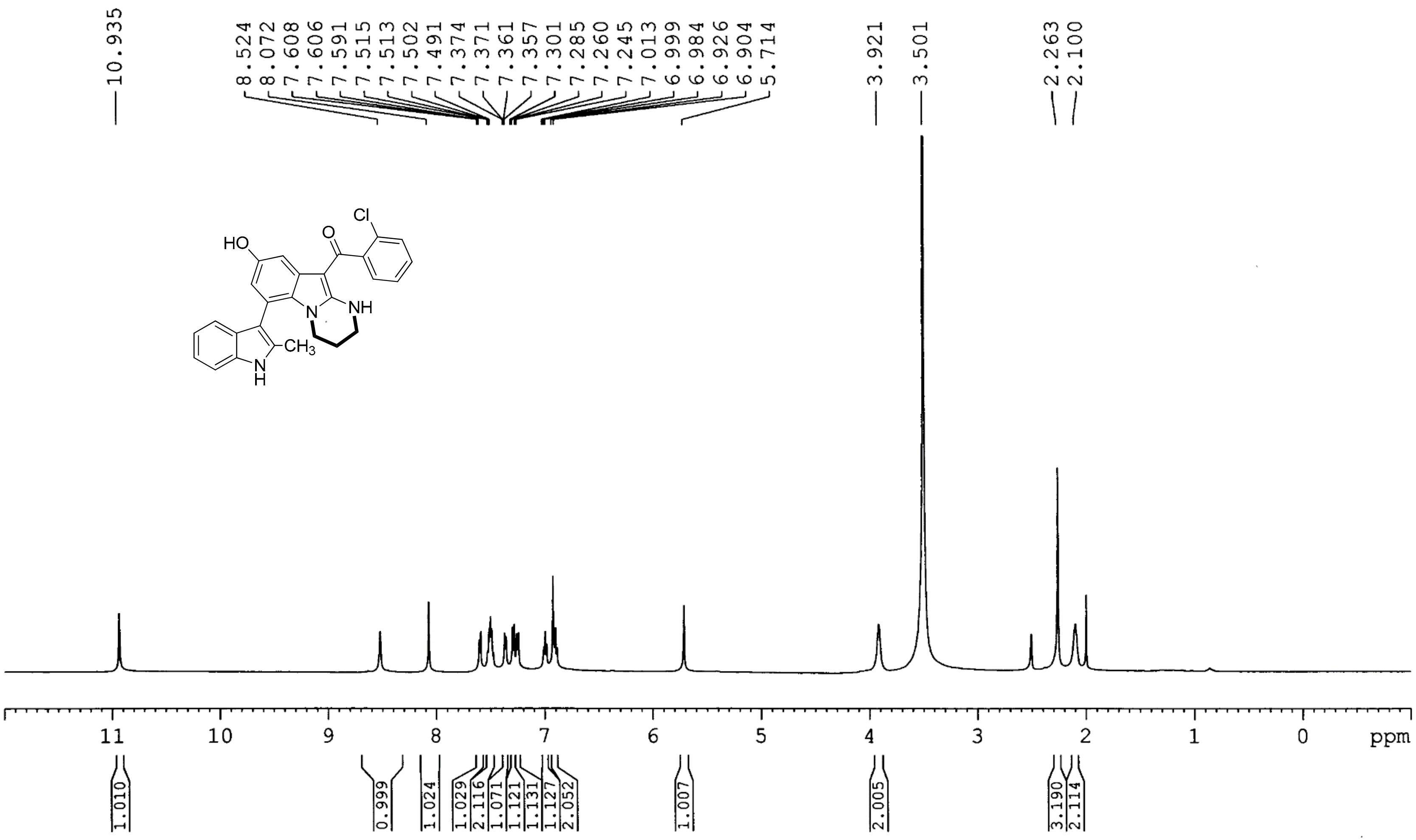

Figure S9. ^{1}H-NMR (500 MHz, DMSO-d_6) spectra of compound **3e**.

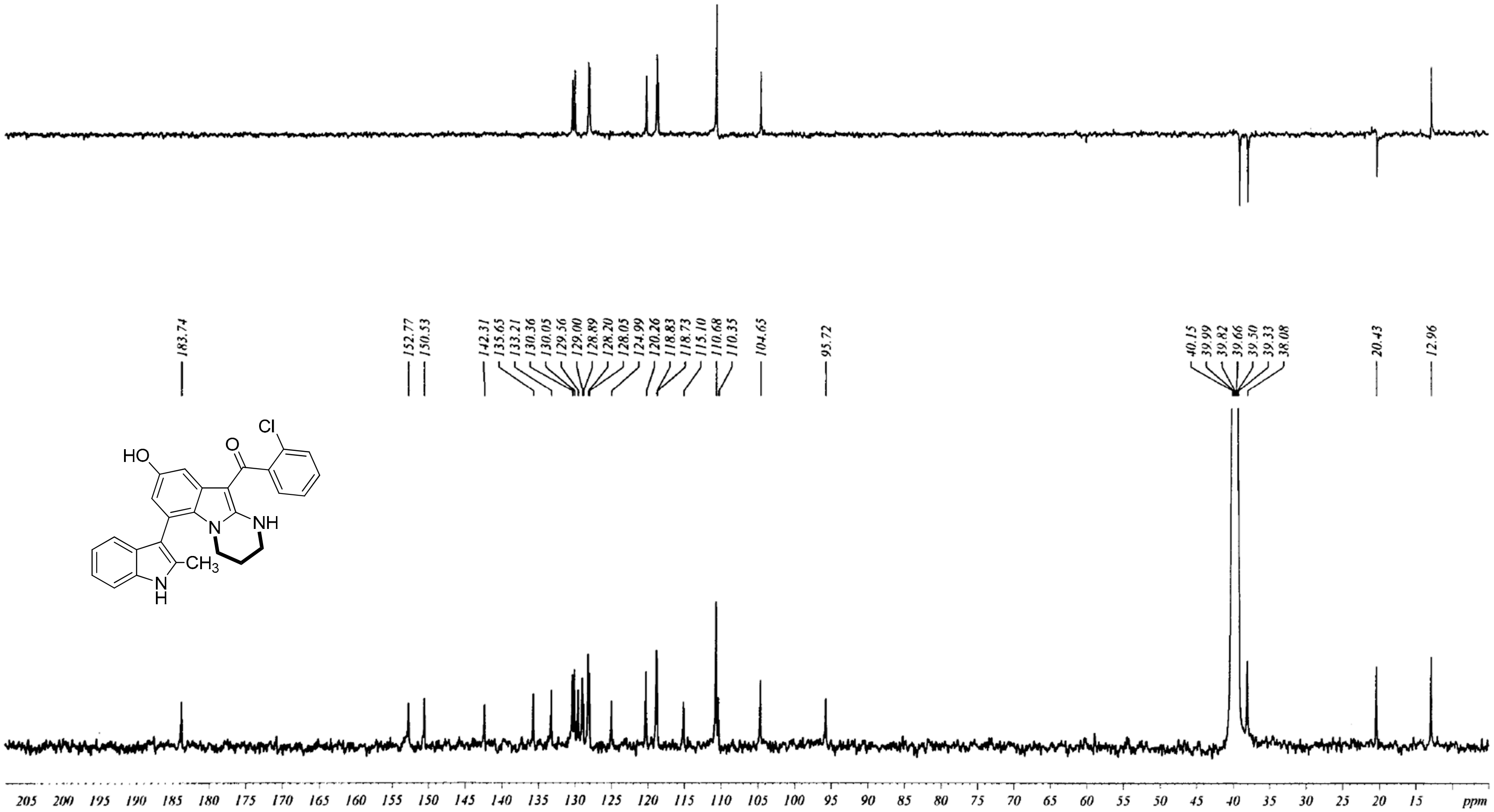

Figure S10. ^{13}C-NMR (125 MHz, DMSO-d_6) spectra of compound **3e.**

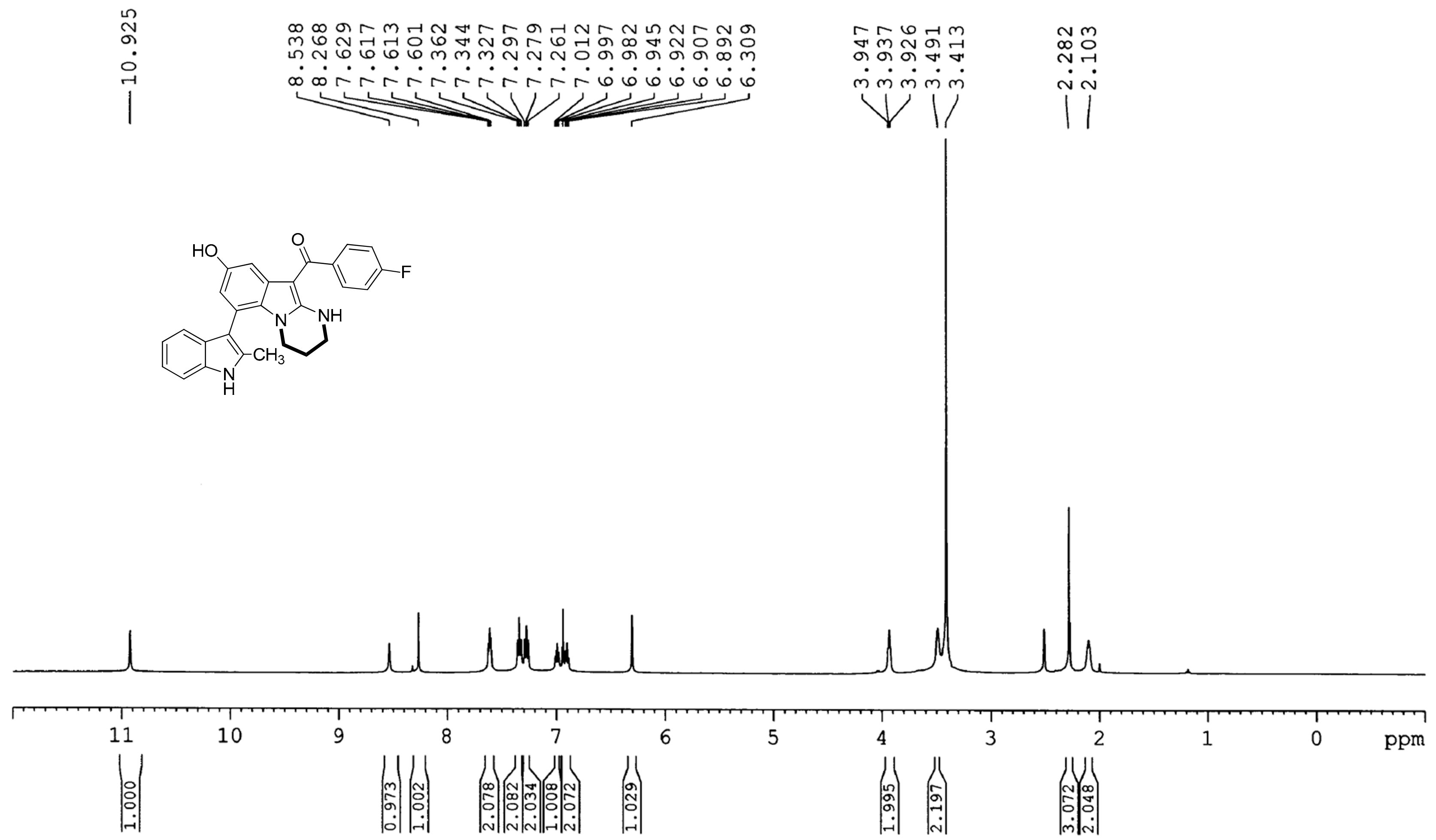

Figure S11. ^{1}H-NMR (500 MHz, DMSO-d_6) spectra of compound **3f**.

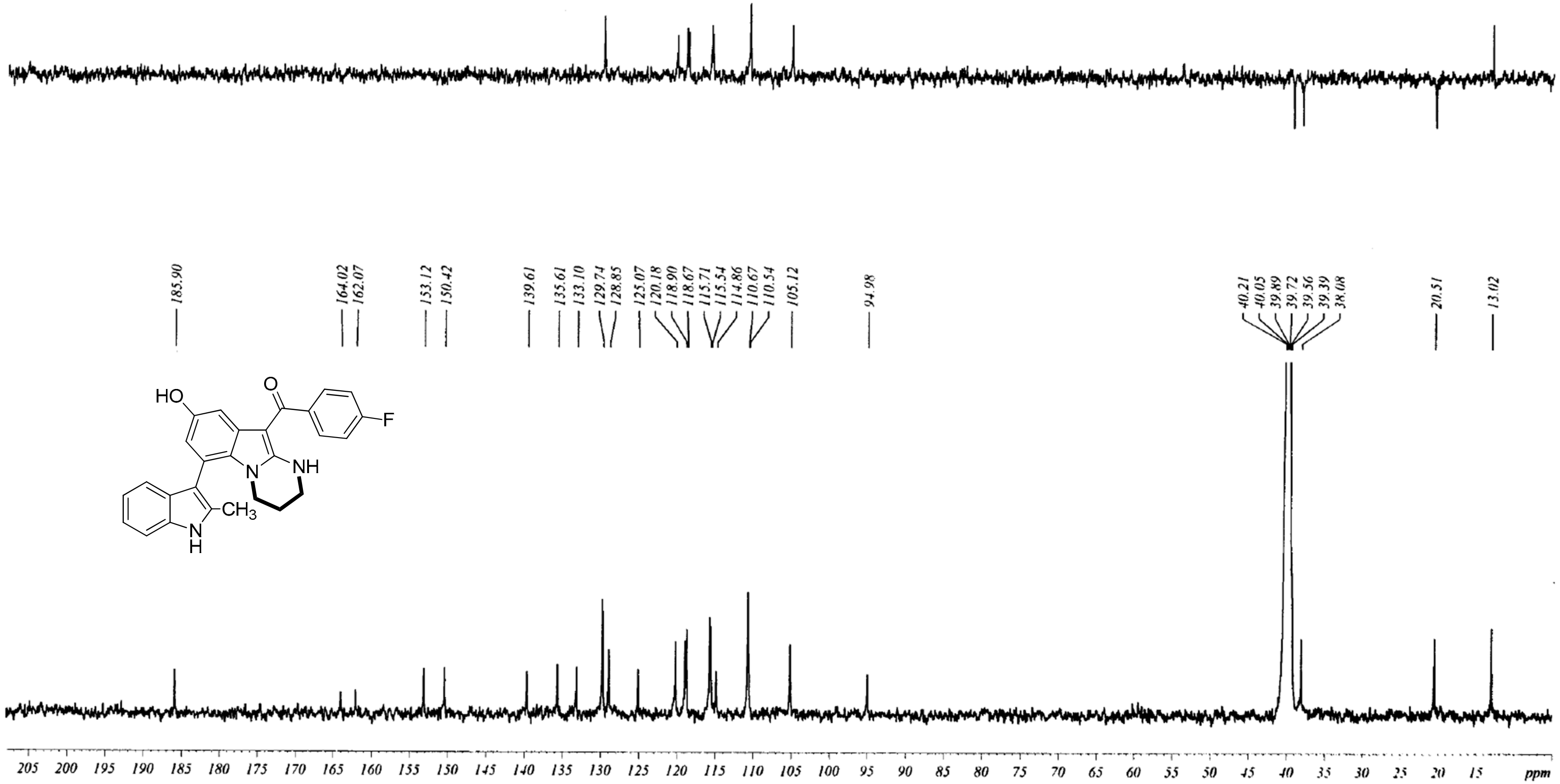

Figure S12. ^{13}C-NMR (125 MHz, DMSO-d_6) spectra of compound **3f.**

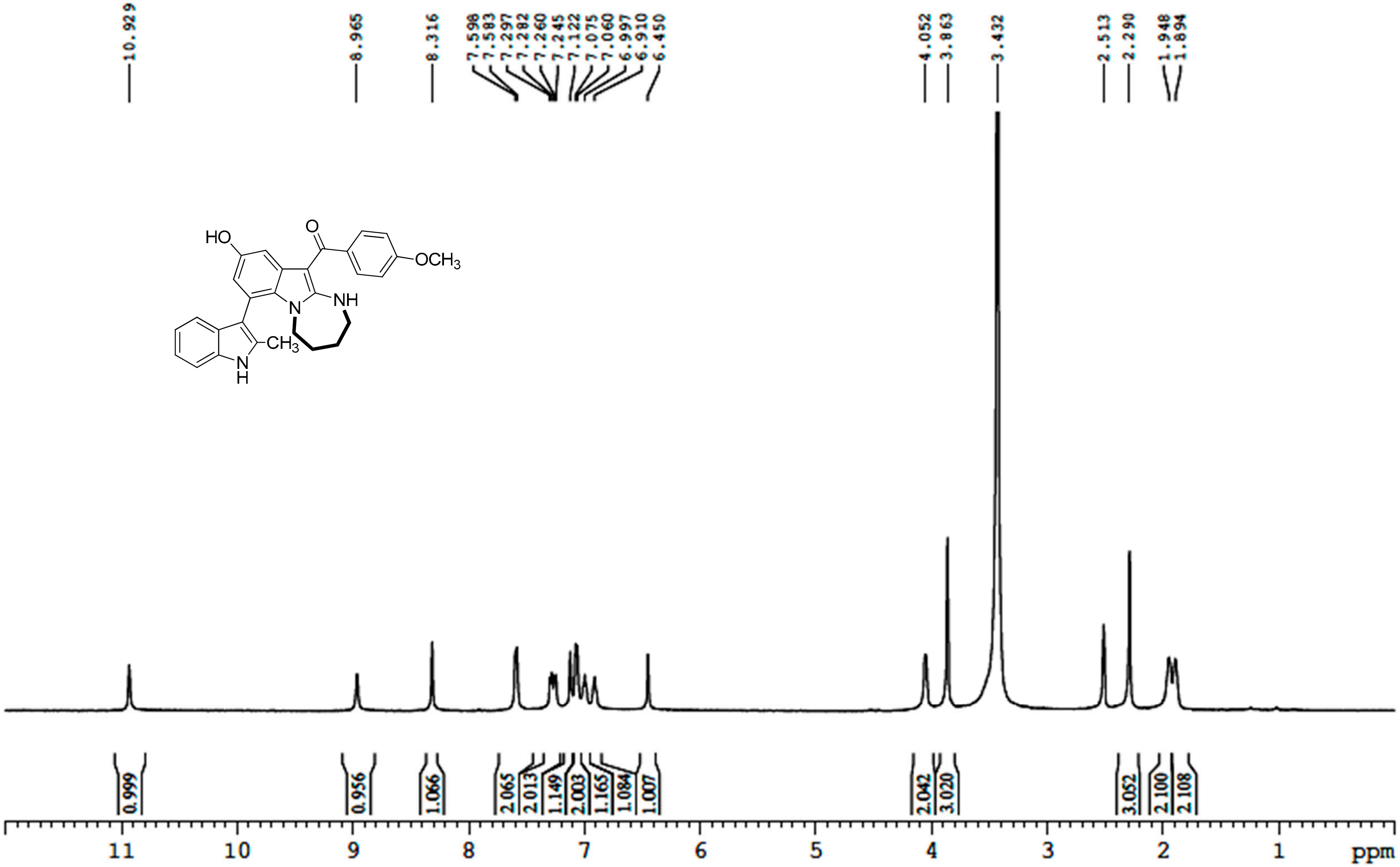

Figure S13. ^{1}H-NMR (500 MHz, DMSO-d_6) spectra of compound **3g.**

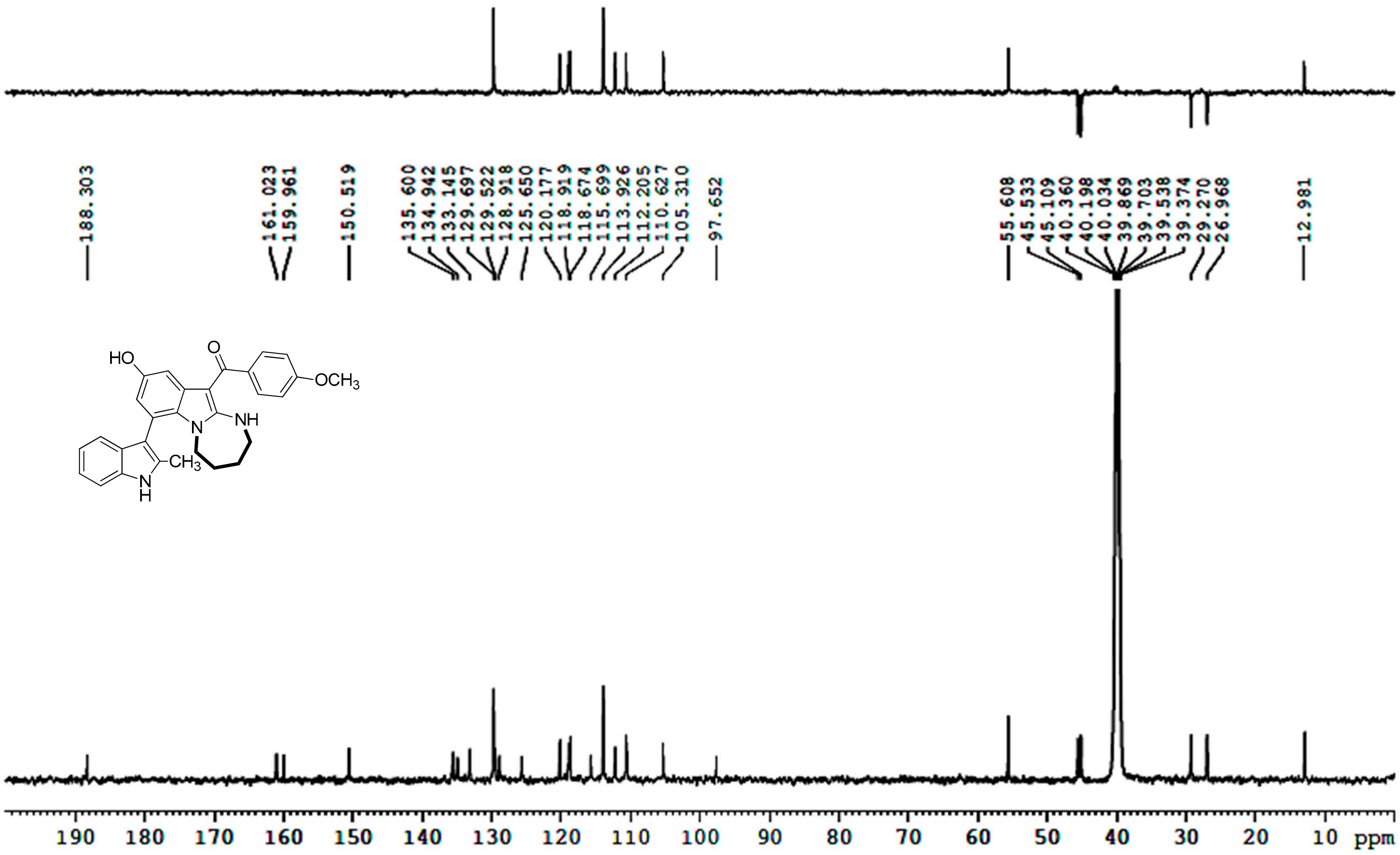

Figure S14. ^{13}C-NMR (125 MHz, DMSO-*d*$_6$) spectra of compound **3g.**

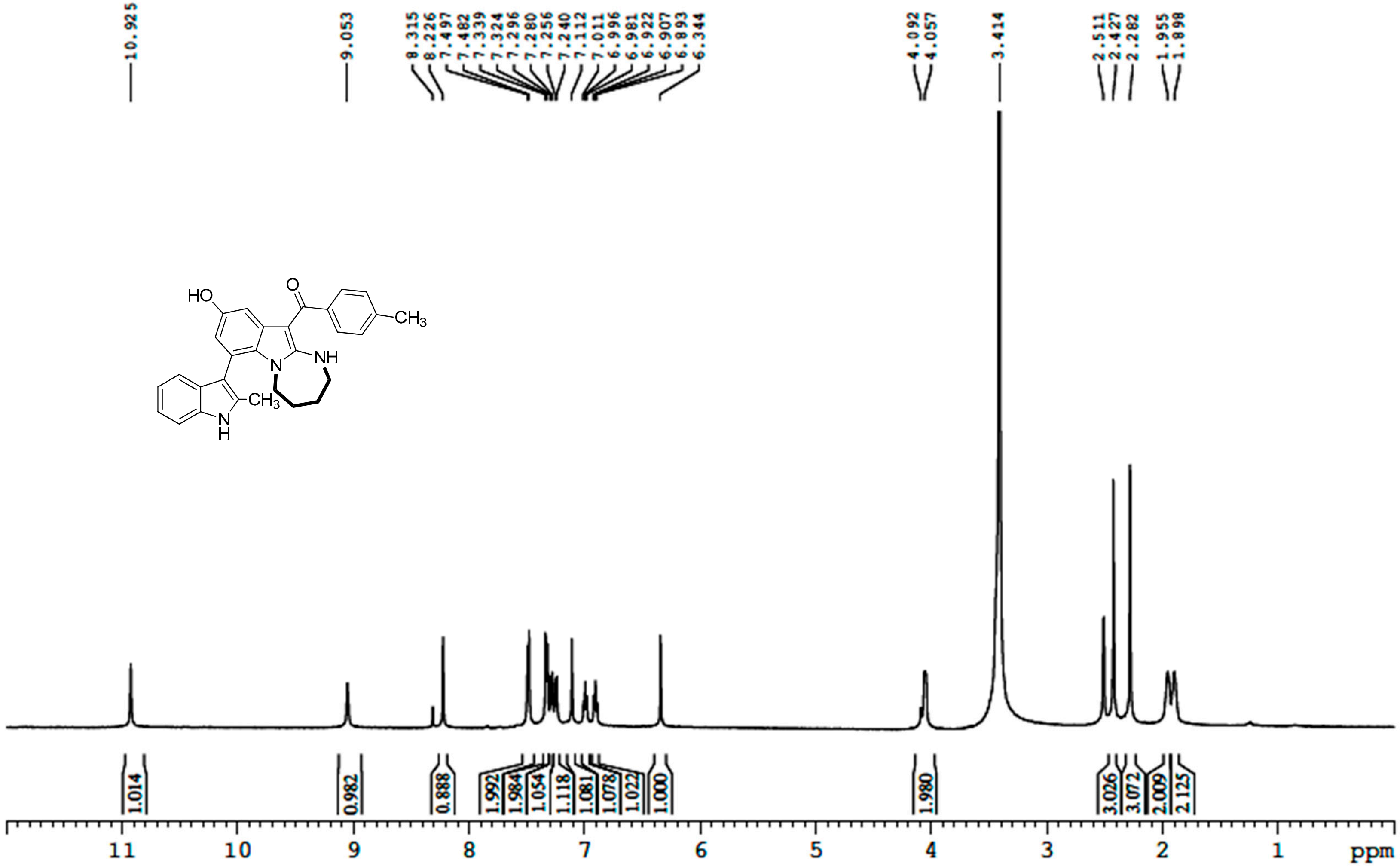

Figure S15. ^{1}H-NMR (500 MHz, DMSO-d_6) spectra of compound **3h.**

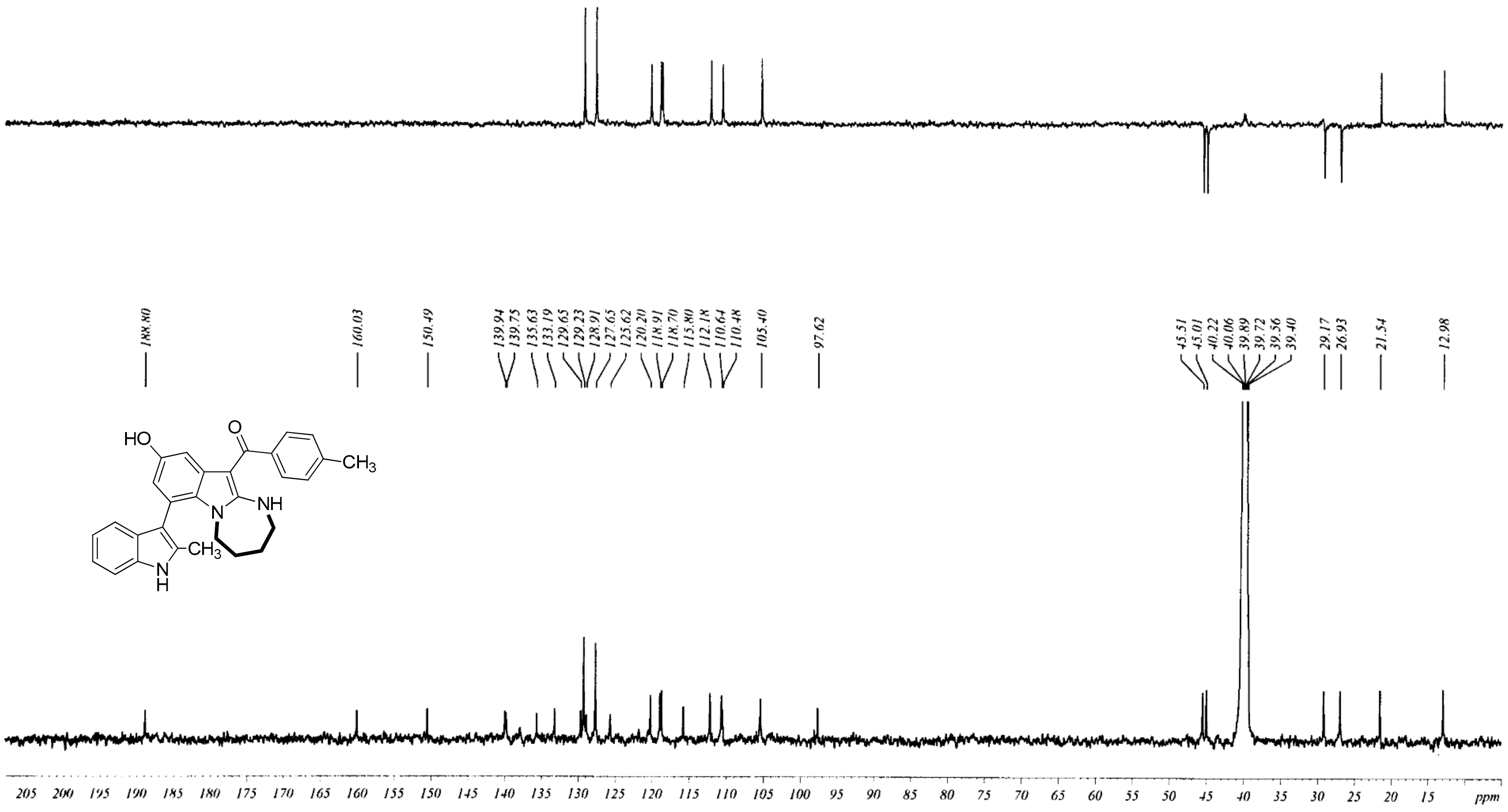

Figure S16. ^{13}C-NMR (125 MHz, DMSO-*d*$_6$) spectra of compound **3h.**

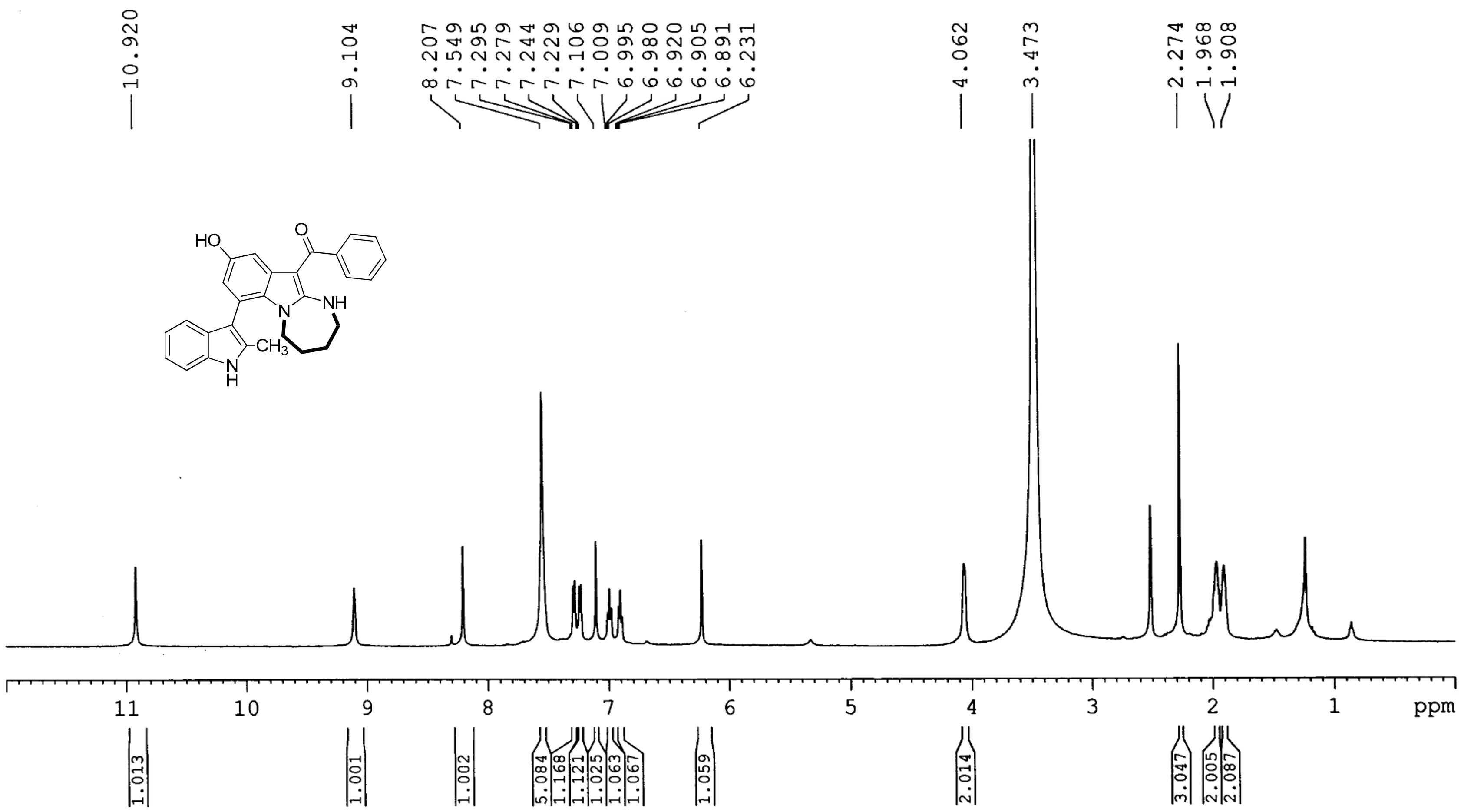

Figure S17. ^{1}H-NMR (500 MHz, DMSO-d_6) spectra of compound **3i.**

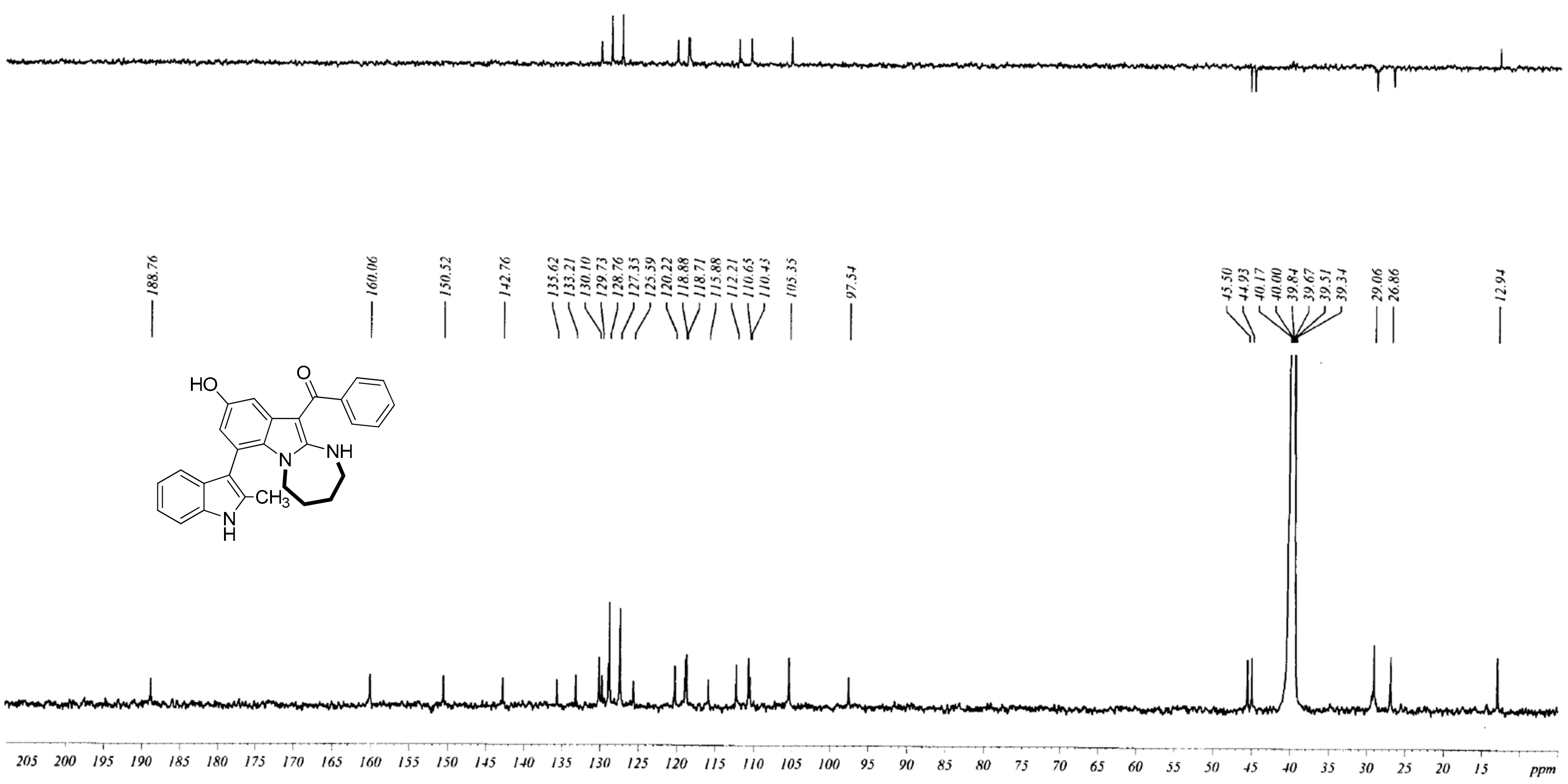

Figure S18. ^{13}C-NMR (125 MHz, DMSO-d_6) spectra of compound **3i.**

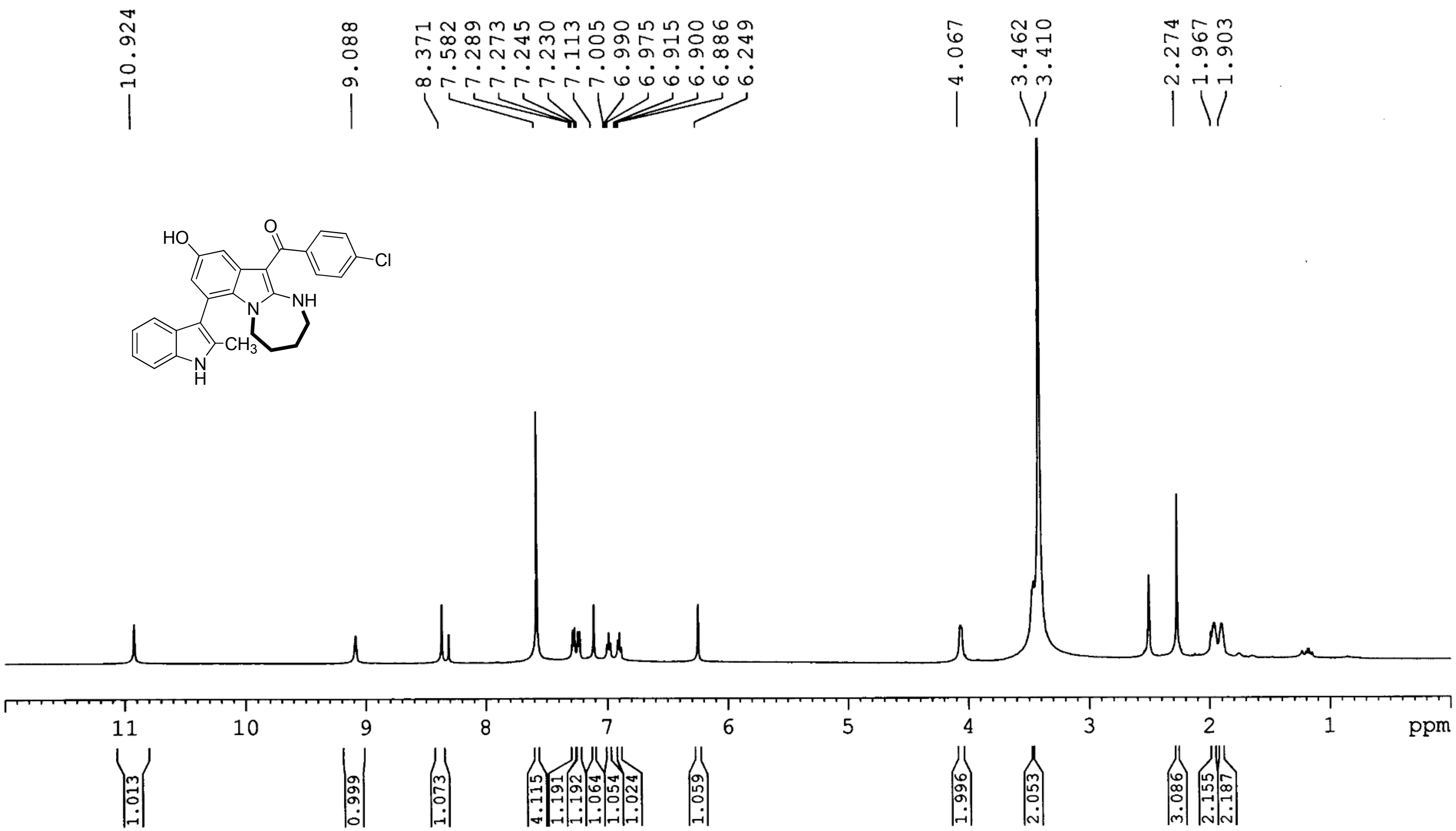

Figure S19. ^{1}H-NMR (500 MHz, DMSO-d_6) spectra of compound **3j**.

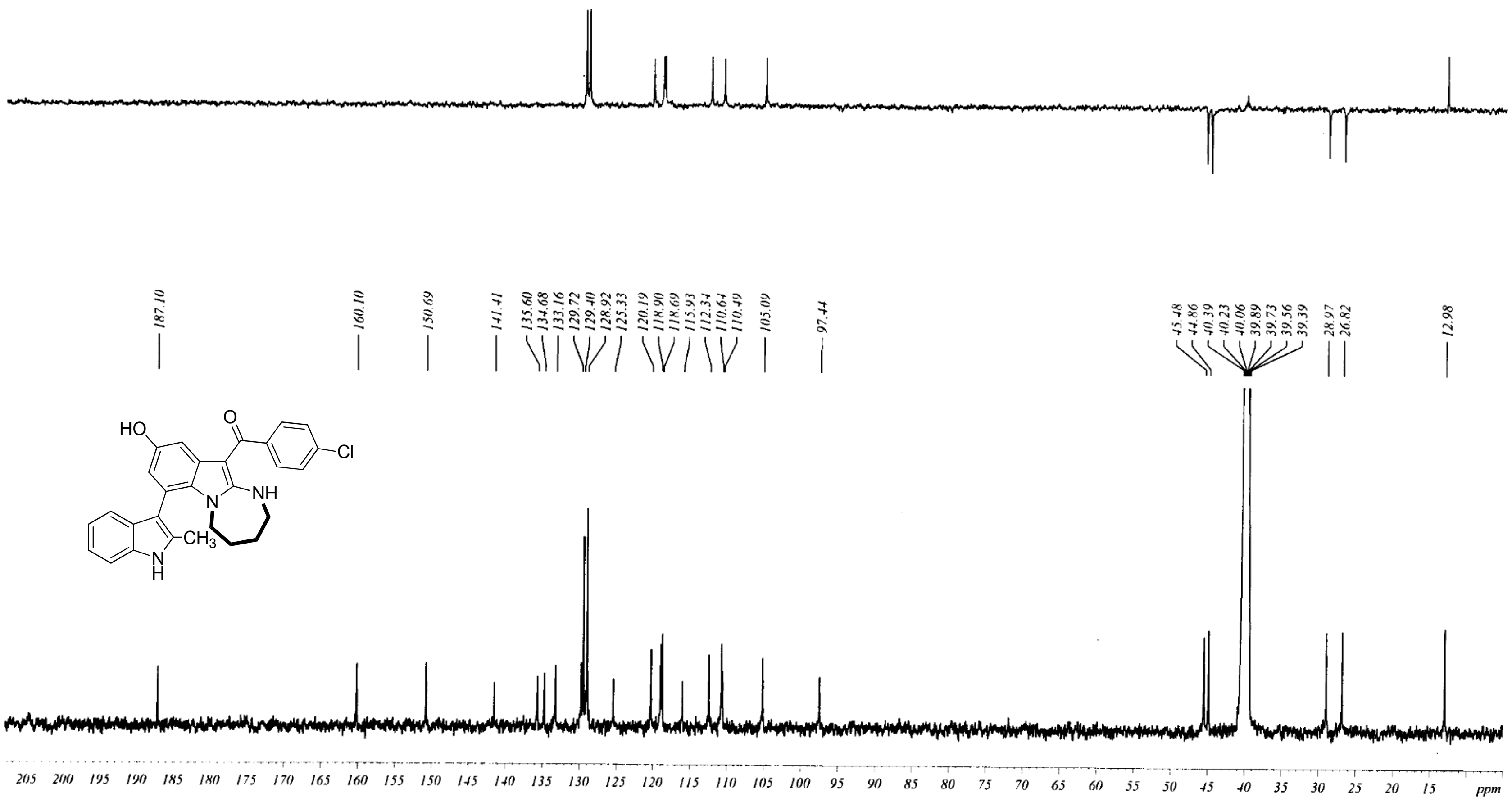

Figure S20. ^{13}C-NMR (125 MHz, DMSO-*d*$_6$) spectra of compound **3j.**

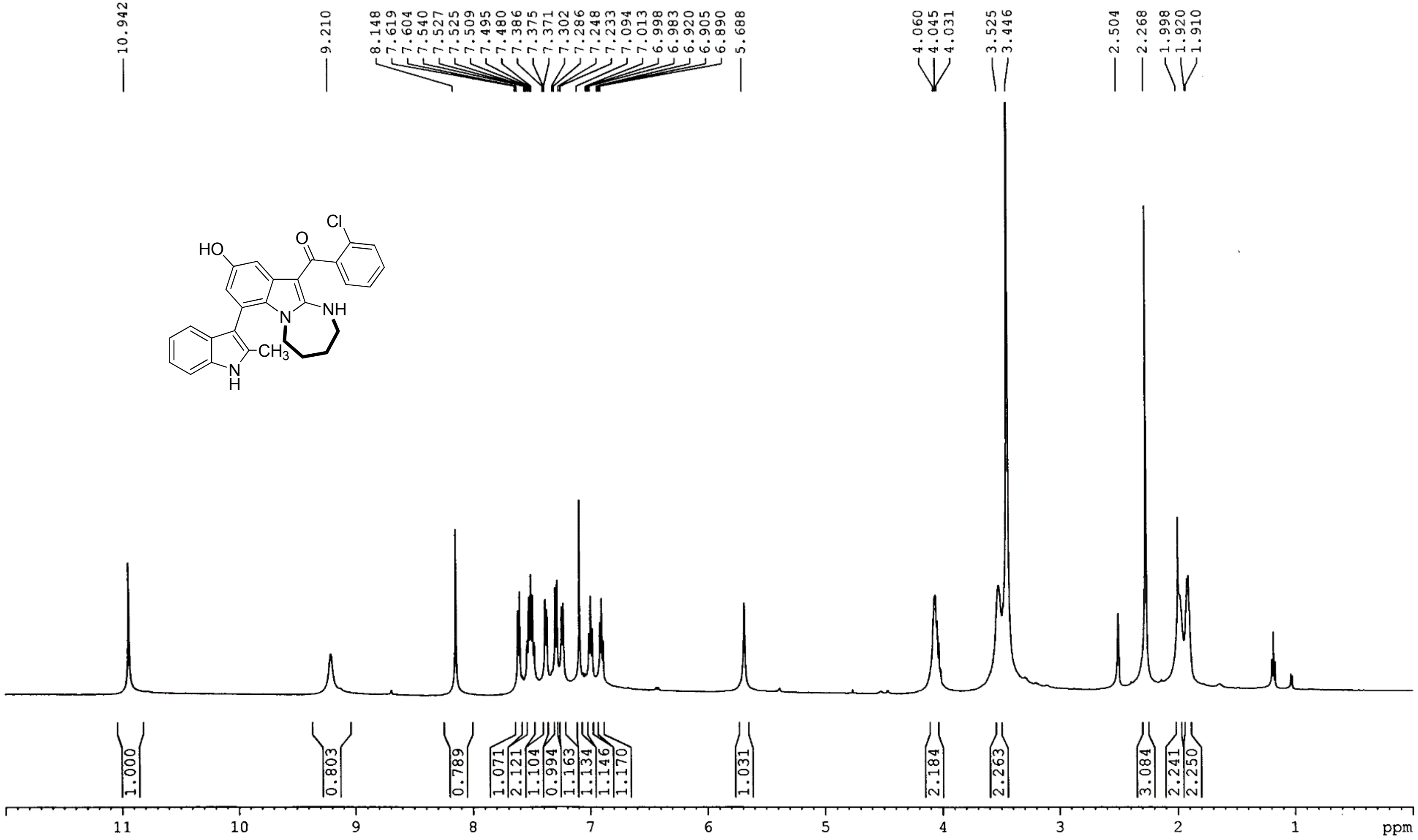

Figure S21. ^{1}H-NMR (500 MHz, DMSO-d_6) spectra of compound **3k**.

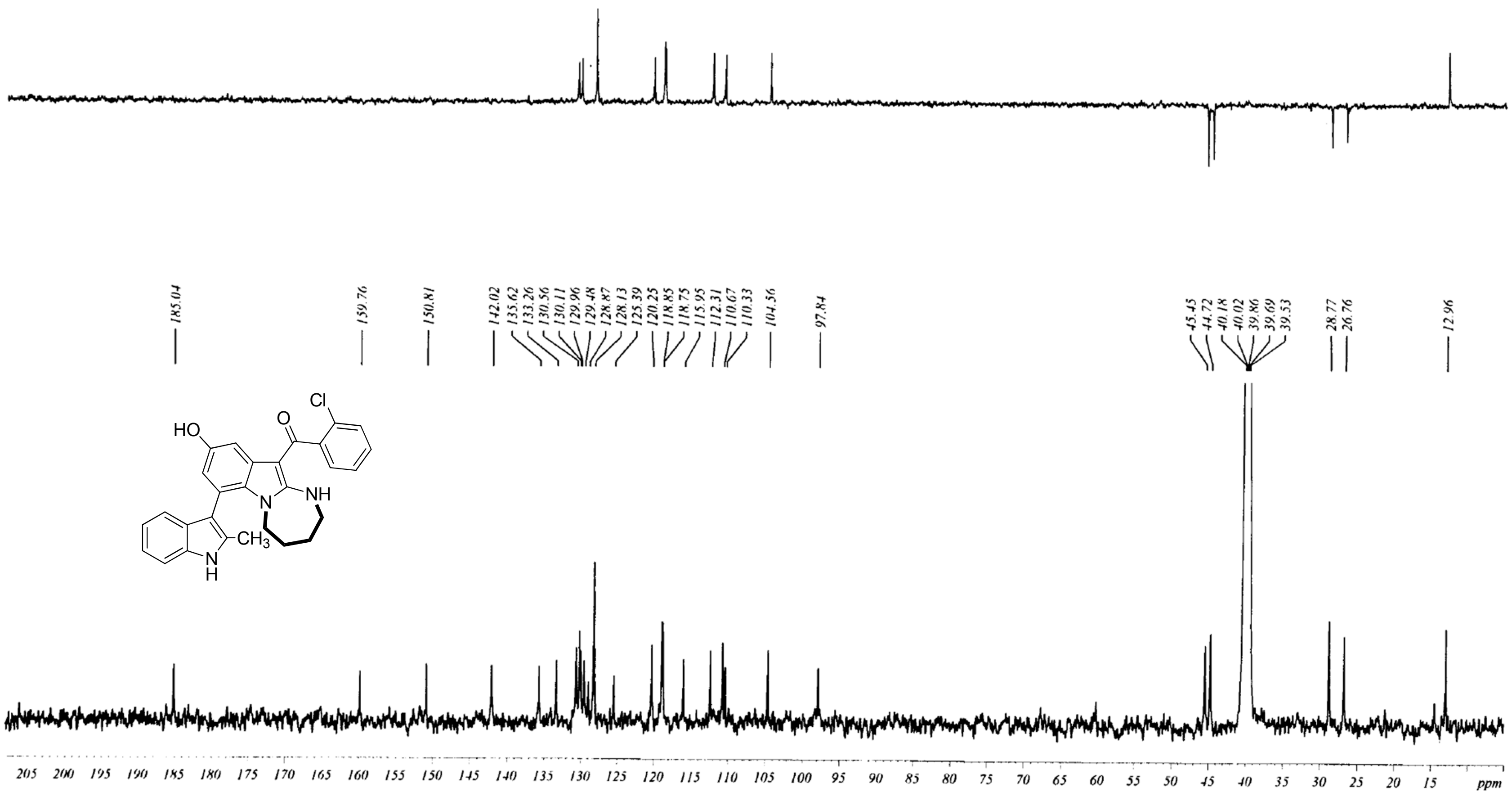

Figure S22. ^{13}C-NMR (125 MHz, DMSO-d_6) spectra of compound **3k.**

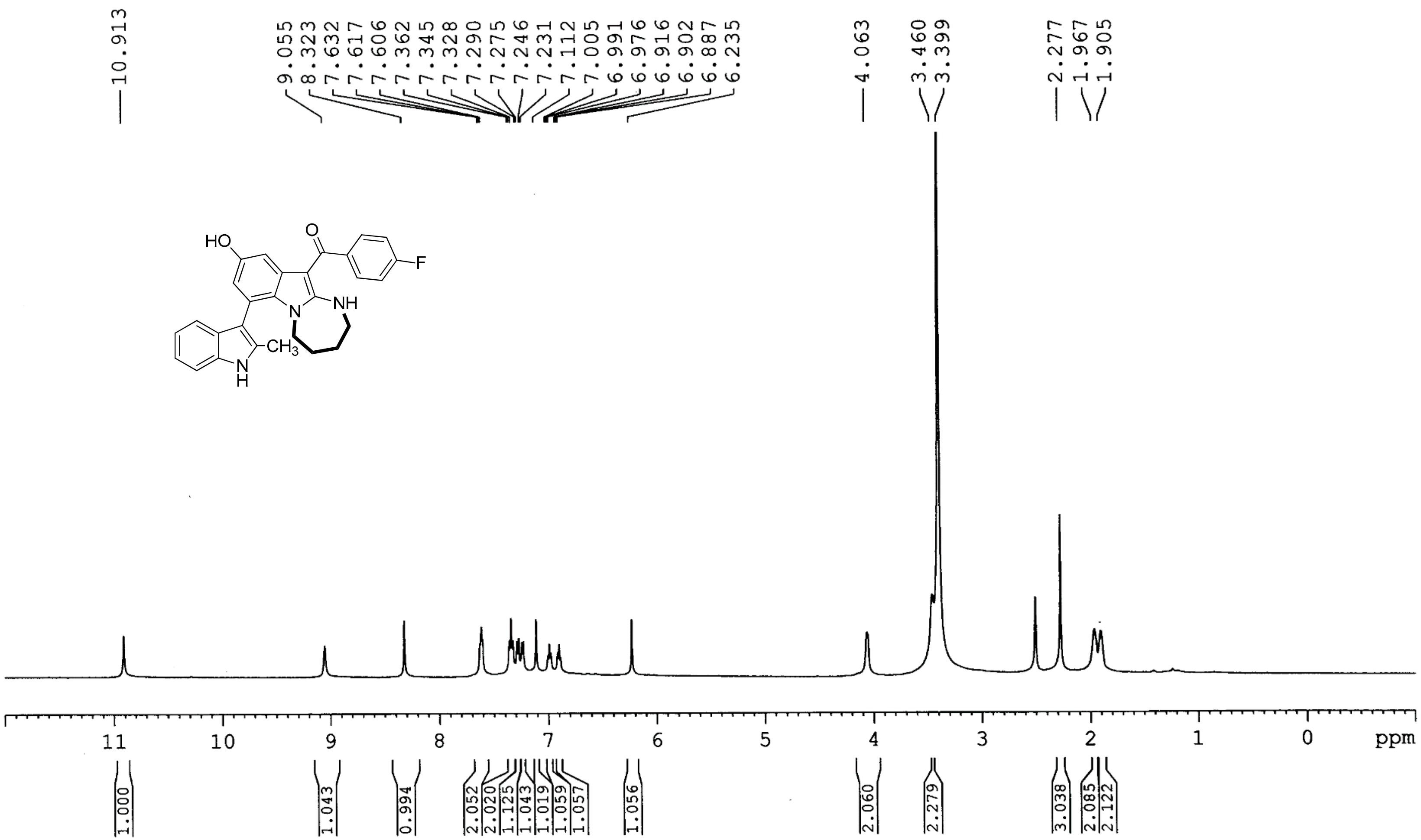

Figure S23. ^{1}H-NMR (500 MHz, DMSO-d_6) spectra of compound **3l**.

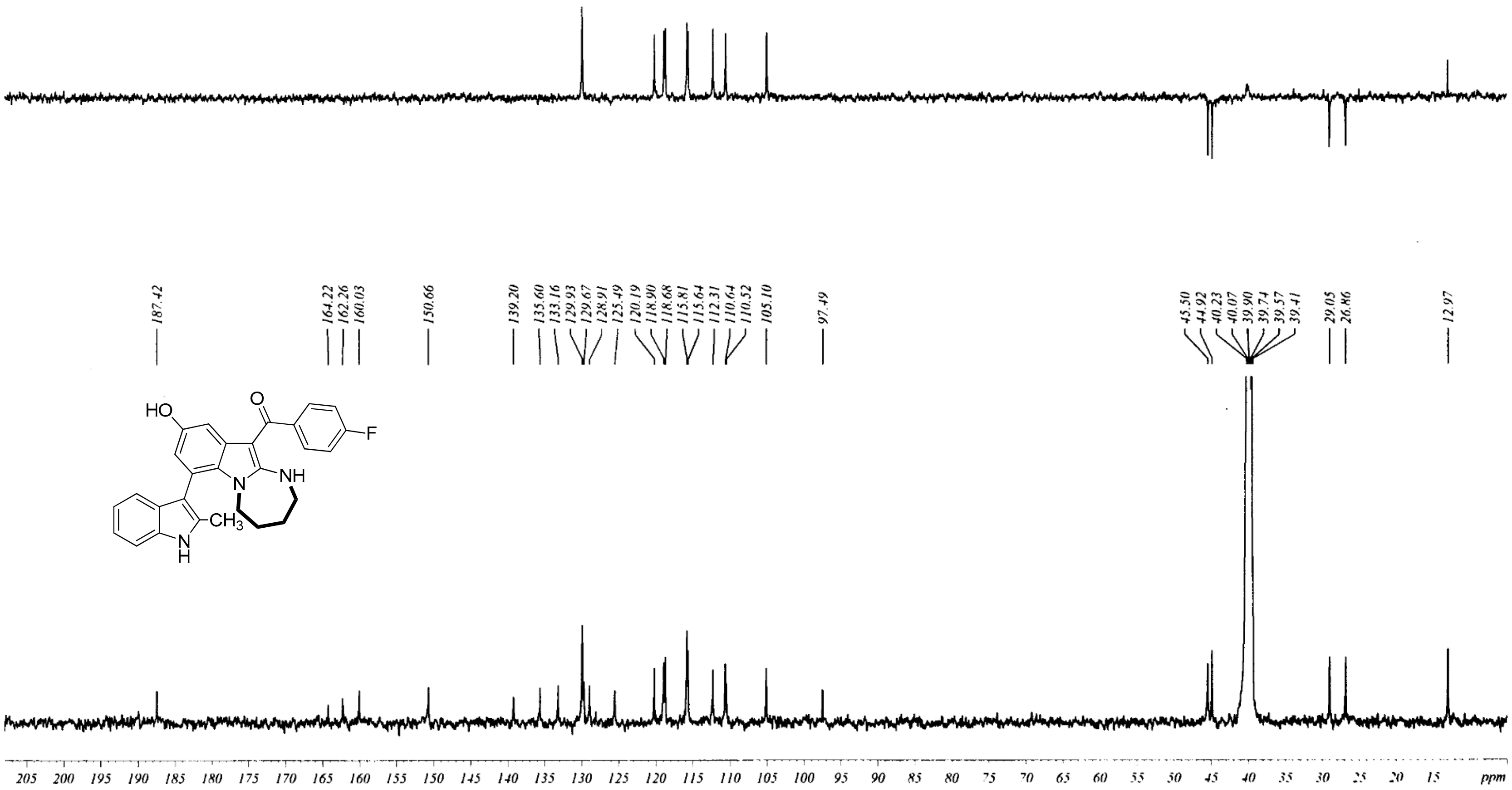

Figure S24. ^{13}C-NMR (125 MHz, DMSO-d_6) spectra of compound **3l.**

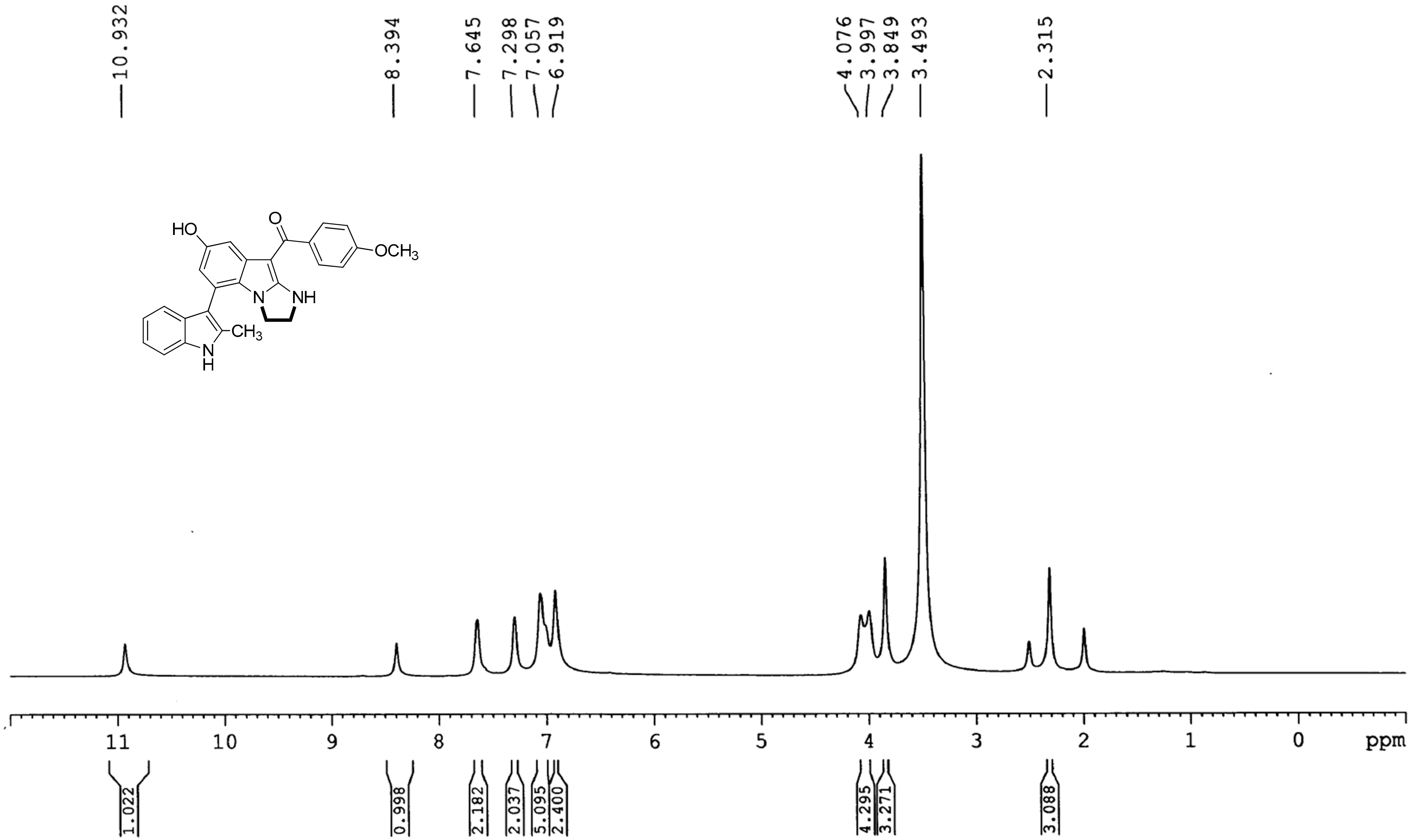

Figure S25. ^{1}H-NMR (500 MHz, DMSO-d_6) spectra of compound **3m**.

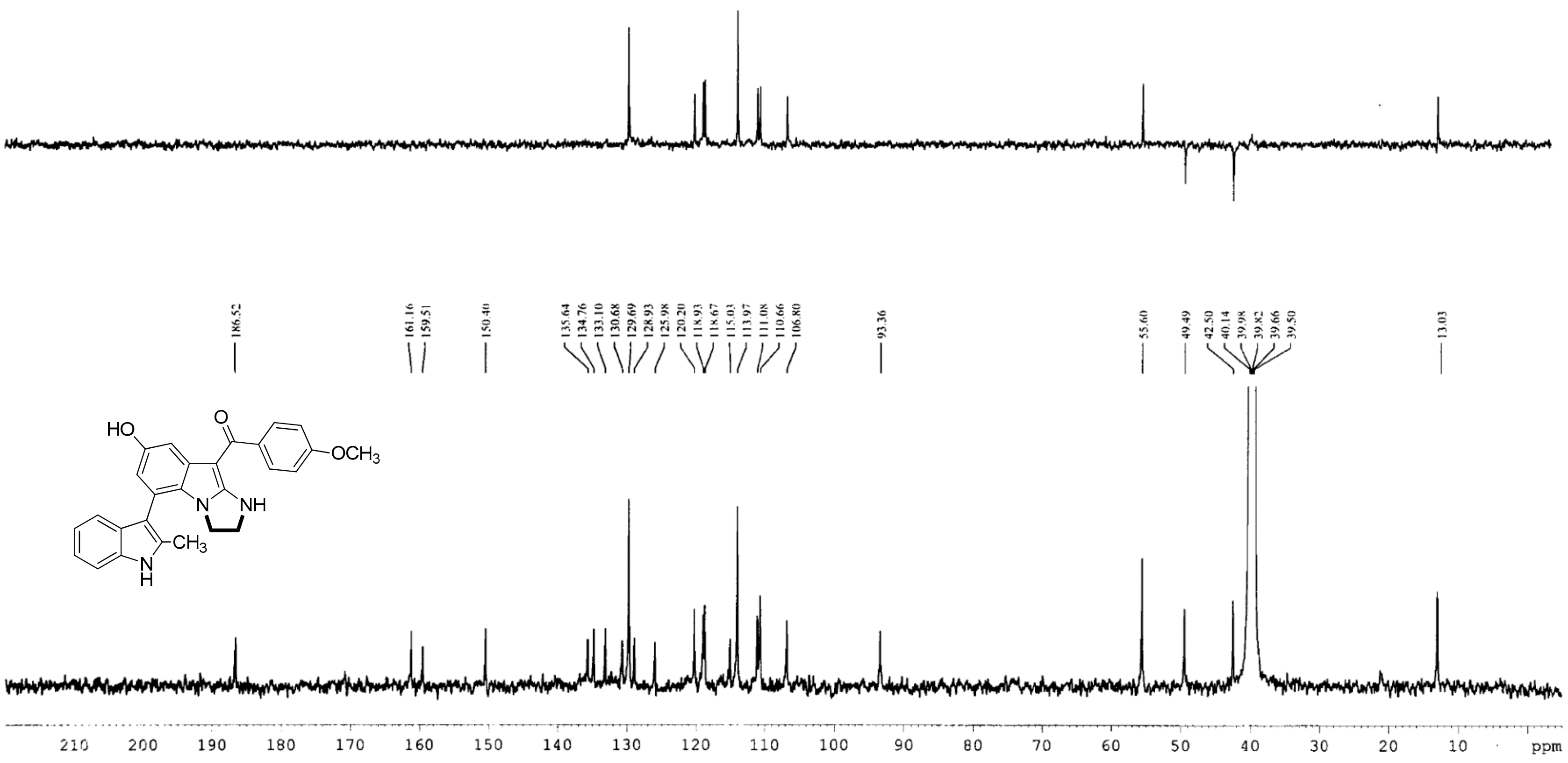

Figure S26. ^{13}C-NMR (125 MHz, DMSO-d_6) spectra of compound **3m.**

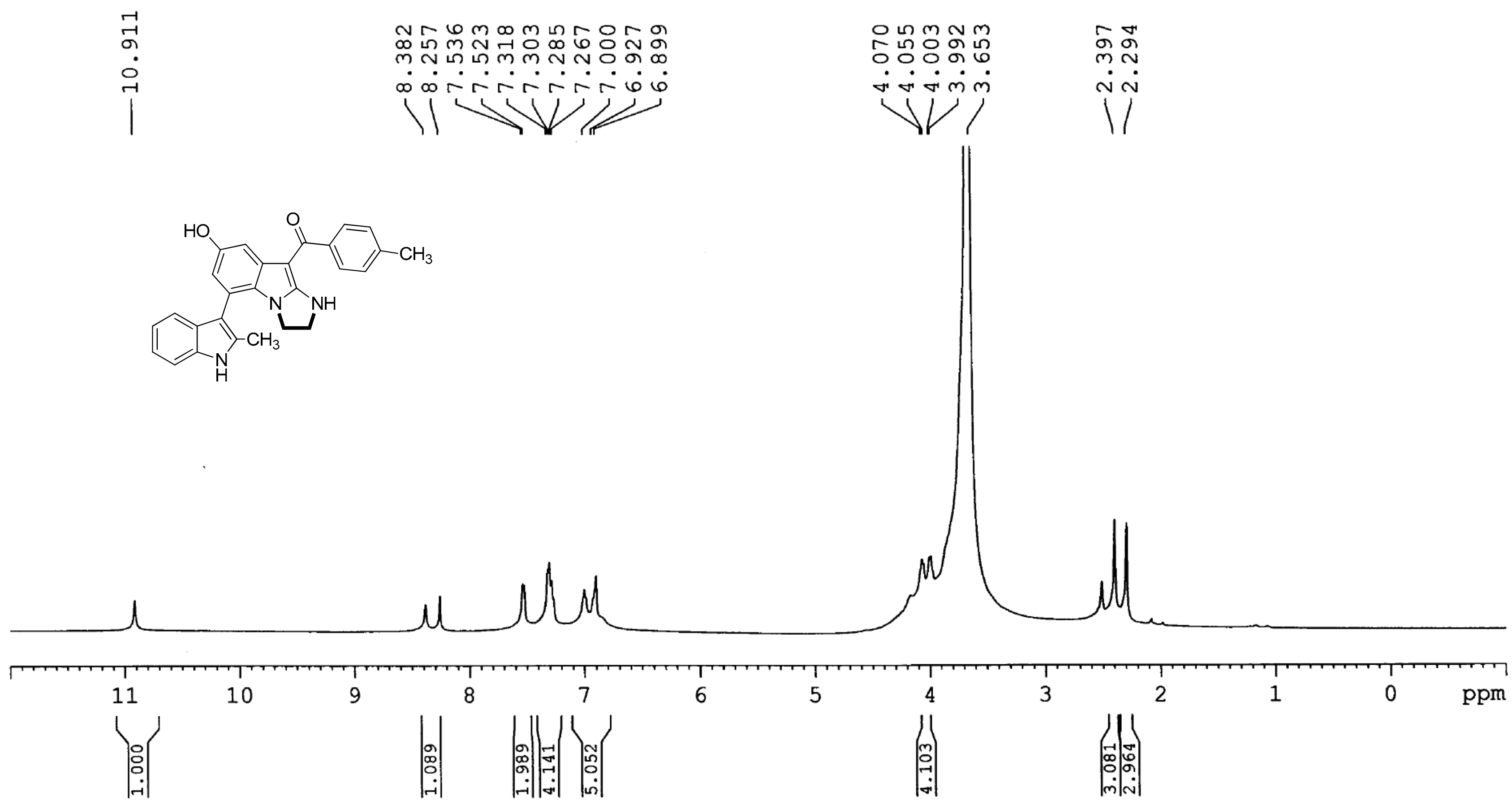

Figure S27. ^{1}H-NMR (500 MHz, DMSO-d_6) spectra of compound **3n**.

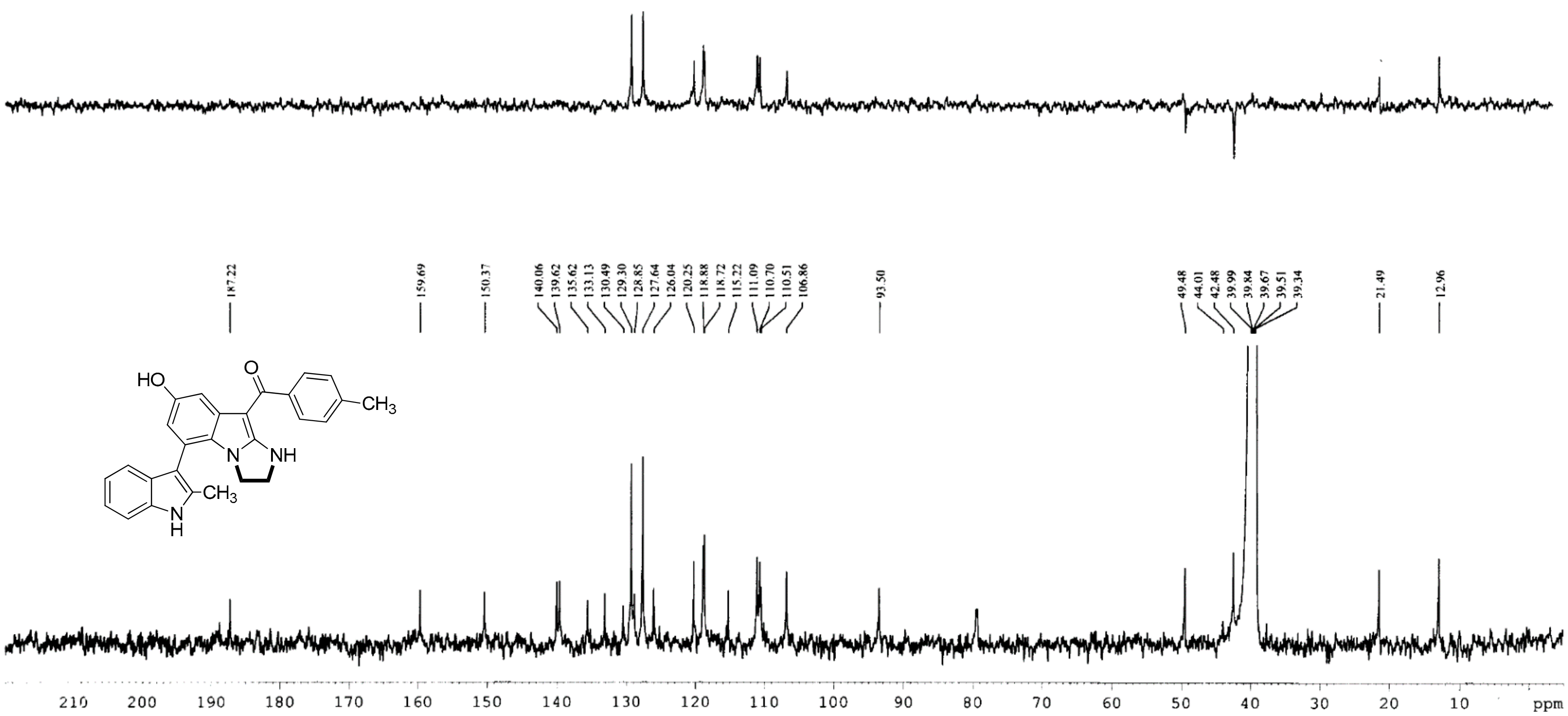

Figure S28. ^{13}C-NMR (125 MHz, DMSO-d_6) spectra of compound **3n**.

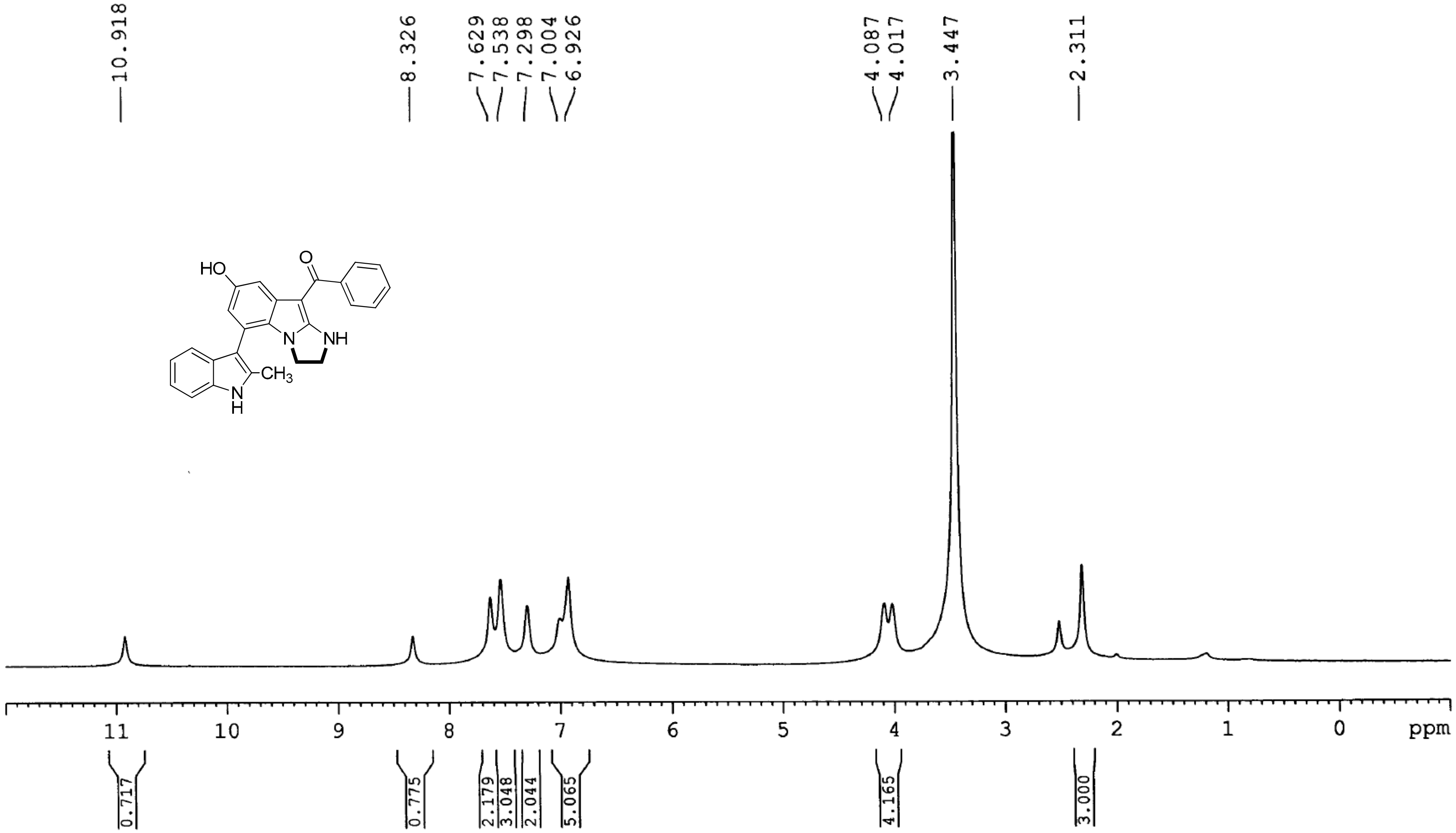

Figure S29. ^{1}H-NMR (500 MHz, DMSO-*d*6) spectra of compound **3o**.

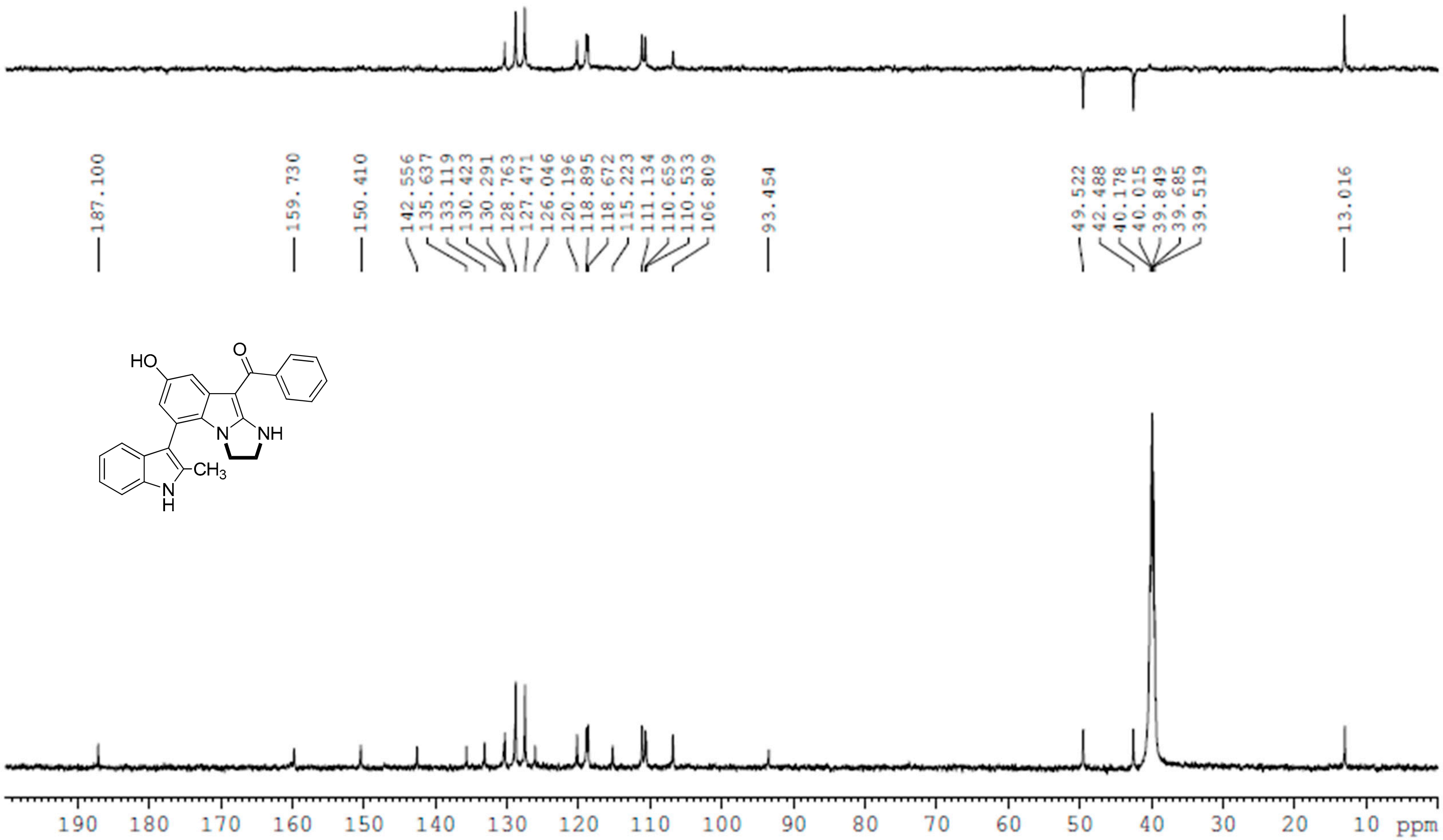

Figure S30. ^{13}C-NMR (125 MHz, DMSO-d_6) spectra of compound **3o**.

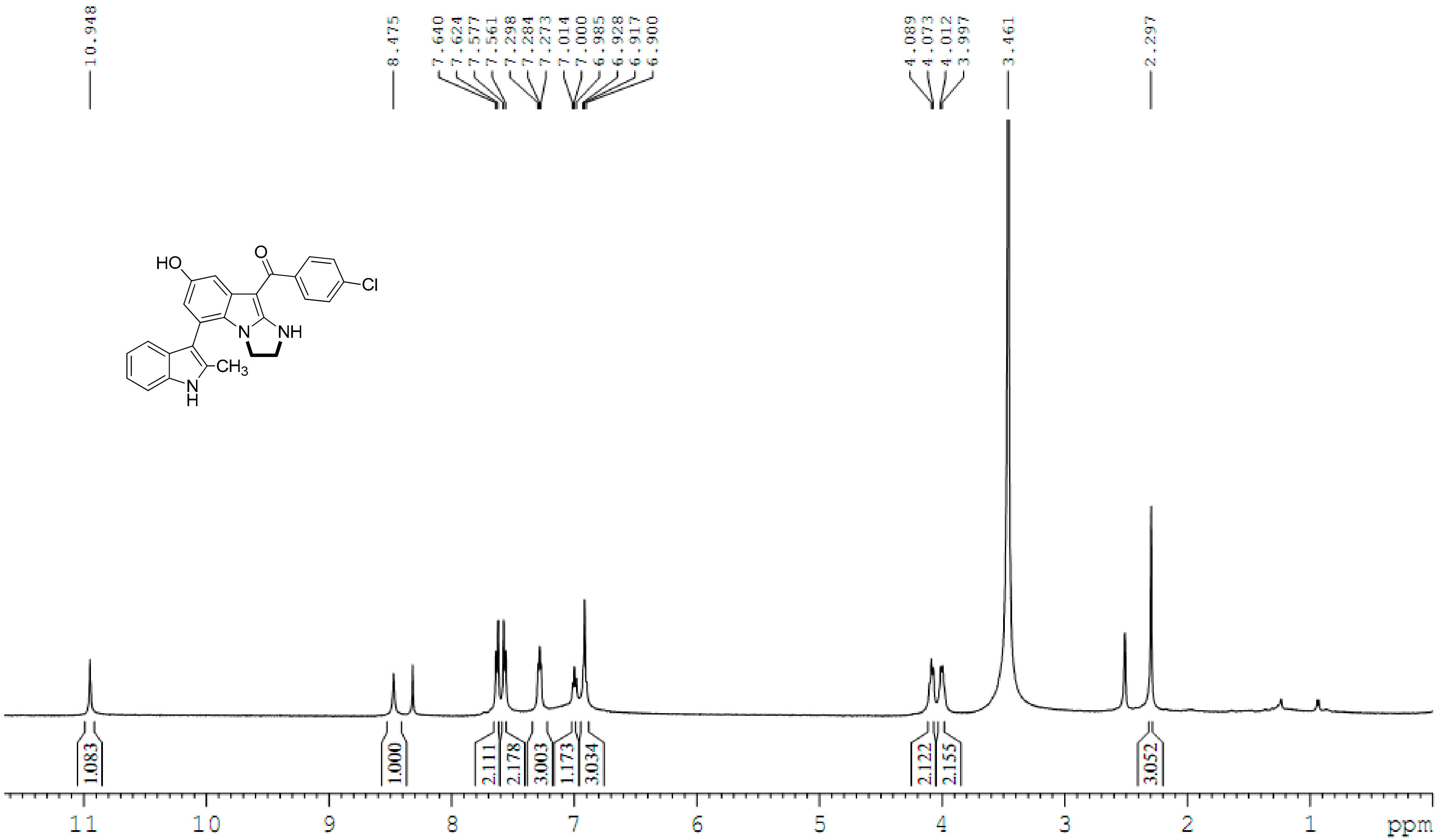

Figure S31. ^{1}H-NMR (500 MHz, DMSO-*d*6) spectra of compound **3p**.

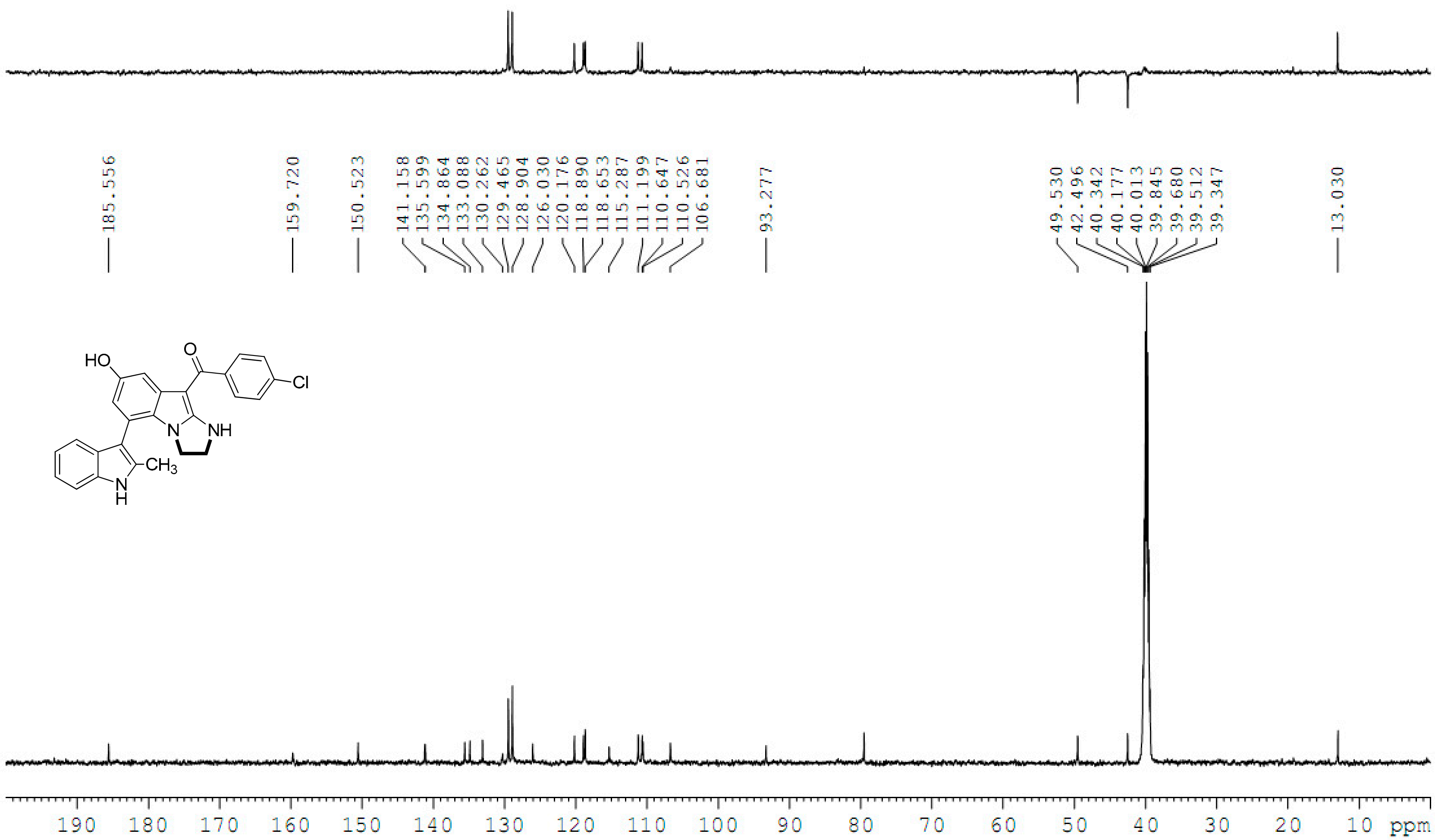

Figure S32. ^{13}C-NMR (125 MHz, DMSO-*d*$_6$) spectra of compound **3p**.

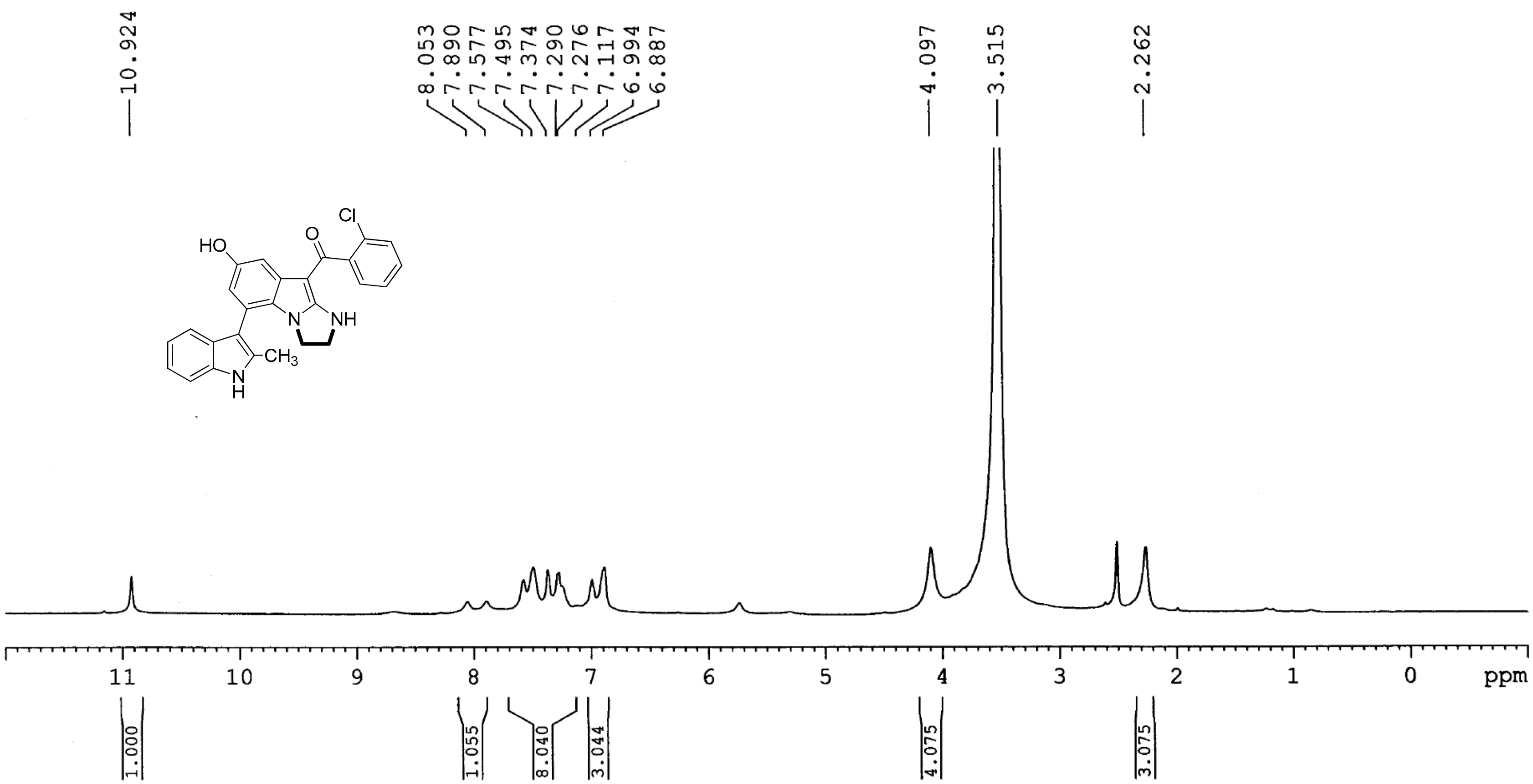

Figure S33. ^{1}H-NMR (500 MHz, DMSO-d_6) spectra of compound **3q**.

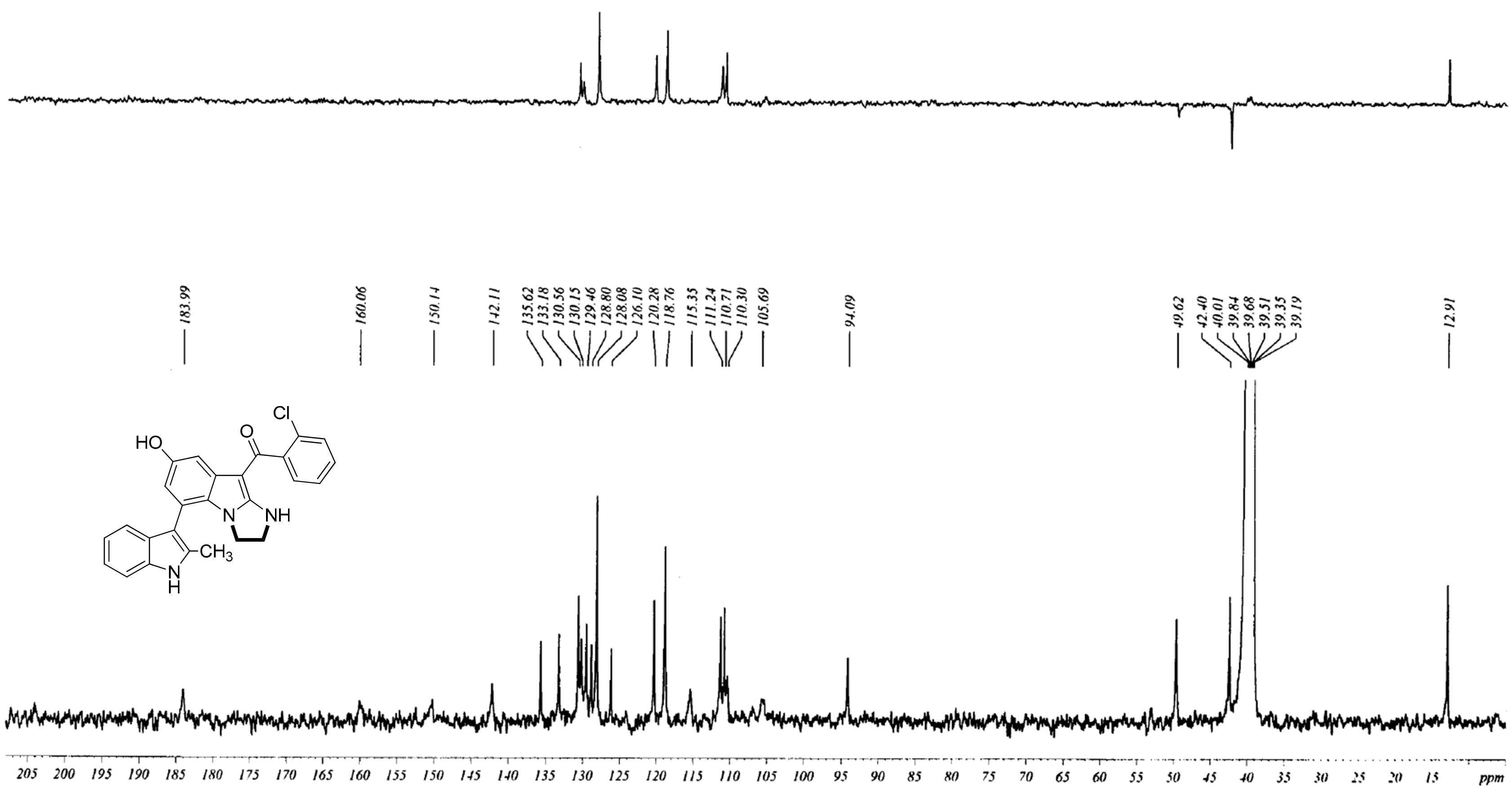

Figure S34. ^{13}C-NMR (125 MHz, DMSO-d_6) spectra of compound **3q**.

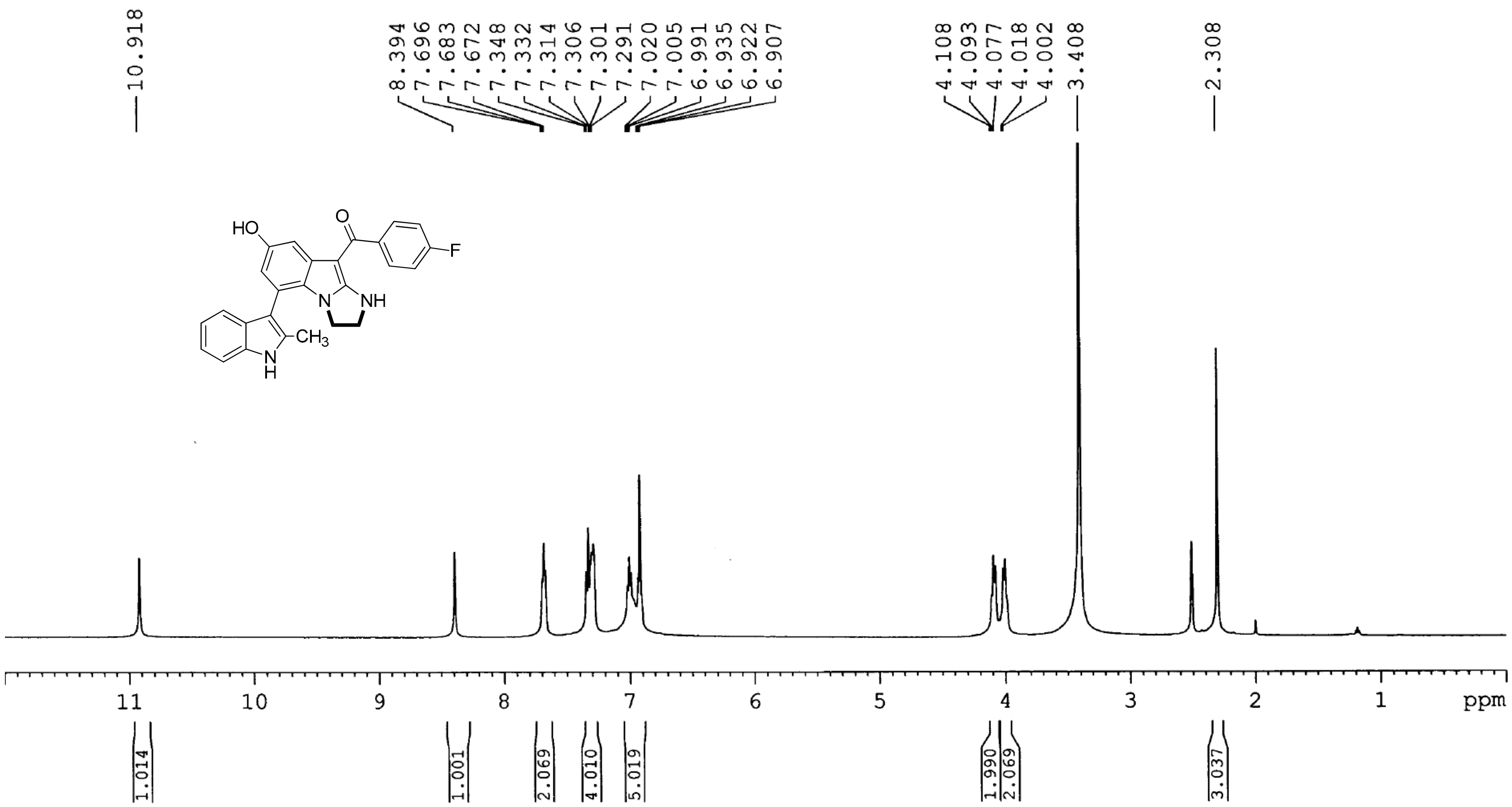

Figure S35. ^{1}H-NMR (500 MHz, DMSO-d_6) spectra of compound **3r**.

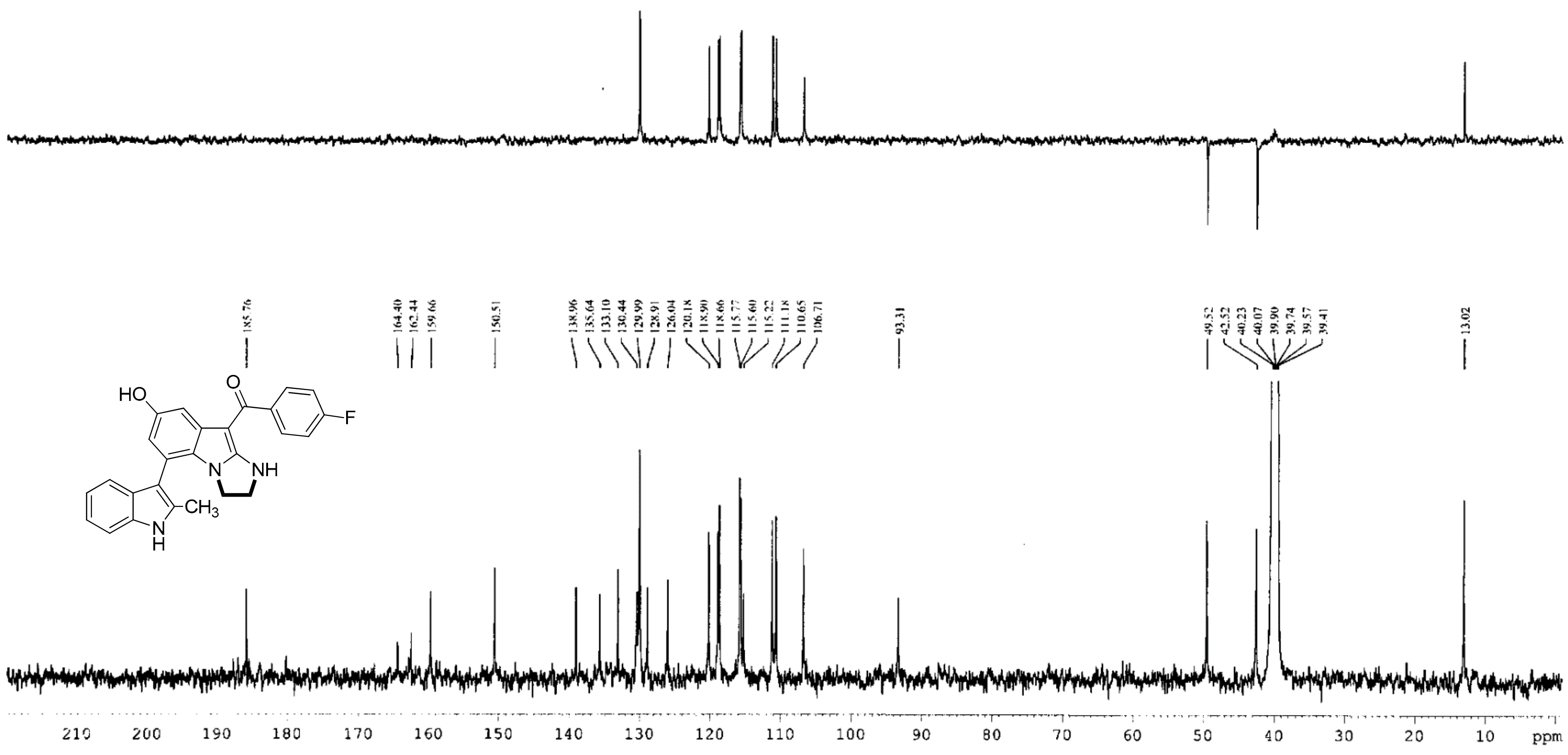

Figure S36. ^{13}C-NMR (125 MHz, DMSO-*d*6) spectra of compound **3r**.

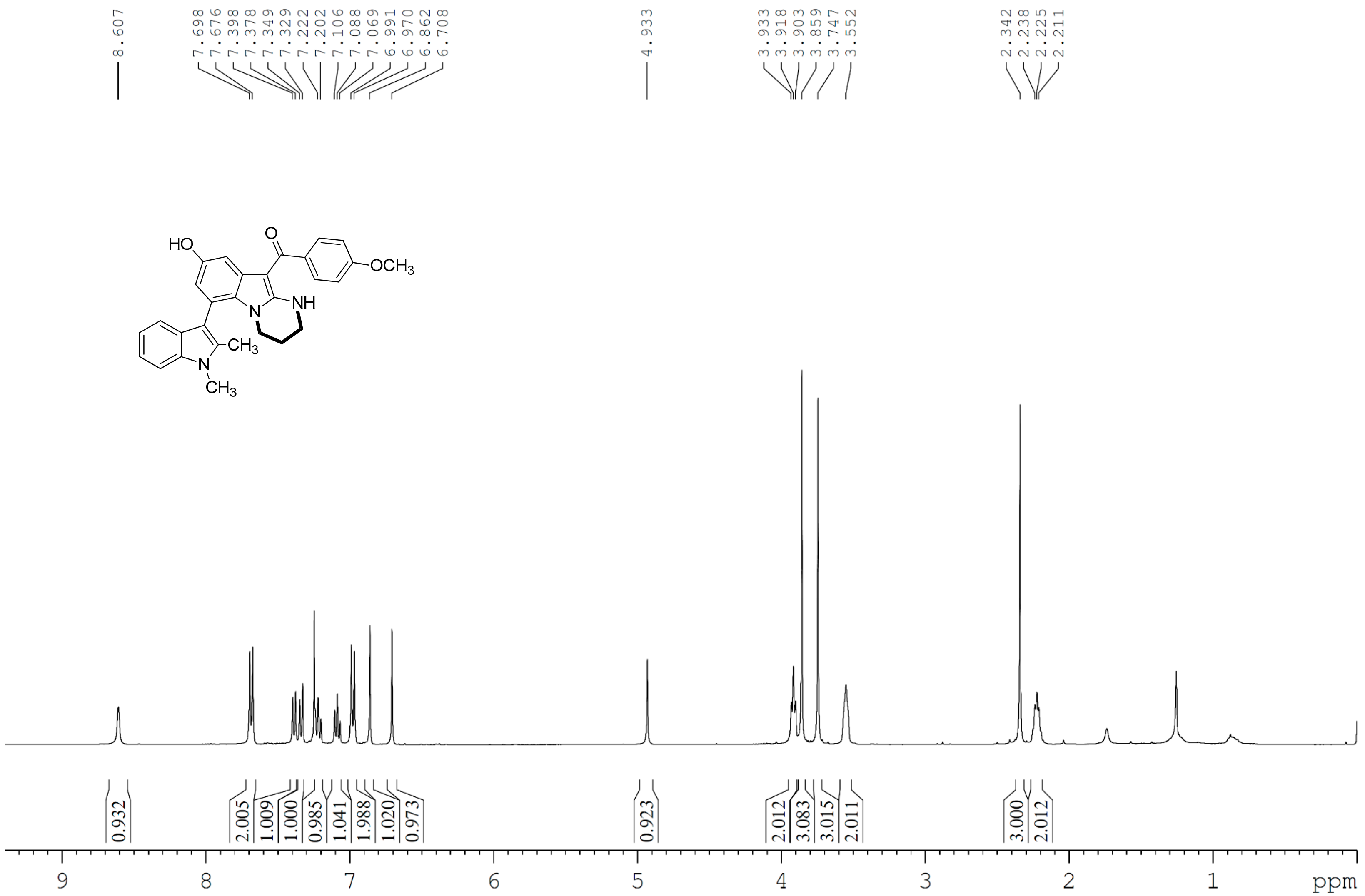

Figure S37. ^{1}H-NMR (400 MHz, $CDCl_3$) spectra of compound **3s.**

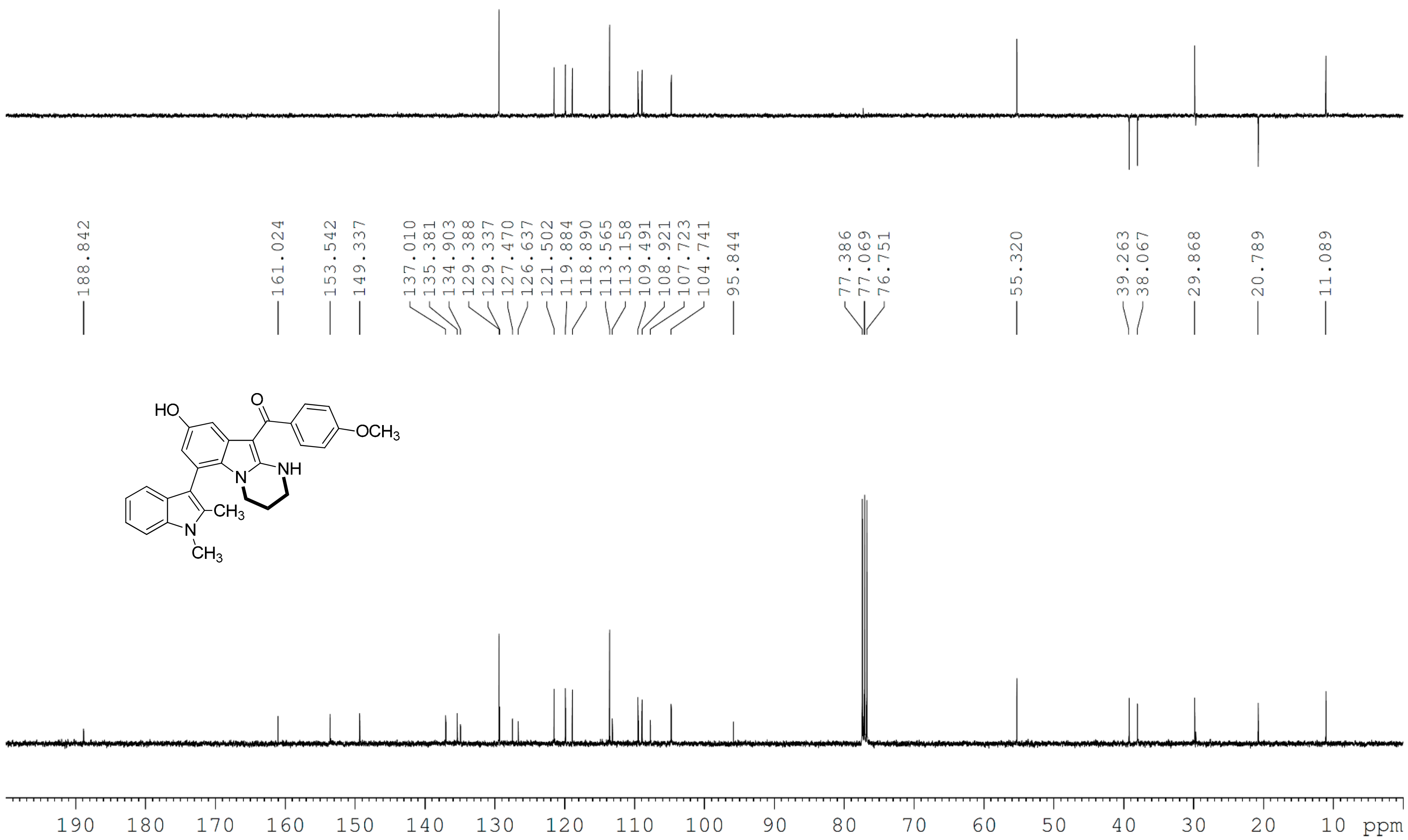

Figure S38. ^{13}C-NMR (100 MHz, $CDCl_3$) spectra of compound **3s**.

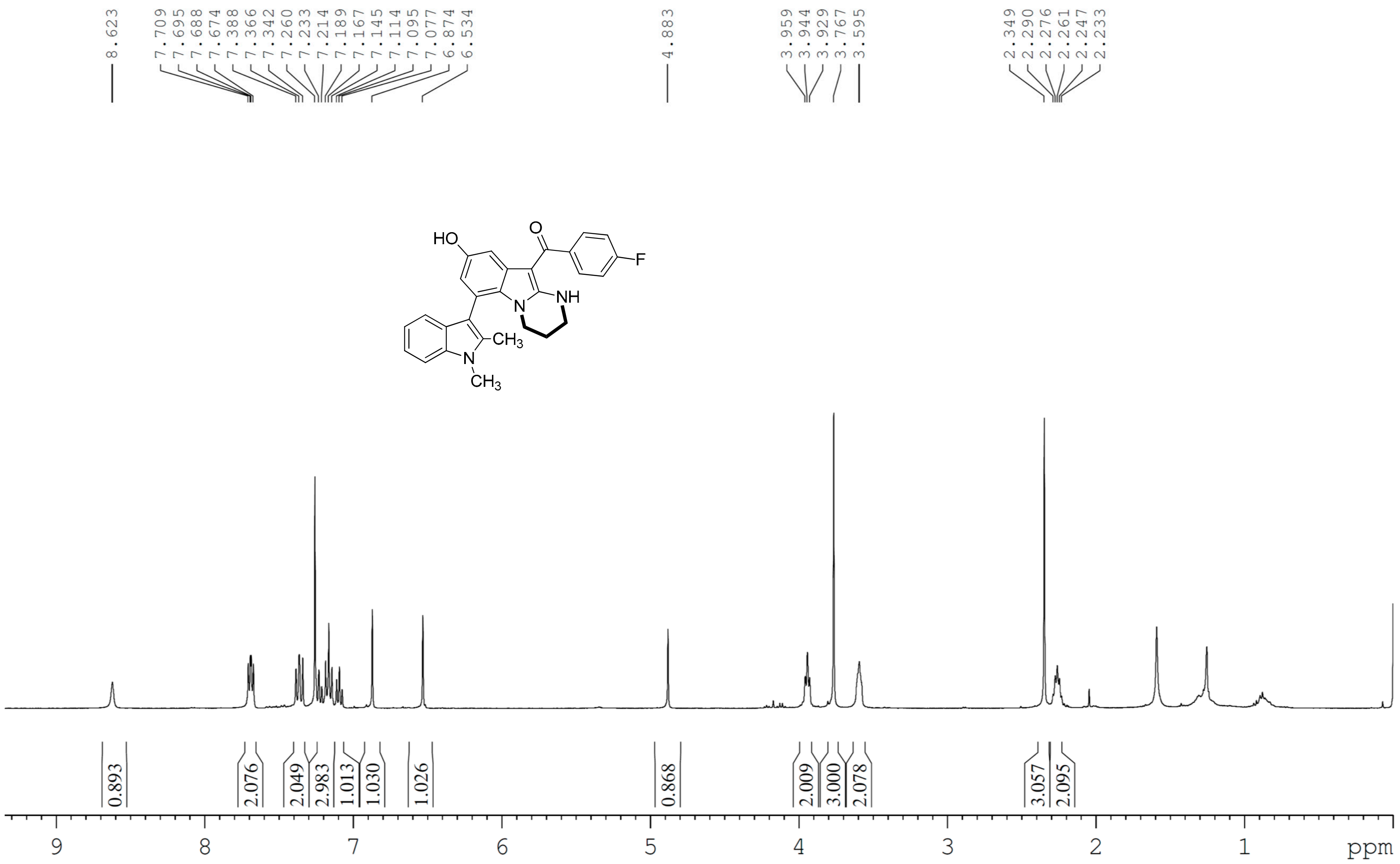

Figure S39. ^{1}H-NMR (400 MHz, $CDCl_3$) spectra of compound **3t.**

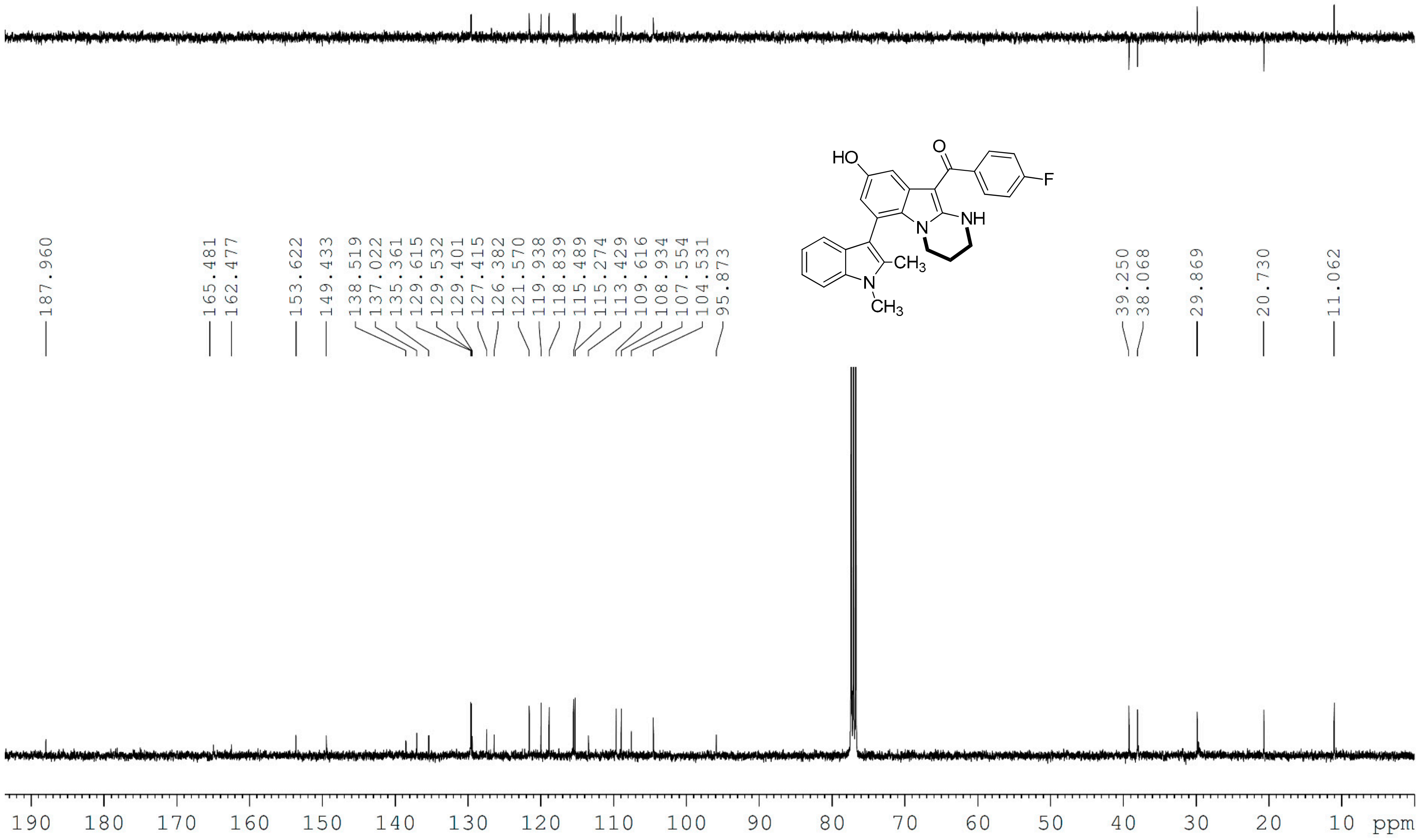

Figure S40. ^{13}C-NMR (100 MHz, $CDCl_3$) spectra of compound **3t**.

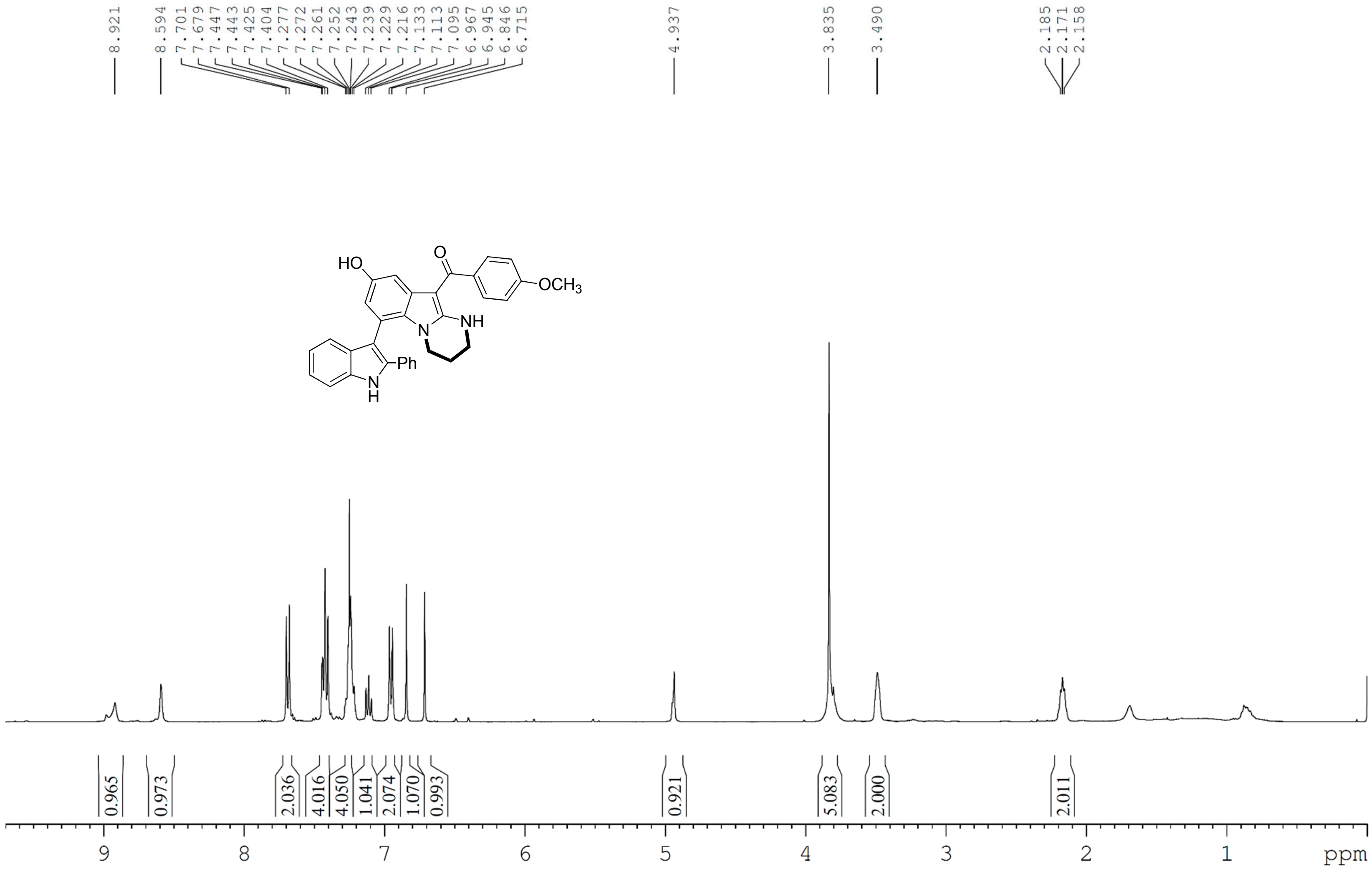

Figure S41. ^{1}H-NMR (400 MHz, $CDCl_3$) spectra of compound **3u.**

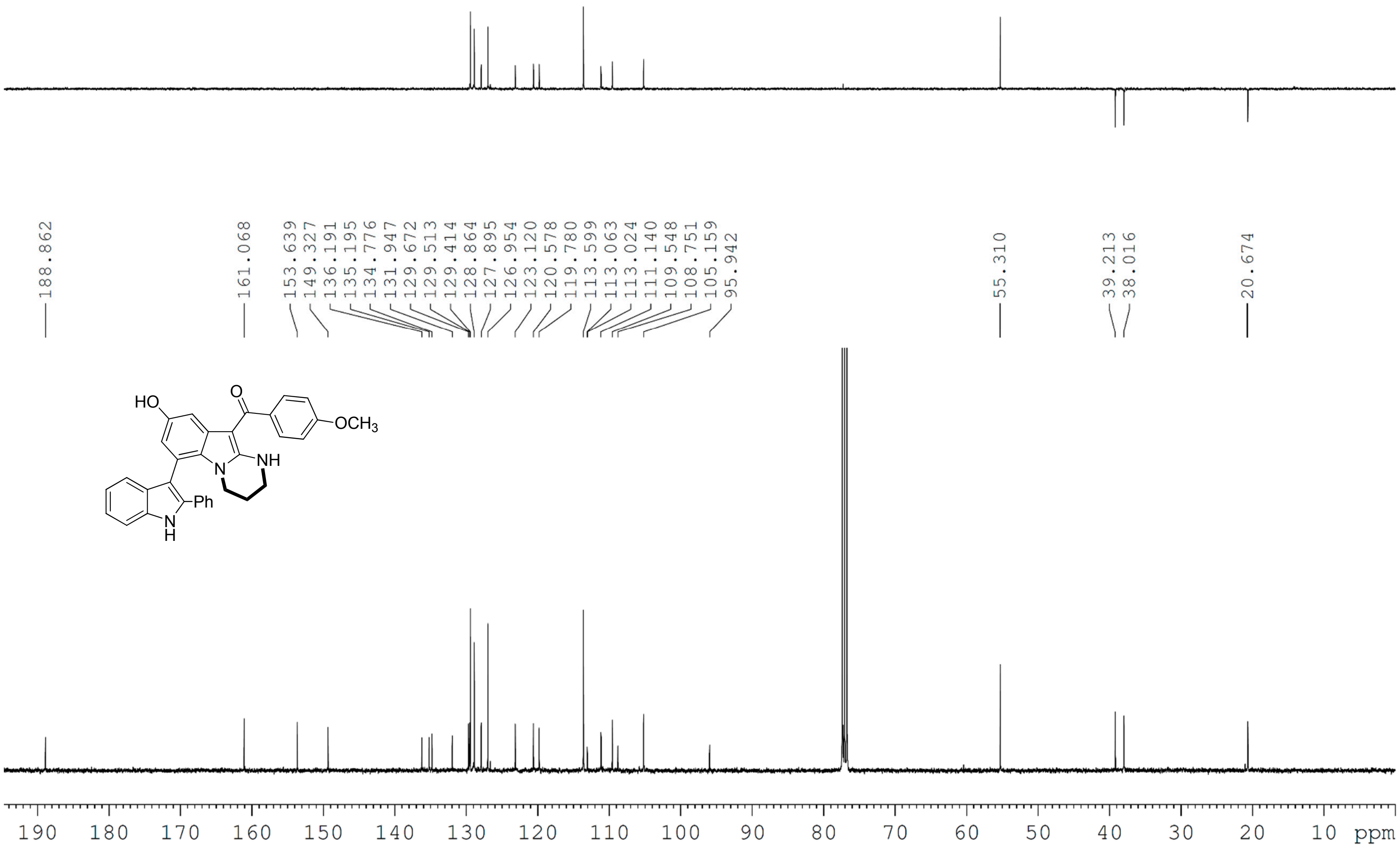

Figure S42. ^{13}C-NMR (100 MHz, $CDCl_3$) spectra of compound **3u**.

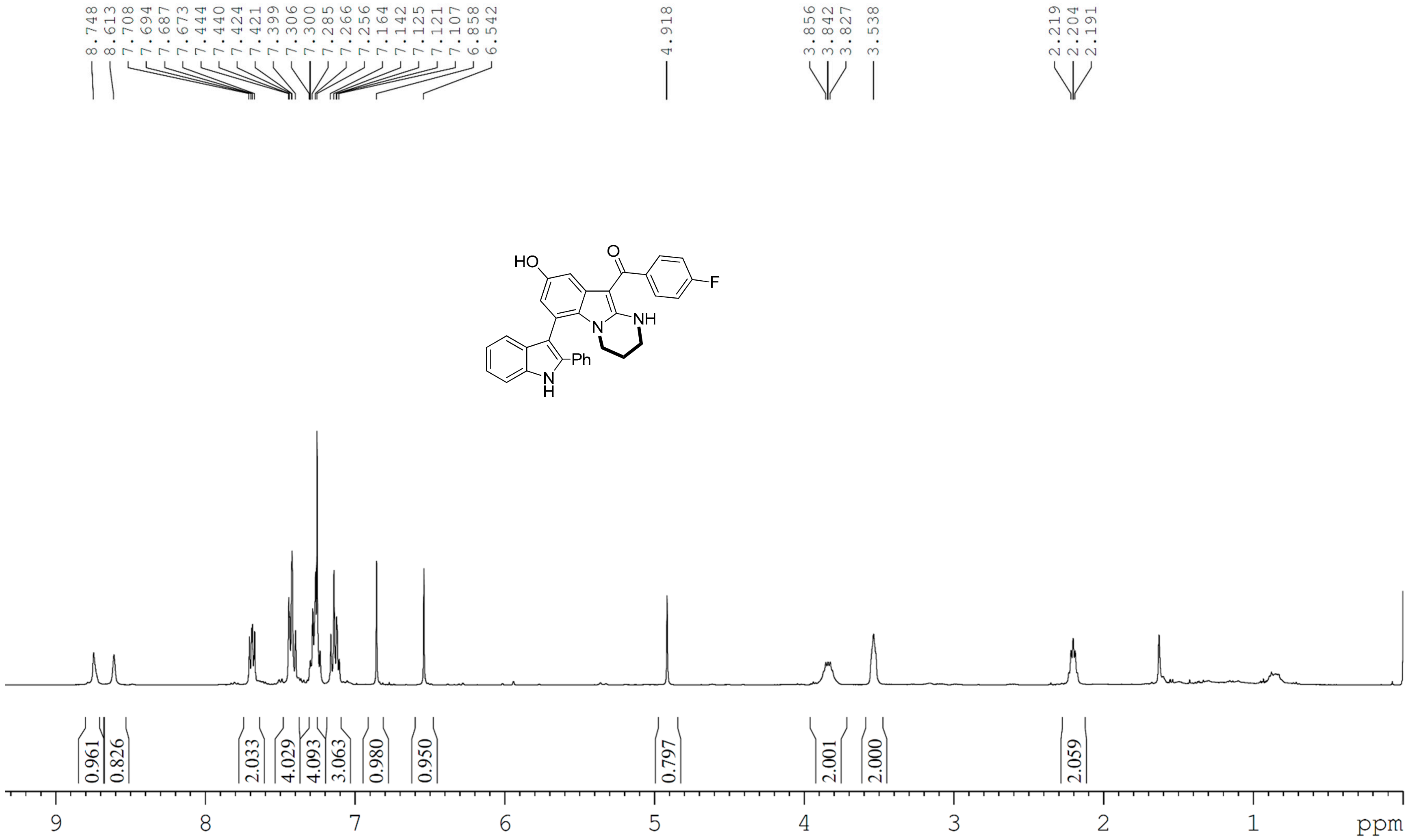

Figure S43. ^{1}H-NMR (400 MHz, $CDCl_3$) spectra of compound **3v.**

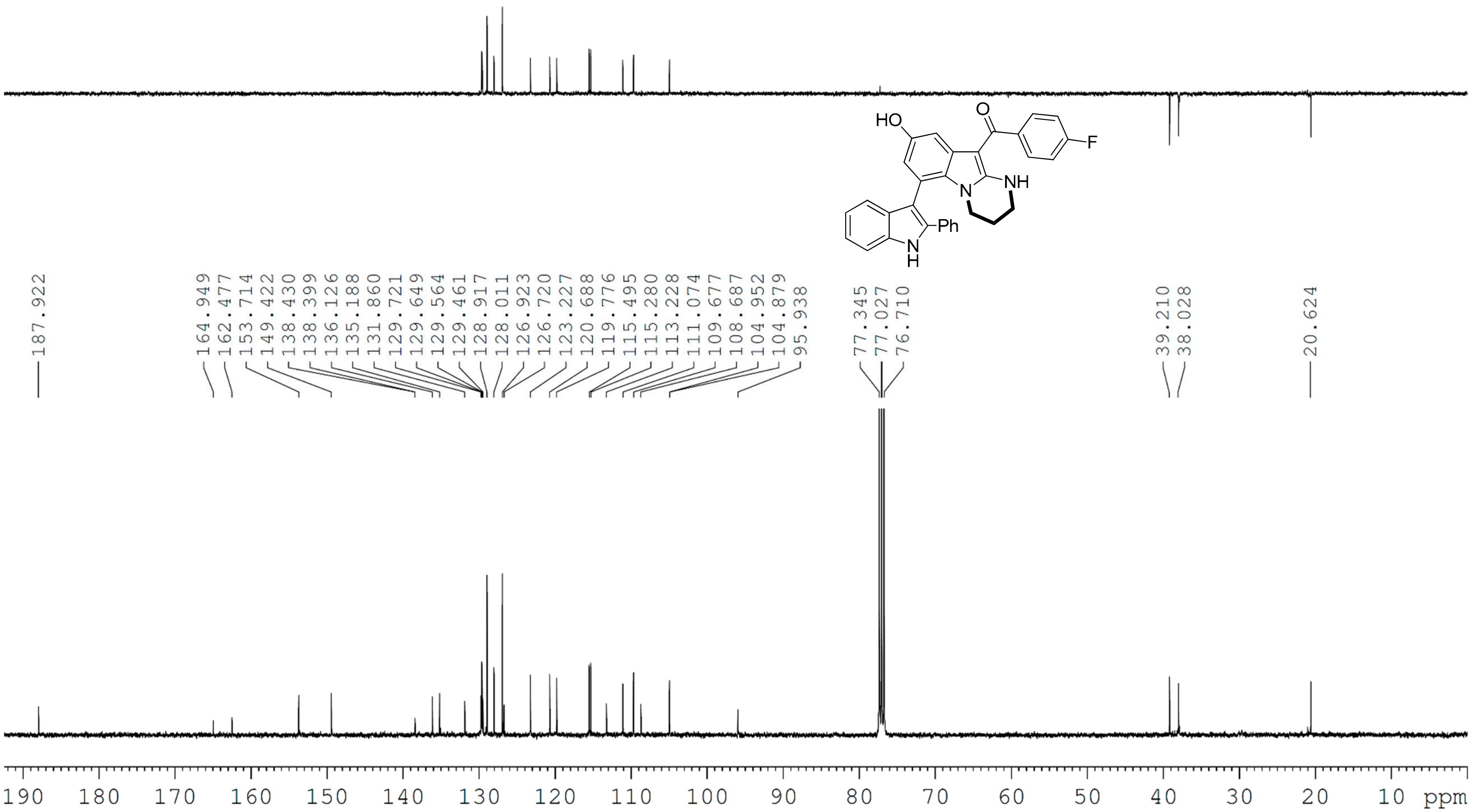

Figure S44. ^{13}C-NMR (100 MHz, $CDCl_3$) spectra of compound **3v**.

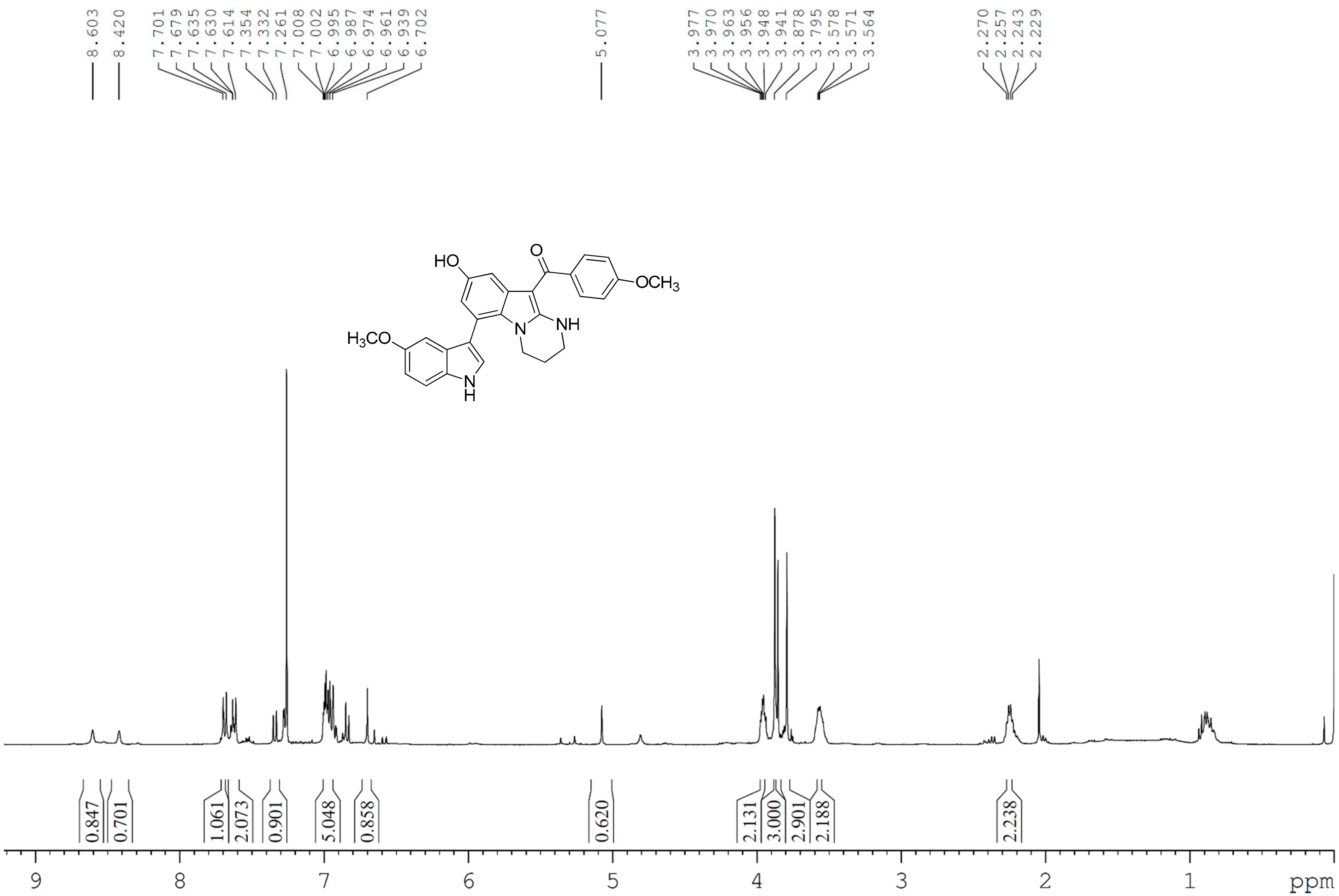

Figure S45. ^{1}H-NMR (400 MHz, $CDCl_3$) spectra of compound **3w.**

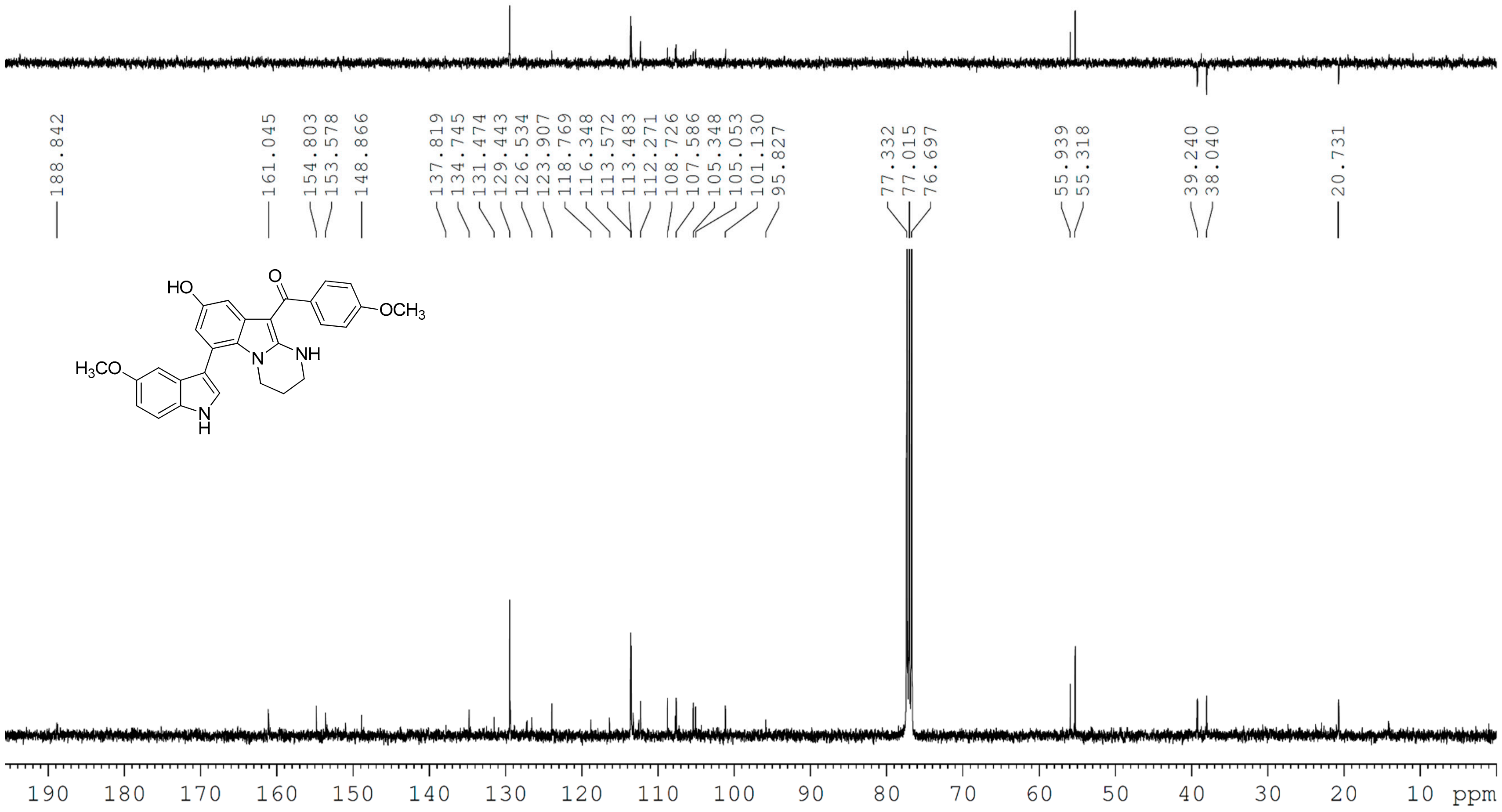

Figure S46. ^{13}C-NMR (100 MHz, $CDCl_3$) spectra of compound **3w**.

References

1. Huang, Z.-T.; Wang, M.-X. A New Route to 3*H*-1,5-Benzodiazepines and Heterocyclic Ketene Aminals from Benzoyl Substituted Ketene Dithioacetals and Diamines. *Synthesis* **1992**, *12*, 1273−1276.
2. Li, Z.-J.; Charles, D. The synthesis of Fluoroheterocyclic Ketene Aminals. *Synth. Commun.* **2001**, *31*, 527−533.
3. Chen, X.-B.; Liu, X.-M.; Huang, R.; Yan, S.-J.; Lin, J. Three-Component Synthesis of Indanone-Fused Spirooxindole Derivatives. *Eur. J. Org. Chem.* **2013**, 4607−4613.
4. Zhang, H.-B.; Liu, L.; Chen, Y.-J.; Wang, D.; Li, C.-J. “On Water”-Promoted Direct Coupling of Indoles with 1,4-Benzoquinones without Catalyst. *Eur. J. Org. Chem.* **2006**, 869−873.
5. Niu, F.; Liu, C.-C.; Cui, Z.-M.; Zhai, J.; Jiang, L.; Song, W.-G. Promotion of organic reactions by interfacial hydrogen bonds on hydroxyl group rich nano-solids. *Chem. Commun.* **2008**, 2803–2805.
